# Access to Fused Indolines with a Quaternary *N*,*N*′‑Aminal Center: Aza-Wacker-Type Cyclization for a Telescoped Reaction Sequence

**DOI:** 10.1021/acs.orglett.5c04647

**Published:** 2026-01-02

**Authors:** Sara Caselli, Amalija Golobič, Fabio Mantellini, Giacomo Mari, Gianfranco Favi

**Affiliations:** † Department of Biomolecular Sciences, Section of Chemistry and Pharmaceutical Technologies, 19044 University of Urbino “Carlo Bo”, Via Ca’ Le Suore, 2, 61029 Urbino, Italy; ‡ Faculty of Chemistry and Chemical Technology, 37663 University of Ljubljana, 1000 Ljubljana, Slovenia

## Abstract

A highly efficient one-pot sequence has been developed for the rapid construction of fused polycyclic indolines bearing a C2-*N,N*′-aminal quaternary center. The process, which employs 1,2-diaza-1,3-dienes and 2-substituted indoles as key substrates, proceeds through a Zn­(II)-catalyzed Michael addition, followed by an intramolecular Cu­(II)-catalyzed dearomative oxidative cyclization. This sequence enabling N–C­(sp^2^) bond formation via formal C­(sp^2^)–H activation, azoalkene addition, and aza-Wacker-type cyclization exhibits broad substrate tolerance and delivers unprecedented tricyclic indole architectures (namely 9a-substituted 9,9a-dihydro-1*H*-pyridazino­[3,4-*b*]­indoles) in good to excellent yields.

## Graphical abstract

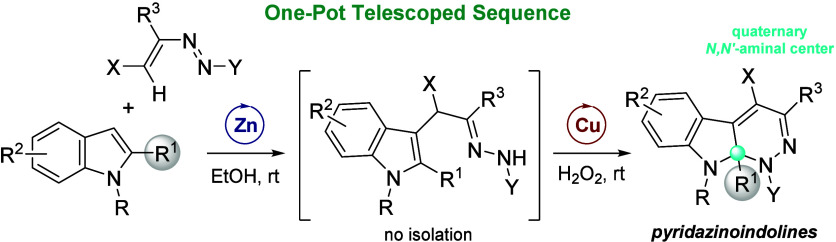

Recently, the construction of complex N-heterocycles in one step has become increasingly attractive from the viewpoint of efficiency and sustainability. Owing to their compelling biological properties and architectural complexity, polycyclic C2,C3-fused indolines continue to captivate synthetic chemists, with indole dearomatization^1^ serving as a prominent strategic approach. Among these, indol­(in)­e–pyridazine systems, which can be regarded as aza analogous (or bioisosteres) of β-carbolines, have captured our attention.^2^ In particular, quaternary *N,N*′-aminal-1*H*-pyridazino­[3,4-*b*]­indoles, which offer conformational restrictions owing to their sterically hindered nature, could enhance the affinity, selectivity, efficacy/potency, metabolic stability, and oral bioavailability of drug candidates contributing to their clinical success.^3^

Among the application of C–X oxidative coupling reactions to the synthesis of nitrogen-containing compounds, the transition metal-catalyzed amination^4^ represents a powerful tool for accessing diverse N-heterocyclic scaffolds. Within this extensive field, the intramolecular aza-Wacker cyclization (IAWC)^5^ exhibits superior utility and versatility to convert alkene-tethered amine/amide nucleophiles into alkenyl-saturated N-heterocycles (Scheme 1a). Two general metals, Pd and Cu, have been explored as productive catalysts for these transformations. For a palladium catalyst, several synthetic methods^5a,5d,5e,5g^ featuring mild reaction conditions and high efficiency have been described involving olefin aminopalladation, and β-H elimination, followed by regeneration of the Pd­(II) catalyst under oxidative conditions. With respect to the more abundant copper catalysis, various groups^5b,5c,5f^ have recently contributed to this area discovering new cheaper and sustainable protocols. Also, elegant examples of electrooxidative intramolecular coupling to achieve formal aza-Wacker cyclizations have been reported by the groups of Moeller, Hu, and Xu.^5h–5k^ Because of the high potential related to this reaction, which brings the union between nitrogen nucleophiles and alke­(y)­nyl substrates, a variety of donor and/or acceptors have been explored.

**Scheme 1:**
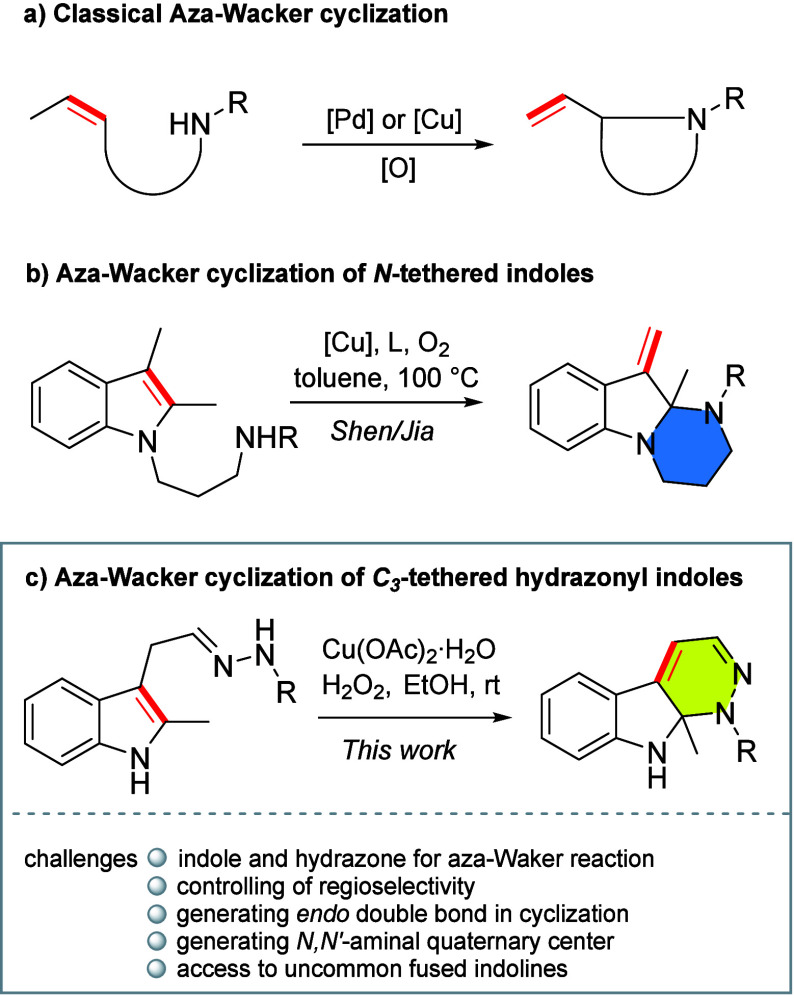
Aza-Wacker-Type Cyclizations

The group of Shen and Jia reported the only known example^5b^ of a formal intramolecular aza-Wacker-type cyclization employing indoles as π-systems and/or substrates. As shown in Scheme 1, an aza-polycyclic structure with an exocyclic C=C bond at position C3 of the indoline core is achieved as a result of deraromative amination when employing C2,C3-dialkyl N-tethered indoles. However, the type c IAWC by connecting the indole’s C2 position with a NH fragment present on the C3 side chain of indole is still lacking.

Following our sustained efforts in the construction of polycyclic N-heterocycles, especially around the indole privileged structure,^2,6^ we report herein a one-pot Michael addition/aza-Wacker cyclization strategy for the synthesis of C2 quaternary dihydro-pyridazino­[3,4-*b*]­indoles. The success of this transformation lies in combining both reactions within a single flask, thereby enabling the rapid and efficient assembly of fused indoline frameworks. Remarkably, the Cu-catalyzed intramolecular dearomative amination proceeds smoothly under mild conditions, employing benign H_2_O_2_ as the sole terminal oxidant at room temperature. By avoiding the use of reactive/hazardous peroxides and high O_2_ pressures, this protocol opens new avenues toward safer, greener, and more sustainable synthetic methodologies.

From a strategic standpoint, this oxidation cyclization operates on the indole–hydrazone substrates, which are, in turn, obtained by a Zn­(II)-catalyzed addition of indoles to azoalkenes, which our group described recently.^6a,6b^ Taken together, the addition and the oxidative cyclization constitute an efficient sequence for extending the indole nucleus by “growing” an additional fused six-membered heteroaromatic ring. To realize this, a dual orthogonal relay catalysis^7^ was developed in which two distinct and selective catalytic cycles were merged.

To verify whether our idea was achievable, we chose conveniently prepared α-indolylhydrazone^6a,6b^ **A1** as the model substrate to investigate this unprecedented IAWC reaction. When the reaction was performed with stoichiometric Cu­(OAc)_2_·H_2_O in DMA at room temperature, the desired product **3a** was obtained in 80% yield (Table 1, entry 1). Gratifyingly, a catalytic system using Cu­(OAc)_2_·H_2_O in combination of H_2_O_2_ (30 wt %, aqueous) as a sacrificial oxidant furnished product **3a** in 81% yield (entry 2). H_2_O_2_ would be the preferred oxidant because it is green (its only byproduct is water), inexpensive, and readily available.^8^ After the evaluation of a series of Lewis acid catalysts (Cu­(OAc)_2_·H_2_O, Cu­(OTf)_2_, CuCl_2_, CuSO_4_, ZnCl_2_, Zn­(OAc)_2_, CuO, AgOAc, Cu, CuCl, Cu_2_O, and FeCl_3_) in combination with H_2_O_2_ as an oxidant and solvents (DMA, EtOH, EtOAc, ACN, THF, and DCM), the best conditions were established as follows: **A1** (0.3 mmol), Cu­(OAc)_2_·H_2_O (5%), and H_2_O_2_ (3 equiv) in solvent EtOH (2 mL) at rt for 5 h (entries 3–18, Table 1). It should be noted that decreasing the amount of copper source from 20% to 5% did not significantly erode the yield (entries 19 and 20, Table 1). Control experiments established that the IAWC requires the assistance of both the catalyst and the oxidant. In the absence of the catalyst, an only 13% yield of product **3a** was obtained (entry 21), while omission of H_2_O_2_ led to a reduced yield of 46% (entry 22).

**Table 1:**
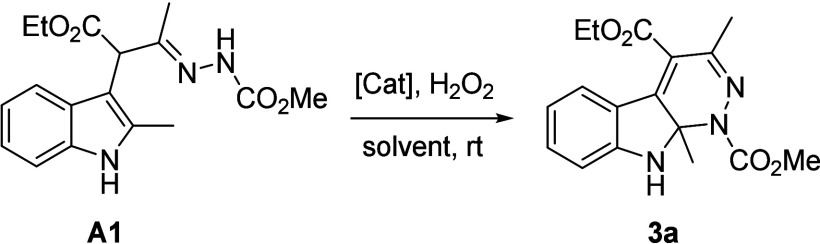
Optimization of the Cyclization Step^a^

entry	catalyst	solvent	time (h)	yield (%)^b^
1	Cu(OAc)_2_·H_2_O^c^	DMA	0.3	80
2	Cu(OAc)_2_·H_2_O	DMA	5	81
3	Cu(OAc)_2_·H_2_O	EtOH	0.4	92
4	Cu(OAc)_2_·H_2_O	AcOEt	3.5	90
5	Cu(OAc)_2_·H_2_O	CH_3_CN	20	19
6	Cu(OAc)_2_·H_2_O	THF	0.5	88
7	Cu(OAc)_2_·H_2_O	DCM	20	44
8	Cu(OTf)_2_	EtOH	20	54
9	CuCl_2_	EtOH	1.3	46
10	CuSO_4_	EtOH	2	50^d^
11	CuO	EtOH	>150	68^d^
12	Cu	EtOH	20	48^d^
13	CuCl	EtOH	2	51^d^
14	Cu_2_O	EtOH	20	78^d^
15	ZnCl_2_	EtOH	>100	43
16	Zn(OAc)_2_	EtOH	>150	8 (25)^e^
17	AgOAc	EtOH	>150	13 (57)^e^
18	FeCl_3_	EtOH	48	25^c^
19	Cu(OAc)_2_·H_2_O^g^	EtOH	2	89
20	Cu(OAc)_2_·H_2_O^h^	EtOH	2	87
21	–	EtOH	20	13 (80)^e^
22	Cu(OAc)_2_·H_2_O^f^	EtOH	>24	46^d^

a Reaction conditions: **A1** (0.3 mmol), a catalyst (20 mol %), and H_2_O_2_ (40 wt %, aqueous, 3 equiv) in 2 mL of a solvent at rt for the indicated time.

b Isolated yields.

c Using 1 equiv of Cu­(OAc)_2_·H_2_O without H_2_O_2_.

d Hydrazine tautomeric byproduct **A1′** (see the Supporting Information) was also recovered.

e Recovered starting material (**A1**) is given in parentheses.

f Without H_2_O_2_.

g Using 10 mol % Cu­(OAc)_2_·H_2_O.

h Using 5 mol % Cu­(OAc)_2_·H_2_O.

Afterward, we focused on assembling quaternary *N,N′*-aminal-1*H*-pyridazino­[3,4-*b*]­indole **3a** directly from 2-substituted indole **1a** and 1,2-diaza-1,3-diene **2a**. To this end, a one-pot^9^ protocol in which α-indolylhydrazone intermediate **A1** was preformed upon exposure to the ZnCl_2_ catalyst (10%) in EtOH at rt (TLC monitoring) and an IAWC process was subsequently employed by adding Cu­(OAc)_2_·H_2_O (5%) and H_2_O_2_ (3 equiv) was carried out. To our delight, the combination of the two steps in the same reaction vessel provided the desired product **3a** in 75% yield. With this promising result, the scope and limitation of this telescoped one-flask transformation by using various 2-substituted indoles **1** and 1,2-diaza-1,3-dienes **2** were then investigated (Scheme 2). Various azoalkene substrates (R^4^ = CO_2_Et, CO_2_Me, CO_2_-*i*-Pr, CO_2_-*t*-Bu, CO_2_Bn, CO_2_allyl, CO_2_(CH_2_)_2_OMe, CONMe_2_, CONH_2_, PO­(OMe)_2_, or Ph; R^5^ = Me, *i*-Pr, or Pr; R^6^ = OMe, OEt, O-*t*-Bu, OBn, NH_2_, or NHPh) were found to be compatible with 2-methylindole **1a** delivering pyridazinoindolines **3a–r** in 20**–**75% yields (Scheme 2).

**Scheme 2:**
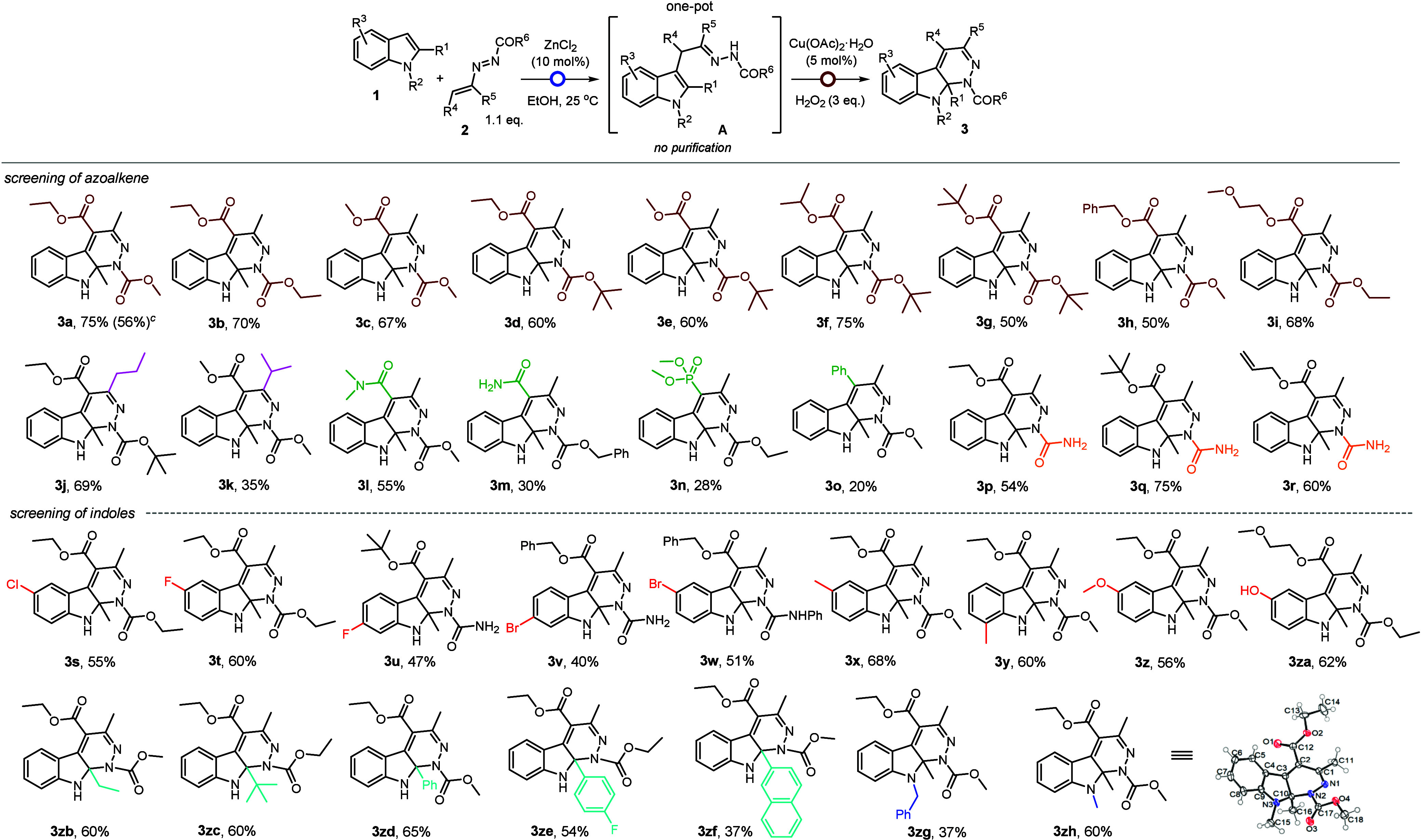
Substrate Scope of the Telescoped Michael Addition/Intramolecular Aza-Wacker-Type Cyclization^a^,^b^

a Reactions performed with **1** (0.5 mmol), **2** (0.55 mmol), and ZnCl_2_ (10 mol %) in EtOH (2 mL) at rt for 0.25–4 h and then Cu­(OAc)_2_·H_2_O (5 mol %) and H_2_O_2_ (40 wt %, aqueous, 3 equiv) at rt for 0.25–12 h.
b Isolated yields.
c Gram scale synthesis.

While 4-alkoxycarbonyl-DDs (**2a–j**) worked well, a fairly lower efficiency of cyclization (**3k**, 35% yield) was observed in the case of DD with an isopropyl group at position C3. Those with amido, dimethoxyphosphoryl, or phenyl groups at position C4 of the DDs also gave cyclized products **3l–o** in moderate yields. The effect of the N-terminal protective group on DDs was irrelevant since amido products **3p–r** were also tolerated under the conditions. The reaction scope of this telescoped Michael addition/intramolecular aza-Wacker-type cyclization was further explored with a series of indoles **1**. Incorporation of various electron-donating and electron-withdrawing (-F, -Cl, -Br, -OMe, and -OH, and -Me) into the benzene ring in the indole nucleus does not affect cyclization efficiency (**3s–za**). Remarkably, in addition to 2-methylindoles **1a–j**, the protocol worked smoothly for indoles containing ethyl (**1k**), *tert*-butyl (**1l**), phenyl (**1m**), *p*-fluorophenyl (**1n**), and naphthyl (**1o**) moieties. Indoles substituted with N-protecting groups such as benzyl (**1p**), and methyl (**1q**) were also competent substrates for this synthetic protocol. The structure of products was established by NMR and HRMS analysis. In addition, the structure of **3zh** was unambiguously elucidated by single-crystal X-ray analysis (Scheme 2, CCDC 2467779).

The applicability of this two-step, one-pot protocol was demonstrated in a scale-up reaction between **1a** and **2a** under the optimized conditions. The transformation proceeded fairly well to afford resultant product **3a** in 59% yield (Scheme 3a). Compounds **3a** and **3d** proved to be useful intermediates for synthetic derivatizations. Dichlorination of **3a** was achieved by treatment with TCCA^10^ in DCM at room temperature (Scheme 3b). Furthermore, exposure of Boc-protected pyridazinoindoline **3d** to BF_3_·Et_2_O^11^ in DCM afforded intriguing indolidene–pyrazolone **5a** in 48% yield (Scheme 3c). This occurrence can be rationalized by an initial *N*-Boc deprotection followed by a ring-opening/ring-closure sequence.^12^

**Scheme 3:**
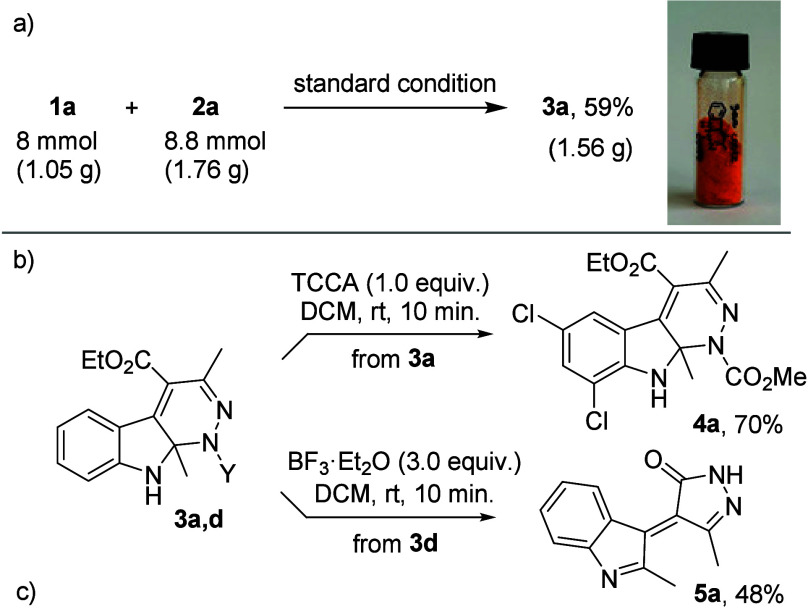
Gram Scale Synthesis of **3a** and Derivatizations of **3a** and **3d**

Thereafter, some mechanistic studies and control experiments were conducted to gain a better mechanistic understanding of the key intramolecular Cu­(II)-catalyzed dearomative oxidative/aza-Wacker-type cyclization (Scheme 4).

**Scheme 4:**
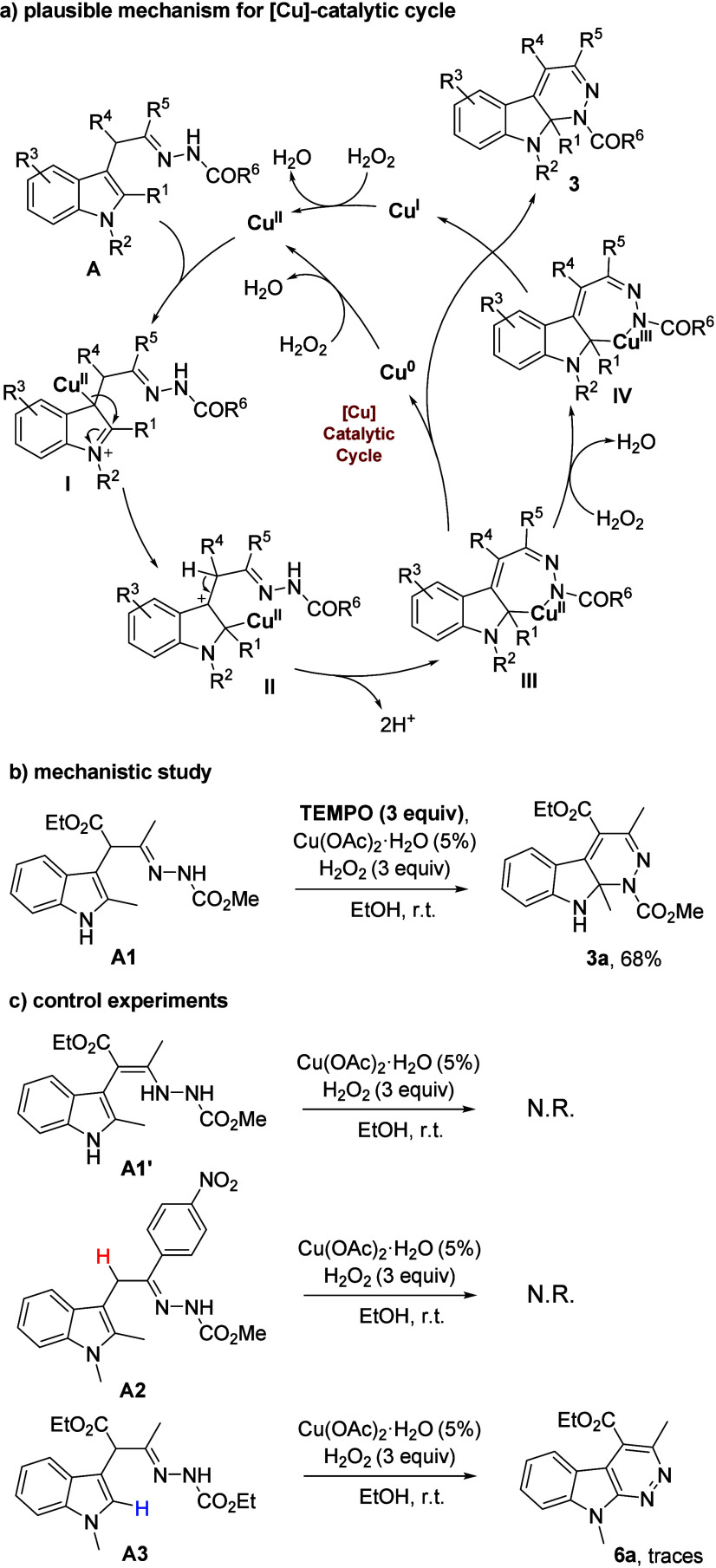
(a) Possible Reaction Mechanism for IAWC, (b) Mechanistic Study, and (c) Control Experiments

First, performing the IAWC reaction under the standard conditions in the presence of 3.0 equiv of a radical scavenger (TEMPO = 2,2,6,6-tetramethylpiperidin-1-oxyl) did not affect the formation of **3a**, thereby excluding the possibility of a free radical pathway (Scheme 4b). Next, when the hydrazine tautomeric form of **A1** (**A1′**) was subjected to the standard conditions, no formation of **3a** was observed (**A1′** remained intact), indicating that this tautomer is not an active intermediate in the transformation. Furthermore, when using **A2** as the substrate, no dearomative cycloamination occurred. While the presence of an acidic proton at the α-position of the hydrazone is crucial for the reaction to proceed (and for the production of byproduct **A1′**), we surmise that substrates lacking such an acidic hydrogen may be unable to participate in the proton elimination step of benzylic carbocation species **II** (*vide infra*), thus hindering the oxidation process. Finally, oxidative cyclization was performed on a C2-unsubstituted substrate (**A3**). As expected, aromatic cinnoline **6a**,^2a^ albeit recovered only in trace amounts, was formed via oxidative cycloamination followed by further aromatization through an initial CH/NH tautomerization.

Based on the above results and literature reports,^4c,6a,13^ a tentative catalytic cycle for the aza-Wacker-type cyclization is proposed using a Cu^I^/Cu^II^/Cu^III^ catalytic cycle (Scheme 4). First, electrophilic cupration of α-indolylhydrazone **A** with Cu­(II) occurs leading to intermediate **I**, which undergoes 1,2-migratory metalation to give intermediate **II**. Cleavage of the C–H bond with concomitant Cu nitrogen-atom association/insertion gives seven-membered intermediate **III**, which is involved in the oxidation of Cu­(II) to form Cu­(III) intermediate **IV** by H_2_O_2_. Reductive elimination furnishes the desired cyclization product **3** and release of Cu­(I) that is then oxidized to regenerate active catalytic Cu­(II) species. As an alternative pathway (Cu^II^/Cu^0^ catalytic cycle), Cu­(II) intermediate **III** might be converted into the final product by releasing the Cu(0), which would be reoxidized to Cu­(II) by H_2_O_2_ to complete the catalytic cycle.

The formation of byproduct **A1′** occasionally isolated from the reaction media (see Table 1) could arise from a 1,3-H shift that precedes the C–H bond cleavage of benzylic carbocation intermediate **II** (not shown).

To summarize, we have investigated a novel and efficient intramolecular Cu-catalyzed aza-Wacker-type cyclization that enables the synthesis of fused polycyclic indoline systems. The feasibility of integrating this dearomative amination process with a Zn-catalyzed Michael addition between 1,2-diaza-1,3-dienes and 2-substituted indoles has been successfully demonstrated in a telescopic sequence, offering clear advantages in terms of operational simplicity and sustainability. Moreover, the unique tricyclic 6/5/6 framework of product **3** featuring a C2-*N,N*′-aminal quaternary center^14,15^ allows it to “escape from flatland” by incorporating an sp^3^-hybridized C atom, a structural characteristic that could attract medicinal chemists for the design of novel drug candidates.

## Supplementary Material





## Data Availability

The data underlying this study are available in the published article and its .

## Notes

Experimental procedures, characterization data, X-ray data of compound **3zh**, and copies of ^1^H, ^13^C, and ^19^F NMR spectra (PDF)
FAIR data, including the primary NMR FID files, for compounds **3a**–**zh**, **4a**, **5a**, **A**, and **A1′** (ZIP)
