# Supplementary material for: Access to Fused Indolines with a Quaternary N,N′‑Aminal Center: Aza-Wacker-Type Cyclization for a Telescoped Reaction Sequence
Source: Org Lett. 2026 Jan 2;28(2):634–9. doi: 10.1021/acs.orglett.5c04647 (PMC12814564; doi:10.1021/acs.orglett.5c04647)

Access to Fused Indolines with a Quaternary *N,N′*-Aminal Center: Aza-Wacker-Type Cyclization for a Telescoped Reaction Sequence

*Sara Caselli,[a] Amalija Golobič,[b] Fabio Mantellini,[a] Giacomo Mari,[a] and Gianfranco Favi,[a,]**

[a]Department of Biomolecular Sciences, Section of Chemistry and Pharmaceutical Technologies, University of Urbino "Carlo Bo", Via Cà Le Suore, 2, 61029 Urbino, Italy

[b]Faculty of Chemistry and Chemical Technology, University of Ljubljana, 1000 Ljubljana, Slovenia

Email: gianfranco.favi@uniurb.it

Table of Contents

1. **General remarks** S2
2. **Substrates involved in the manuscript** S3
3. **Synthesis and characterization of α-(indol-3-yl)hydrazo(i)ne A1 and A1'** S4–S5
4. **Synthesis and characterization of 1,3-diaryl imidazolidines 3a-zh** S5–S22
    4.1 Procedures for the synthesis of **3a-zh** S5
    4.2 Characterization of **3a-zh** S6–S22
5. **Preparation method for crystal growth of compound 3zh** S23
6. **Procedure for scale-up reaction** S23
7. **Derivatization of 3a,d** S23–S24
8. **X-ray structure of compound 3zh** S25–S26
9. **References** S27
10. **Copies of ^{1}H and ^{13}C NMR spectra** S28–S70

1. General Remarks

All the commercially available reagents and solvents were used without further purification. Indoles **1a–o, 1q** are commercially available. *N*-Benzylindole **1p** was prepared from corresponding commercially available *N*H-indole following literature procedures.[1] 1,2-Diaza-1,3-dienes (DDs) **2a–2r** were synthesized from the corresponding hydrazones following literature procedures.[2] α-(Indol-3-yl)hydrazone intermediate (**A1**) was synthesized according to previously our reported method.[3] Chromatographic purification of compounds was carried out on silica gel (60–200 μm). TLC analysis was performed on pre-loaded (0.25 mm) glass supported silica gel plates (Kieselgel 60); compounds were visualized by exposure to UV light and by dipping the plates in 1% $Ce(SO_4)\cdot 4H_2O$, 2.5% $(NH_4)_6Mo_7O_{24}\cdot 4H_2O$ in 10% sulphuric acid followed by heating on a hot plate. All ^{1}H NMR, ^{13}C NMR and ^{19}F NMR spectra were recorded at 400, 101 and 376 MHz using DMSO-d_6 as solvent on a Bruker Ultrashield 400 spectrometer (Bruker, Billerica, MA, USA). Chemical shift (δ scale) are reported in parts per million (ppm) relative to the central peak of the solvent and are sorted in descending order within each group. The following abbreviations are used to describe peak patterns where appropriate: s = singlet, bs = broad singlet, d = doublet, dd = doublet of doublet, dt = doublet of triplet, t = triplet, td = triplet of doublet, dq = doublet of quartet, q = quartet, sept = septet and m = multiplet. All coupling constants (J value) are given in Hertz [Hz]. High-resolution mass spectral (HRMS) analyses were performed using Orbitrap Exploris 240 Mass Spectrometers (Thermo Scientific) equipped with an ESI source. Melting points were determined in open capillary tubes and are uncorrected.

2. Substrates involved in the manuscript.

2.1 *Indoles*

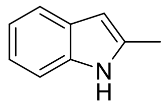
1a

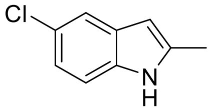
1b

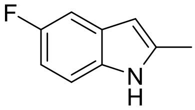
1c

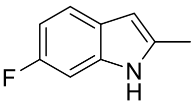
1d

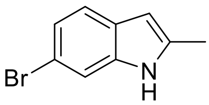
1e

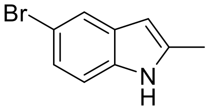
1f

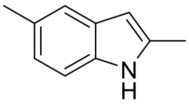
1g

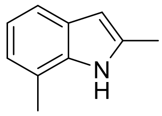
1h

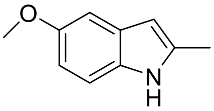
1i

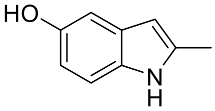
1j

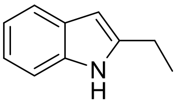
1k

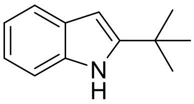
1l

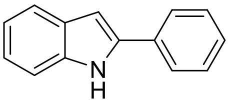
1m

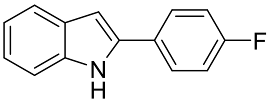
1n

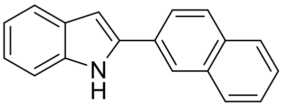
1o

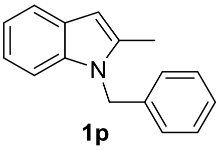
1p

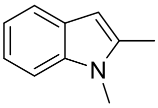
1q

2.2 *Azoalkenes*

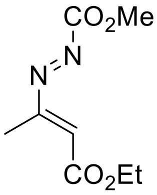
2a

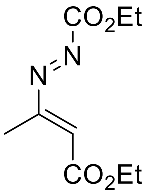
2b

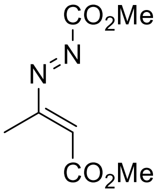
2c

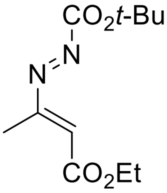
2d

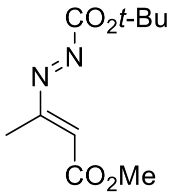
2e

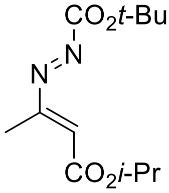
2f

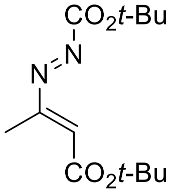
2g

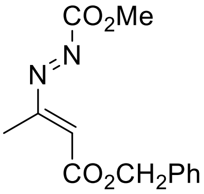
2h

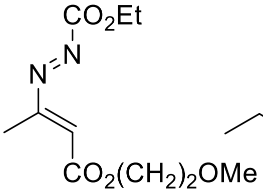
2i

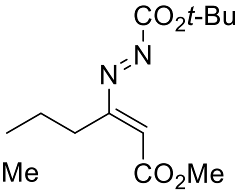
2j

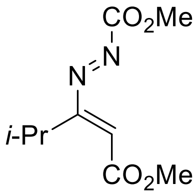
2k

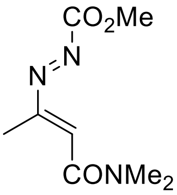
2l

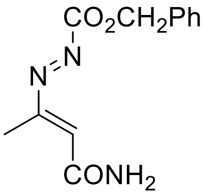
2m

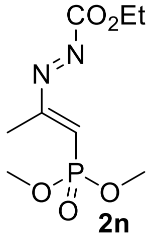
2n

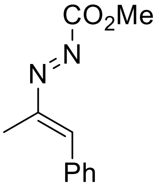
2o

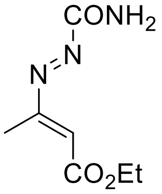
2p

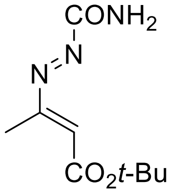
2q

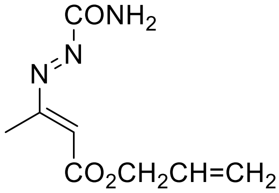
2r

3. Synthesis of α-(indol-3-yl)hydrazone A1.[3]

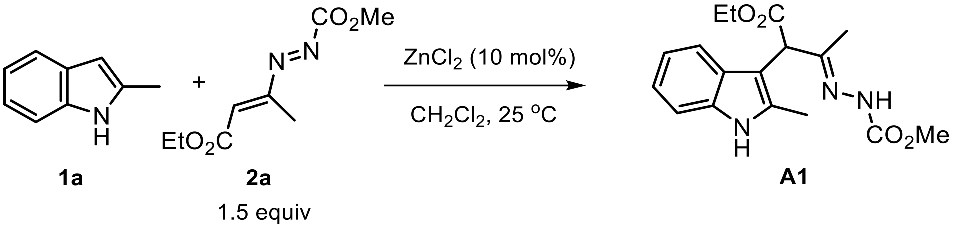

To a stirred mixture of indole **1a** (1 mmol) and azoalkene **2a** (1.5 mmol, 1.5 equiv) in dichloromethane (4 mL), $ZnCl_2$ (13.6 mg, 0.1 mmol, 10 mol %) was added. After the disappearance of indole (3h, TLC check), the solvent was removed and the crude mixture was purified by column chromatography on silica gel to afford, after crystallization, the α-(indol-3-yl)hydrazone **A1** (95% yield).

*3.1 Characterization of α-(indol-3-yl)hydrazone (**A1**)*:

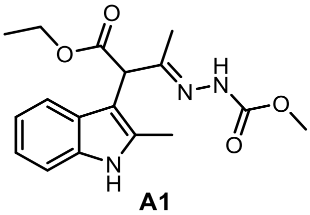

A1

Methyl 2-(4-ethoxy-3-(2-methyl-1*H*-indol-3-yl)-4-oxobutan-2-ylidene)hydrazinecarboxylate (A1): compound **A1** was isolated by column chromatography (cyclohexane/ethyl acetate 7:3) in 95% yield (314.8 mg); white solid; mp: 155–157 °C. **^{1}H NMR (400 MHz, DMSO-d_6)** δ 10.99 (s, 1H), 9.84 (s, 1H), 7.33 (d, J = 7.9 Hz, 1H), 7.25 (d, J = 7.9 Hz, 1H), 7.00 (td, J = 7.9 Hz, J = 1.1 Hz, 1H), 6.92 (td, J = 7.9 Hz, J = 1.1 Hz, 1H), 4.75 (s, 1H), 4.29–4.02 (m, 2H), 3.66 (s, 3H), 2.30 (s, 3H), 1.73 (s, 3H), 1.17 (t, J = 7.1 Hz, 3H). **^{13}C{^{1}H} NMR (101 MHz, DMSO-d_6)** δ 171.1, 154.6, 151.8, 135.0, 134.0, 127.5, 120.1, 118.6, 118.0, 110.6, 104.2, 60.3, 51.7, 50.9, 15.0, 14.1, 11.6. **HRMS (ESI-Orbitrap, *m/z*):** $[M+H]^+$ Calcd for $C_{17}H_{22}N_3O_4^+$ 332.1605; Found 332.1592.

*Characterization of hydrazine tautomeric form of α-(indol-3-yl)hydrazone (**A1'**)*

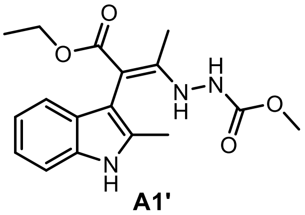

A1'

Methyl 2-(4-ethoxy-3-(2-methyl-1*H*-indol-3-yl)-4-oxobut-2-en-2-yl)hydrazine-1-carboxylate (A1'): compound **A1'** was isolated by column chromatography (cyclohexane/ethyl acetate 5:5); dark yellow oil. **^{1}H NMR (400 MHz, DMSO-*d*$_6$)** δ 10.87 (s, 1H), 10.01 (s, 1H), 7.43 (d, J = 8.0 Hz, 1H), 7.20 (d, J = 8.0 Hz, 1H), 6.96 (td, J = 8.0 Hz, J = 0.9 Hz, 1H), 6.86 (td, J = 8.0 Hz, J = 0.9 Hz, 1H), 5.94 (s, 1H), 4.20 (dq, J = 10.7, J = 7.1 Hz, 1H), 3.94 (dq, J = 10.7, J = 7.1 Hz, 1H), 3.68 (s, 3H), 2.31 (s, 3H), 1.74 (s, 3H), 1.16 (t, J = 7.1 Hz, 3H). **^{13}C NMR (101 MHz, DMSO-*d*$_6$)** δ 172.9, 154.6, 154.0, 134.8, 133.6, 127.4, 120.2, 119.8, 118.3, 110.2, 108.5, 80.9, 60.6, 51.8, 13.9, 13.2, 13.1. **HRMS (ESI-Orbitrap, *m/z*):** $[M+H]^+$ Calcd for $C_{17}H_{22}N_3O_4^+$ 332.1605; Found 332.1619.

4. Synthesis of products 3.

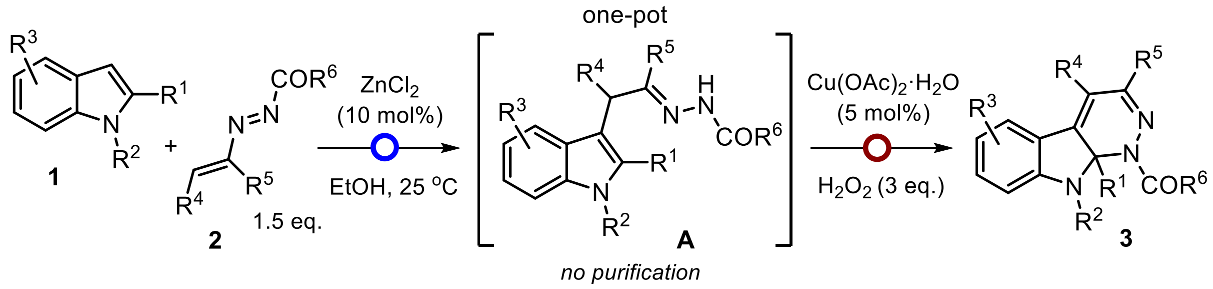

To a stirred mixture of indole **1** (0.5 mmol) and azoalkene **2** (1.5 eq.) in ethanol (2 mL), $ZnCl_2$ (10 mol%) was added. Upon disappearance of **1** (0.25–4 h, TLC check), to the same reaction mixture $Cu(OAc)_2{\cdot}H_2O$ (5 mol%) and H_2O_2 (40 wt%, aqueous, 3 eq.) were sequentially added. After TLC showed complete consumption of intermediate **A** (0.25–12 h, TLC check), the solvent was removed, and the crude mixture was purified by column chromatography on silica gel to afford the product **3** (30–75% yields).

*4.1 Characterization of pyridazinoindolines **3a–3zh***:

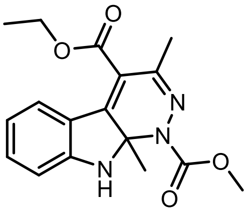

3a

4-Ethyl 1-methyl 3,9a-dimethyl-9,9a-dihydro-1*H*-pyridazino[3,4-*b*]indole-1,4-dicarboxylate (3a): compound **3a** was isolated by column chromatography (cyclohexane/ethyl acetate 7:3) in 75% yield (172 mg); orange solid; mp: 116–118 °C. **^{1}H NMR (400 MHz, DMSO-*d*$_6$)** δ 7.51 (d, *J* = 7.8 Hz, 1H), 7.41 (s, 1H), 7.31 (td, *J* = 8.1 Hz, *J* = 1.2 Hz, 1H), 6.89 (d, *J* = 8.1 Hz, 1H), 6.75 (td, *J* = 7.8 Hz, *J* = 0.9 Hz, 1H), 4.43–4.26 (m, 2H), 3.79 (s, 3H), 2.12 (s, 3H), 1.30 (t, *J* = 7.1 Hz, 3H), 1.22 (s, 3H). **^{13}C{^{1}H} NMR (101 MHz, DMSO-*d*$_6$)** δ 165.5, 154.9, 152.8, 144.6, 143.5, 134.0, 125.6, 118.9, 118.2, 112.6, 111.6, 74.8, 61.5, 53.2, 19.6, 19.1, 13.9. **HRMS (ESI-Orbitrap, *m/z*):** $[M+H]^+$ Calcd for $C_{17}H_{20}N_3O_4^+$ 330.1448; Found 330.1438.

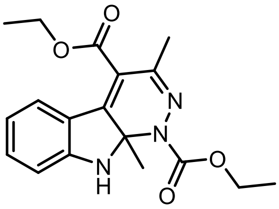

3b

Diethyl 3,9a-dimethyl-9,9a-dihydro-1*H*-pyridazino[3,4-*b*]indole-1,4-dicarboxylate (3b): compound **3b** was isolated by column chromatography (cyclohexane/ethyl acetate 7:3) in 70% yield (120 mg); orange oil. **^{1}H NMR (400 MHz, DMSO-*d*$_6$)** δ 7.51 (d, *J* = 7.8 Hz, 1H), 7.38 (s, 1H), 7.31 (td, *J* = 8.1 Hz, *J* = 1.2 Hz, 1H), 6.90 (d, *J* = 8.1 Hz, 1H), 6.76 (td, *J* = 7.8 Hz, *J* = 0.9 Hz, 1H), 4.45–4.08 (m, 4H), 2.12 (s, 3H), 1.30 (t, *J* = 7.2 Hz, 3H), 1.28 (t, *J* = 7.1 Hz, 3H), 1.22 (s, 3H). **^{13}C{^{1}H} NMR (101 MHz, DMSO-*d*$_6$)** δ 165.5, 154.4, 152.7, 144.6, 143.4, 133.9, 125.5, 118.9, 118.2, 112.6, 111.6, 74.8, 62.0, 61.5, 19.7, 19.1, 14.4, 13.9. **HRMS (ESI-Orbitrap, *m/z*):** $[M+H]^+$ Calcd for $C_{18}H_{22}N_3O_4^+$ 344.1605; Found 344.1609.

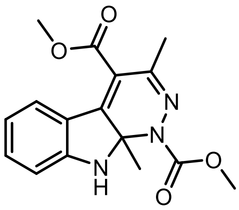

3c

Dimethyl 3,9a-dimethyl-9,9a-dihydro-1*H*-pyridazino[3,4-*b*]indole-1,4-dicarboxylate (3c): compound **3c** was isolated by column chromatography (cyclohexane/ethyl acetate 7:3) in 67% yield (105 mg); dark yellow solid; mp: 145–147 °C. **^{1}H NMR (400 MHz, DMSO-*d*$_6$)** δ 7.47 (d, *J* = 7.6 Hz, 1H), 7.44 (s, 1H), 7.31 (td, *J* = 8.1 Hz, *J* = 1.1 Hz, 1H), 6.89 (d, *J* = 8.1 Hz, 1H), 6.75 (td, *J* = 7.6 Hz, *J* = 0.8 Hz, 1H), 3.88 (s, 3H), 3.79 (s, 3H), 2.11 (s, 3H), 1.22 (s, 3H). **^{13}C{^{1}H} NMR (101 MHz, DMSO-*d*$_6$)** δ 166.0, 154.9, 152.8, 144.9, 143.5, 134.0, 125.6, 119.0, 118.1, 112.2, 111.7, 74.8, 53.2, 52.5, 19.6, 19.1. **HRMS (ESI-Orbitrap, *m/z*):** $[M+H]^+$ Calcd for $C_{16}H_{18}N_3O_4^+$ 316.1292. Found 316.1285.

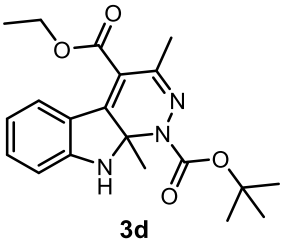

3d

1-(*tert*-Butyl) 4-ethyl 3,9a-dimethyl-9,9a-dihydro-1*H*-pyridazino[3,4-*b*]indole-1,4-dicarboxylate (3d): compound **3d** was isolated by column chromatography (cyclohexane/ethyl acetate 7:3) in 60% yield (155 mg); orange solid; mp: 104–106 °C. **^{1}H NMR (400 MHz, DMSO-*d*$_6$)** δ 7.49 (d, *J* = 7.9 Hz, 1H), 7.30 (td, *J* = 8.1 Hz, *J* = 1.2 Hz, 1H), 7.27 (s, 1H), 6.90 (d, *J* = 8.1 Hz, 1H), 6.73 (td, *J* = 7.9 Hz, *J* = 0.9 Hz, 1H), 4.39–4.30 (m, 2H), 2.11 (s, 3H), 1.51 (s, 9H), 1.30 (t, *J* = 7.1 Hz, 3H), 1.20 (s, 3H). **^{13}C{^{1}H} NMR (101 MHz, DMSO-*d*$_6$)** δ 165.6, 153.4, 152.7, 144.3, 142.8, 133.7, 125.5, 118.8, 118.2, 112.6, 111.7, 81.5, 74.8, 61.4, 27.9, 19.8, 18.9, 13.9. **HRMS (ESI-Orbitrap, *m/z*):** $[M+H]^+$ Calcd for $C_{20}H_{26}N_3O_4^+$ 372.1918; Found 372.1927.

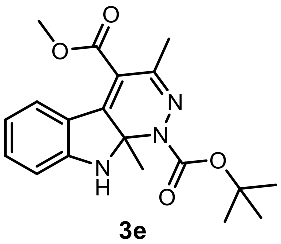

3e

1-(*tert*-Butyl) 4-methyl 3,9a-dimethyl-9,9a-dihydro-1*H*-pyridazino[3,4-*b*]indole-1,4-dicarboxylate (3e): compound **3e** was isolated by column chromatography (cyclohexane/ethyl acetate 7:3) in 60% yield (150 mg); orange oil. **^{1}H NMR (400 MHz, DMSO-*d*$_6$)** δ 7.45 (d, *J* = 7.9 Hz, 1H), 7.30 (td, *J* = 8.3 Hz, *J* = 1.2 Hz, 1H), 7.29 (s, 1H), 6.90 (d, *J* = 8.3 Hz, 1H), 6.73 (td, *J* = 7.9 Hz, *J* = 0.9 Hz, 1H), 3.88 (s, 3H), 2.09 (s, 3H), 1.51 (s, 9H), 1.20 (s, 3H). **^{13}C{^{1}H} NMR (101 MHz, DMSO-*d*$_6$)** δ 166.1, 153.4, 152.8, 144.6, 142.8, 133.8, 125.5, 118.9, 118.2, 112.3, 111.7, 81.5, 74.8, 52.4, 27.9, 19.8, 18.9. **HRMS (ESI-Orbitrap, *m/z*):** $[M+H]^+$ Calcd for $C_{19}H_{24}N_3O_4^+$ 358.1761; Found 358.1758.

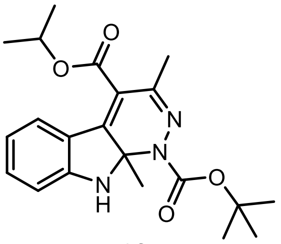

3f

1-(*tert*-Butyl) 4-isopropyl 3,9a-dimethyl-9,9a-dihydro-1*H*-pyridazino[3,4-*b*]indole-1,4-dicarboxylate (3f): compound **3f** was isolated by column chromatography (cyclohexane/ethyl acetate 7:3) in 75% yield (144.5 mg); yellow solid; mp: 157–159 °C. **^{1}H NMR (400 MHz, DMSO-*d*$_6$)** δ 7.51 (d, *J* = 7.9 Hz, 1H), 7.29 (td, *J* = 8.3 Hz, *J* = 1.2 Hz, 1H), 7.25 (s, 1H), 6.89 (td, *J* = 7.9 Hz, *J* = 0.9 Hz, 1H), 6.74 (d, *J* = 8.3 Hz, 1H), 5.18 (sept, *J* = 6.2 Hz, 1H), 2.10 (s, 3H), 1.51 (s, 9H), 1.34 (d, *J* = 6.2 Hz, 3H), 1.27 (d, *J* = 6.2 Hz, 3H), 1.20 (s, 3H). **^{13}C{^{1}H} NMR (101 MHz, DMSO-*d*$_6$)** δ 165.1, 153.3, 152.6, 143.9, 142.8, 133.7, 125.4, 118.8, 118.3, 113.0, 111.6, 81.4, 74.8, 69.3, 27.9, 21.5, 21.3, 19.8, 18.9. **HRMS (ESI-Orbitrap, *m/z*):** $[M+H]^+$ Calcd for $C_{21}H_{28}N_3O_4^+$ 386.2074. Found 386.2063.

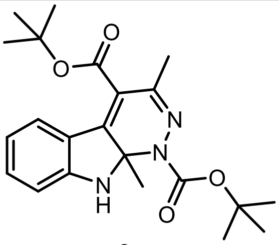

3g

di-*tert*-Butyl 3,9a-dimethyl-9,9a-dihydro-1*H*-pyridazino[3,4-*b*]indole-1,4-dicarboxylate (3g): compound **3g** was isolated by column chromatography (cyclohexane/ethyl acetate 7:3) in 50% yield (100 mg); orange solid; mp: 131–133 °C. **^{1}H NMR (400 MHz, DMSO-*d*$_6$)** δ 7.52 (d, *J* = 7.9 Hz, 1H), 7.28 (td, *J* = 8.3 Hz, *J* = 1.2 Hz, 1H), 7.21 (s, 1H), 6.89 (d, *J* = 8.3 Hz, 1H), 6.74 (td, *J* = 7.9 Hz, *J* = 0.8 Hz, 1H), 2.12 (s, 3H), 1.54 (s, 9H), 1.51 (s, 9H), 1.19 (s, 3H). **^{13}C{^{1}H} NMR (101 MHz, DMSO-*d*$_6$)** δ 165.0, 153.4, 152.4, 143.0, 142.9, 133.6, 125.2, 118.9, 118.6, 114.3, 111.7, 82.6, 81.5, 74.8, 28.0, 27.7, 19.8, 19.0. **HRMS (ESI-Orbitrap, *m/z*):** $[M+H]^+$ Calcd for $C_{22}H_{30}N_3O_4^+$ 400.2231. Found 400.2233.

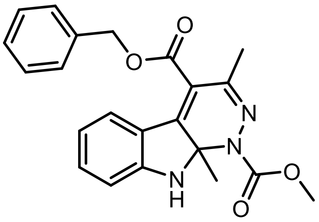

3h

4-Benzyl 1-methyl 3,9a-dimethyl-9,9a-dihydro-1*H*-pyridazino[3,4-*b*]indole-1,4-dicarboxylate (3h): compound **3h** was isolated by column chromatography (cyclohexane/ethyl acetate 7:3) in 50% yield (98 mg); orange oil. **^{1}H NMR (400 MHz, DMSO-*d*$_6$)** δ 7.54–7.35 (m, 7H), 7.29 (td, *J* = 8.3 Hz, *J* = 1.2 Hz, 1H), 6.87 (d, *J* = 8.3 Hz, 1H), 6.64 (td, *J* = 8.0 Hz, J = 0.9 Hz, 1H), 5.41 (d, *J* = 12.0 Hz, 1H), 5.36 (d, *J* = 12.0 Hz, 1H), 3.78 (s, 3H), 2.07 (s, 3H), 1.20 (s, 3H). **^{13}C{^{1}H} NMR (101 MHz, DMSO-*d*$_6$)** δ 165.4, 154.9, 152.8, 144.8, 143.4, 135.1, 134.0, 129.1, 128.6 (2C), 125.7, 118.8, 118.1, 112.2, 111.6, 74.8, 67.2, 53.2, 19.6, 19.1. **HRMS (ESI-Orbitrap, *m/z*):** $[M+H]^+$ Calcd for $C_{22}H_{22}N_3O_4^+$ 392.1605. Found 392.1619.

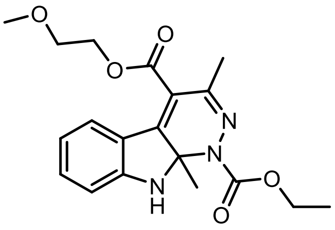

3i

1-Ethyl 4-(2-methoxyethyl) 3,9a-dimethyl-9,9a-dihydro-1*H*-pyridazino[3,4-*b*]indole-1,4-dicarboxylate (3i): compound **3i** was isolated by column chromatography (cyclohexane/ethyl acetate 8:2) in 68% yield (126 mg); red oil. **^{1}H NMR (400 MHz, DMSO-*d*$_6$)** δ 7.59 (d, *J* = 7.9 Hz, 1H), 7.39 (s, 1H), 7.31 (td, *J* = 8.3 Hz, *J* = 1.2 Hz, 1H), 6.89 (d, *J* = 8.3 Hz, 1H), 6.73 (td, *J* = 7.9 Hz, *J* = 0.9 Hz, 1H), 4.53–4.36 (m, 2H), 4.35–4.16 (m, 2H), 3.63 (t, *J* = 4.5 Hz, 2H), 3.28 (s, 3H), 2.13 (s, 3H), 1.28 (t, *J* = 7.1 Hz, 3H), 1.22 (s, 3H). **^{13}C{^{1}H} NMR (101 MHz, DMSO-*d*$_6$)** δ 165.5, 154.4, 152.8, 144.8, 143.6, 134.0, 125.8, 118.9, 118.2, 112.3, 111.6, 74.9, 69.5, 64.3, 62.0, 58.0, 19.6, 19.0, 14.4. **HRMS (ESI-Orbitrap, *m/z*):** $[M+H]^+$ Calcd for $C_{19}H_{24}N_3O_5^+$ 374.1710. Found 374.1716.

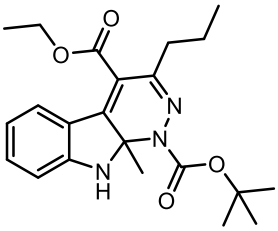

3j

1-(*tert*-Butyl) 4-ethyl 9a-methyl-3-propyl-9,9a-dihydro-1*H*-pyridazino[3,4-*b*]indole-1,4-dicarboxylate (3j): compound **3j** was isolated by column chromatography (cyclohexane/ethyl acetate 7:3) in 69% yield (137 mg); dark red oil. **^{1}H NMR (400 MHz, DMSO-*d*$_6$)** δ 7.48 (d, *J* = 7.9 Hz, 1H), 7.29 (td, *J* = 8.3 Hz, *J* = 1.2 Hz, 1H), 7.25 (s, 1H), 6.89 (d, *J* = 8.3 Hz, 1H), 6.73 (td, *J* = 7.9 Hz, *J* = 0.9 Hz, 1H), 4.55–4.13 (m, 2H), 2.60–2.53 (m, 1H), 2.36–2.29 (m, 1H), 1.62–1.40 (m, 2H), 1.51 (s, 9H), 1.29 (t, *J* = 7.1 Hz, 3H), 1.20 (s, 3H), 0.93 (t, *J* = 7.4 Hz, 3H). **^{13}C{^{1}H} NMR (101 MHz, DMSO-*d*$_6$)** δ 165.8, 153.4, 152.6, 146.7, 144.4, 133.7, 125.4, 118.8, 118.4, 112.3, 111.6, 81.4, 74.8, 61.4, 34.9, 27.9, 20.5, 18.7, 13.9, 13.6. **HRMS (ESI-Orbitrap, *m/z*):** $[M+H]^+$ Calcd for $C_{22}H_{30}N_3O_4^+$ 400.2231. Found 400.2223.

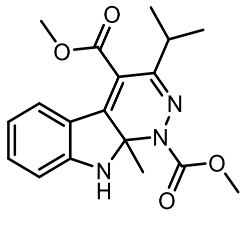

3k

1-Methyl 4-propyl 3,9a-dimethyl-9,9a-dihydro-1*H*-pyridazino[3,4-*b*]indole-1,4-dicarboxylate (3k): compound **3k** was isolated by column chromatography (cyclohexane/ethyl acetate 7:3) in 35% yield (60 mg); dark red oil. **^{1}H NMR (400 MHz, DMSO-*d*$_6$)** δ 7.40 (s, 1H), 7.38 (d, J = 7.9 Hz, 1H), 7.30 (td, J = 8.1 Hz, J = 1.1 Hz, 1H), 6.88 (d, J = 8.1 Hz, 1H), 6.74 (td, J = 7.9 Hz, J = 0.8 Hz, 1H), 3.88 (s, 3H), 3.80 (s, 3H), 2.82 (sept, J = 6.8 Hz, 1H), 1.19 (s, 3H), 1.16 (d, J = 6.8 Hz, 3H) 1.08 (d, J = 6.8 Hz, 3H). **^{13}C{^{1}H} NMR (101 MHz, DMSO-*d*$_6$)** δ 166.6, 155.1, 152.6, 151.6, 144.2, 133.8, 125.0, 119.0, 118.4, 112.2, 111.6, 75.0, 53.3, 52.6, 31.1, 22.3, 19.1, 18.9. **HRMS (ESI-Orbitrap, *m/z*):** [M+H]$^+$ Calcd for $C_{18}H_{22}N_3O_4^+$ 344.1605. Found 344.1610.

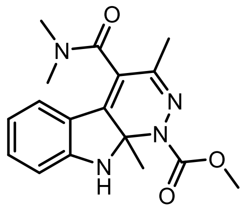

3l

Methyl 4-(dimethylcarbamoyl)-3,9a-dimethyl-9,9a-dihydro-1*H*-pyridazino[3,4-*b*]indole-1-carboxylate (3l): compound **3l** was isolated by column chromatography (cyclohexane/ethyl acetate 8:2) in 55% yield (90 mg); yellow solid; mp: 183–185 °C. **^{1}H NMR (400 MHz, DMSO)** δ 7.35–7.18 (m, 2H), 7.09 and 7.01 (d, J = 7.6 Hz, 1H), 6.89–6.83 (m, 1H), 6.76–6.69 (m, 1H), 3.79 and 3.78 (s, 3H), 3.11 and 3.00 (s, 3H), 3.09 and 2.60 (s, 3H), 2.00 and 1.99 (s, 3H), 1.27 and 1.21 (s, 3H). **^{13}C{^{1}H} NMR (101 MHz, DMSO-*d*$_6$)** δ 165.6 and 165.5, 154.8 and 154.7, 151.7 and 151.6, 143.4 and 143.1, 138.8 and 138.6, 132.9 and 132.8, 123.8 and 123.6, 119.2 and 119.1, 119.0 and 118.9, 115.6 and 115.4, 111.4 and 111.3, 74.5 and 74.1, 53.1 and 53.0, 37.1 and 36.4, 34.0 and 33.9, 19.6 and 19.4, 18.7 and 18.6. **HRMS (ESI-Orbitrap, *m/z*):** [M+H]$^+$ Calcd for $C_{17}H_{21}N_4O_3^+$ 329.1608. Found 329.1614.

Notably, compound **3l** at NMR analysis shows two sets of peaks indicating the presence of rotamers. This fact is probably ascribable to the presence of a second axis along the N−N bond that determines the

existence of syn/anti rotamers of carbamates.[4] This fact is probably ascribable to the presence of bulky substituents around C3-C2' bond that determines the existence of rotamers (ca 60:40 ratio);

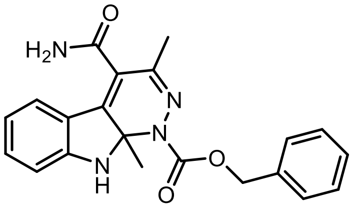

3m

Benzyl 4-carbamoyl-3,9a-dimethyl-9,9a-dihydro-1*H*-pyridazino[3,4-*b*]indole-1-carboxylate (3m): compound **3m** was isolated by column chromatography (cyclohexane/ethyl acetate 8:2) in 30% yield (56 mg); yellow solid; mp: 171–173 °C. **^{1}H NMR (400 MHz, DMSO-*d*$_6$)** δ 8.00 (s, 1H), 7.65 (s, 1H), 7.50 (d, J = 7.8 Hz, 1H), 7.46–7.32 (m, 5H), 7.23 (td, J = 8.0 Hz, J = 1.2 Hz, 1H), 7.17 (s, 1H), 6.85 (d, J = 8.0 Hz, 1H), 6.72 (td, J = 7.8 Hz, J = 0.8 Hz, 1H), 5.34 (d, J = 12.4 Hz, 1H), 5.22 (d, J = 12.4 Hz, 1H), 2.08 (s, 3H), 1.22 (s, 3H). **^{13}C{^{1}H} NMR (101 MHz, DMSO-*d*$_6$)** δ 167.1, 154.3, 151.6, 144.1, 139.2, 136.4, 132.6, 128.5, 128.1, 128.0, 124.8, 119.4, 118.7, 117.7, 111.3, 74.5, 67.0, 19.7, 19.0. **HRMS (ESI-Orbitrap, *m/z*):** $[M+H]^+$ Calcd for $C_{21}H_{21}N_4O_3^+$ 377.1608. Found 377.1599.

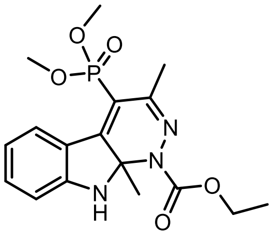

3n

Ethyl 4-(dimethoxyphosphoryl)-3,9a-dimethyl-9,9a-dihydro-1*H*-pyridazino[3,4-*b*]indole-1-carboxylate (3n): compound **3n** was isolated by column chromatography (cyclohexane/ethyl acetate 7:3) in 28% yield (53 mg); orange oil; **^{1}H NMR (400 MHz, DMSO-*d*$_6$)** δ 8.29 (d, J = 8.1 Hz, 1H), 7.44 (d, J = 1.9 Hz, 1H), 7.33 (t, J = 8.2 Hz, 1H), 6.91 (d, J = 8.1 Hz, 1H), 6.75 (t, J = 8.2 Hz, 1H), 4.51–3.95 (m, 2H), 3.64 (dd, J = 11.4, 9.3 Hz, 6H), 2.30 (d, J = 0.6 Hz, 3H), 1.28 (t, J = 7.1 Hz, 3H), 1.15 (s, 3H). **^{13}C{^{1}H} NMR (101 MHz, DMSO-*d*$_6$)** δ 156.8 (d, J_{CP}= 8.5 Hz), 154.8 (d, J_{CP}= 6.5 Hz), 146.3 (d, J_{CP}= 13.3 Hz), 134.7, 129.5, 118.5, 118.0 (d, J_{CP}= 5.4 Hz), 111.5, 105.0 (d, J_{CP}= 188.0 Hz), 75.7 (d, J_{CP}= 13.6

Hz), 62.1, 52.2 (d, J_{CP}= 5.4 Hz), 52.1, (d, J_{CP}= 5.3 Hz), 29.5, 22.1, 18.0, 14.3. **HRMS (ESI-Orbitrap, *m/z*):** $[M+H]^+$ Calcd for $C_{17}H_{23}N_3O_5P^+$ 380.1370. Found 380.1378.

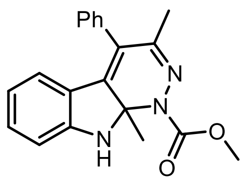

3o

Methyl 3,9a-dimethyl-4-phenyl-9,9a-dihydro-1*H*-pyridazino[3,4-*b*]indole-1-carboxylate (3o): compound **3o** was isolated by column chromatography (cyclohexane/ethyl acetate 8:2) in 20% yield (33.3 mg); dark yellow solid; mp: 164–166 °C. **^{1}H NMR (400 MHz, DMSO-*d*$_6$)** δ 7.60–7.41 (m, 4H), 7.14 (s, 1H), 7.13–7.07 (m, 2H), 6.80 (d, *J* = 8.0 Hz, 1H), 6.39 (td, *J* = 7.9 Hz, *J* = 0.9 Hz, 1H), 6.21 (d, *J* = 7.9 Hz, 1H), 3.80 (s, 3H), 1.83 (s, 3H), 1.32 (s, 3H). **^{13}C{^{1}H} NMR (101 MHz, DMSO-*d*$_6$)** δ 155.0, 151.6, 146.2, 139.4, 135.3, 131.8, 130.7, 129.2, 129.0, 128.2, 127.8, 123.9, 121.0, 120.5, 118.1, 111.0, 74.7, 53.0, 20.1, 20.0. **HRMS (ESI-Orbitrap, *m/z*):** $[M+H]^+$ Calcd for $C_{20}H_{20}N_3O_2^+$ 334.1550. Found 334.1553.

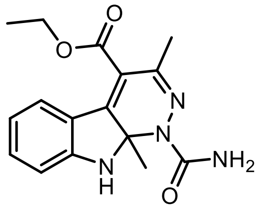

3p

Ethyl 1-carbamoyl-3,9a-dimethyl-9,9a-dihydro-1H-pyridazino[3,4-b]indole-4-carboxylate (3p): compound **3p** was isolated by column chromatography (cyclohexane/ethyl acetate 6.5:3.5) in 54% yield (84 mg); red oil. **^{1}H NMR (400 MHz, DMSO-*d*$_6$)** δ 7.47 (d, *J* = 7.8 Hz, 1H), 7.36 (s, 1H), 7.26 (td, *J* = 8.3 Hz, *J* = 1.2 Hz, 1H), 6.89 (d, *J* = 8.3 Hz, 1H), 6.82 (s, 2H), 6.69 (td, *J* = 7.8 Hz, *J* = 1.0 Hz, 1H), 4.52–4.19 (m, 2H), 2.13 (s, 3H), 1.30 (t, *J* = 7.1 Hz, 3H), 1.17 (s, 3H). **^{13}C{^{1}H} NMR (101 MHz, DMSO-*d*$_6$)** δ 165.8, 157.7, 152.4, 144.2, 140.5, 133.7, 125.4, 118.3, 117.9, 112.6, 111.3, 74.4, 61.4, 19.7, 19.3, 13.9. **HRMS (ESI-Orbitrap, *m/z*):** $[M+H]^+$ Calcd for $C_{16}H_{19}N_4O_3^+$ 315.1452. Found 315.1441.

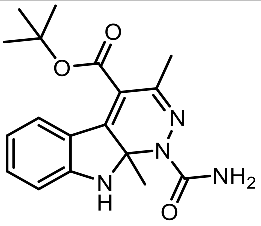

3q

***tert*-Butyl 1-carbamoyl-3,9a-dimethyl-9,9a-dihydro-1*H*-pyridazino[3,4-*b*]indole-4-carboxylate (3q):** compound **3q** was isolated by column chromatography (cyclohexane/ethyl acetate 6:4) in 75% yield (128.4 mg); dark yellow solid; mp: 155–157 °C; **^{1}H NMR (400 MHz, DMSO-*d*$_6$)** δ 7.51 (d, *J* = 7.9 Hz, 1H), 7.30 (s, 1H), 7.25 (td, *J* = 8.3 Hz, *J* = 1.2 Hz, 1H), 6.88 (d, *J* = 8.3 Hz, 1H), 6.81 (s, 2H), 6.69 (td, *J* = 7.9 Hz, *J* = 0.9 Hz, 1H), 2.14 (s, 3H), 1.54 (s, 9H), 1.16 (s, 3H). **^{13}C{^{1}H} NMR (101 MHz, DMSO-*d*$_6$)** δ 165.2, 157.7, 152.1, 142.9, 140.5, 133.5, 125.1, 118.3, 118.2, 114.2, 111.3, 82.5, 74.3, 27.7, 19.7, 19.4. **HRMS (ESI-Orbitrap, *m/z*):** [M+H]$^+$ Calcd for $C_{18}H_{23}N_4O_3^+$ 343.1765. Found 343.1757.

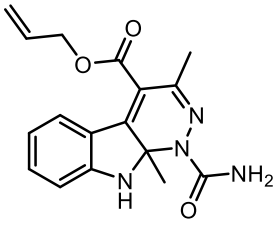

3r

Allyl 1-carbamoyl-3,9a-dimethyl-9,9a-dihydro-1*H*-pyridazino[3,4-*b*]indole-4-carboxylate (3r): compound **3r** was isolated by column chromatography (cyclohexane/ethyl acetate 6:4) in 60% yield (97 mg); red oil. **^{1}H NMR (400 MHz, DMSO-*d*$_6$)** δ 7.48 (d, *J* = 8.0 Hz, 1H), 7.40 (s, 1H), 7.27 (td, *J* = 8.3 Hz, *J* = 1.2 Hz, 1H), 6.89 (d, *J* = 8.3 Hz, 1H), 6.84 (s, 2H), 6.67 (td, *J* = 8.0 Hz, *J* = 1, 1H), 6.10–5.98 (m, 1H), 5.44–5.39 (m, 1H), 5.33–5.29 (m, 1H), 4.85–4.83 (m, 2H), 2.13 (s, 3H), 1.17 (s, 3H). **^{13}C{^{1}H} NMR (101 MHz, DMSO-*d*$_6$)** δ 165.5, 157.7, 152.5, 144.6, 140.4, 133.8, 131.9, 125.5, 119.5, 118.3, 117.8, 112.2, 111.4, 74.4, 65.9, 19.7, 19.3. **HRMS (ESI-Orbitrap, *m/z*):** [M+H]$^+$ Calcd for $C_{17}H_{19}N_4O_3^+$ 327.1452. Found 327.1458.

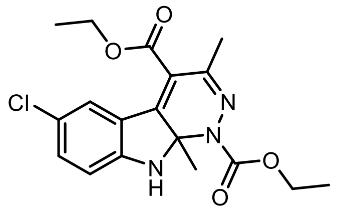

3s

Diethyl 6-chloro-3,9a-dimethyl-9,9a-dihydro-1*H*-pyridazino[3,4-*b*]indole-1,4-dicarboxylate (3s): compound **3s** was isolated by column chromatography (cyclohexane/ethyl acetate 7:3) in 55% yield (103 mg); orange solid; mp: 117–119 °C. **^{1}H NMR (400 MHz, DMSO-*d*$_6$)** δ 7.62 (s, 1H), 7.54 (d, *J* = 2.2 Hz, 1H), 7.35 (dd, *J* = 8.6, *J* = 2.2 Hz, 1H), 6.91 (d, *J* = 8.6 Hz, 1H), 4.44–4.16 (m, 4H), 2.15 (s, 3H), 1.32 (t, *J* = 7.2 Hz, 3H), 1.28 (t, *J* = 7.2 Hz, 3H), 1.22 (s, 3H). **^{13}C{^{1}H} NMR (101 MHz, DMSO-*d*$_6$)** δ 165.0, 154.3, 151.6, 143.7, 143.6, 133.7, 124.9, 122.1, 119.7, 113.5, 113.1, 75.5, 62.2, 61.7, 19.9, 18.9, 14.4, 13.8. **HRMS (ESI-Orbitrap, *m/z*):** $[M+H]^+$ Calcd for $C_{18}H_{21}ClN_3O_4^+$ 378.1215. Found 378.1212.

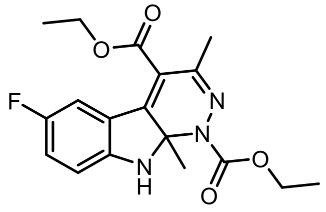

3t

Diethyl 6-fluoro-3,9a-dimethyl-9,9a-dihydro-1*H*-pyridazino[3,4-*b*]indole-1,4-dicarboxylate (3t): compound **3t** was isolated by column chromatography (cyclohexane/ethyl acetate 7:3) in 60% yield (108 mg); orange solid; mp: 105–107 °C. **^{1}H NMR (400 MHz, DMSO-*d*$_6$)** δ 7.36 (s, 1H), 7.29 (dd, *J* = 9.4, *J* = 2.7 Hz, 1H), 7.21 (td, *J* = 9.0, *J* = 2.7 Hz, 1H), 6.91 (dd, *J* = 8.9, *J* = 4.6 Hz, 1H), 4.49–4.09 (m, 4H), 2.14 (s, 3H), 1.30 (t, *J* = 7.1 Hz, 3H), 1.29 (t, *J* = 7.1 Hz, 3H), 1.22 (s, 3H). **^{13}C{^{1}H} NMR (101 MHz, DMSO)** δ 165.1, 155.4 (d, J_{CF} = 233.6 Hz), 154.3, 149.5, 144.4 (d, J_{CF} = 3.6 Hz), 143.4, 121.3 (d, J_{CF} = 24.4 Hz), 118.7 (d, J_{CF} = 9.3 Hz), 113.4, 112.7 (d, J_{CF} = 8.2 Hz), 111.1 (d, J_{CF} = 25.1 Hz), 75.6, 62.1, 61.6, 19.8, 18.9, 14.3, 13.8. **^{19}F{^{1}H} NMR (376 MHz, DMSO-*d*$_6$)** δ -124.85. **HRMS (ESI-Orbitrap, *m/z*):** $[M+H]^+$ Calcd for $C_{18}H_{21}FN_3O_4^+$ 362.1511. Found 362.1503.

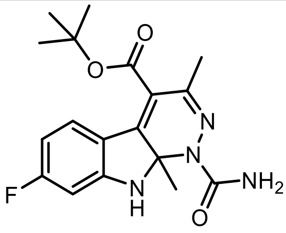

3u

***tert*-Butyl 1-carbamoyl-7-fluoro-3,9a-dimethyl-9,9a-dihydro-1*H*-pyridazino[3,4-*b*]indole-4-carboxylate (3u):** compound **3u** was isolated by column chromatography (cyclohexane/ethyl acetate 6:4) in 47% yield (83 mg); orange solid; mp: 111–113 °C. **^{1}H NMR (400 MHz, DMSO-*d*$_6$)** δ 7.66 (s, 1H), 7.56 (dd, J = 8.6, J = 5.9 Hz, 1H), 6.84 (s, 2H), 6.64 (dd, J = 10.3, J = 2.3 Hz, 1H), 6.51 (td, J = 9.3, J = 2.3 Hz, 1H), 2.14 (s, 3H), 1.53 (s, 9H), 1.16 (s, 3H). **^{13}C{^{1}H} NMR (101 MHz, DMSO)** δ 166.0 (d, J_{CF} = 247.7 Hz), 165.0, 157.7, 154.1 (d, J_{CF} = 13.8 Hz), 142.2, 140.9, 127.2 (d, J_{CF} = 11.4 Hz), 114.8 (d, J_{CF} = 1.1 Hz), 113.8 (d, J_{CF} = 2.1 Hz), 105.7 (d, J_{CF} = 24.1 Hz), 97.6 (d, J_{CF} = 26.4 Hz), 82.6, 75.1, 27.7, 19.8, 19.2. **^{19}F{^{1}H} NMR (376 MHz, DMSO-*d*$_6$)** δ -105.44. **HRMS (ESI-Orbitrap, *m/z*):** [M+H]$^+$ Calcd for $C_{18}H_{22}FN_4O_3^+$ 361.1670. Found 361.1662.

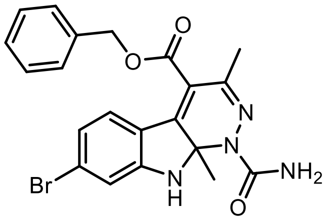

3v

Benzyl 7-bromo-1-carbamoyl-3,9a-dimethyl-9,9a-dihydro-1*H*-pyridazino[3,4-*b*]indole-4-carboxylate (3v): compound **3v** was isolated by column chromatography (cyclohexane/ethyl acetate 7:3) in 40% yield (83 mg); red oil. **^{1}H NMR (400 MHz, DMSO-*d*$_6$)** δ 7.70 (s, 1H), 7.49–7.35 (m, 5H), 7.29 (d, J = 8.4 Hz, 1H), 7.06 (d, J = 1.8 Hz, 1H), 6.86 (bs, 2H), 6.72 (dd, J = 8.4, J = 1.8 Hz, 1H), 5.40 (d, J = 12.0 Hz, 1H), 5.35 (d, J = 12.0 Hz, 1H), 2.09 (s, 3H), 1.15 (s, 3H). **^{13}C{^{1}H} NMR (101 MHz, DMSO)** δ 165.3, 157.5, 153.3, 143.1, 140.4, 135.1, 129.1, 128.6 (2C), 127.1, 127.0, 120.8, 117.1, 113.6, 112.8, 74.8, 67.3, 19.7, 19.2. **HRMS (ESI-Orbitrap, *m/z*):** [M+H]$^+$ Calcd for $C_{21}H_{20}BrN_4O_3^+$ 455.0713. Found 455.0719.

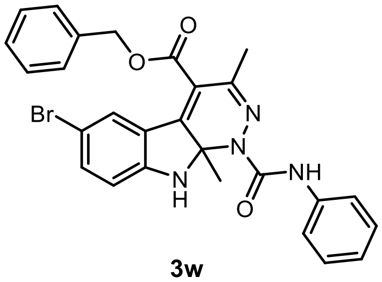

3w

Benzyl 6-bromo-3,9a-dimethyl-1-(phenylcarbamoyl)-9,9a-dihydro-1*H*-pyridazino[3,4-*b*]indole-4-carboxylate (3w): compound **3w** was isolated by column chromatography (cyclohexane/ethyl acetate 7:3) in 51% yield (135 mg); dark yellow solid; mp: 151–153 °C. **^{1}H NMR (400 MHz, DMSO-*d*$_6$)** δ 9.36 (s, 1H), 7.79 (s, 1H), 7.64–7.37 (m, 9H), 7.30 (t, J = 7.9 Hz, 2H), 7.03 (t, J = 7.4 Hz, 1H), 6.87 (d, J = 8.6 Hz, 1H), 5.42 (d, J = 11.8 Hz, 1H), 5.37 (d, J = 11.8 Hz, 1H), 2.21 (s, 3H), 1.25 (s, 3H). **^{13}C{^{1}H} NMR (101 MHz, DMSO-*d*$_6$)** δ 165.1, 153.8, 151.9, 143.8, 142.1, 138.7, 136.4, 135.0, 129.1, 128.7, 128.6, 128.5, 127.9, 122.9, 119.9, 119.5, 113.4, 113.2, 109.1, 75.2, 67.5, 19.9, 18.8. **HRMS (ESI-Orbitrap, *m/z*):** [M+H]$^+$ Calcd for $C_{27}H_{24}BrN_4O_3^+$ 531.1026. Found 531.1027.

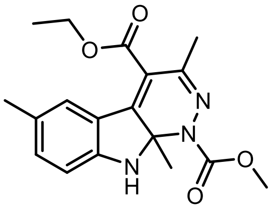

3x

4-Ethyl 1-methyl 3,6,9a-trimethyl-9,9a-dihydro-1*H*-pyridazino[3,4-*b*]indole-1,4-dicarboxylate (3x): compound **3x** was isolated by column chromatography (cyclohexane/ethyl acetate 7:3) in 68% yield (116.7 mg); orange solid; mp: 117–119 °C. **^{1}H NMR (400 MHz, DMSO-*d*$_6$)** δ 7.31 (s, 1H), 7.23 (s, 1H), 7.18–7.11 (m, 1H), 6.81 (d, J = 8.2 Hz, 1H), 4.46–4.24 (m, 2H), 3.79 (s, 3H), 2.20 (s, 3H), 2.11 (s, 3H), 1.32 (t, J = 7.1 Hz, 3H), 1.20 (s, 3H). **^{13}C{^{1}H} NMR (101 MHz, DMSO-*d*$_6$)** δ 165.6, 154.9, 150.9, 144.7, 143.5, 135.1, 127.7, 125.3, 118.4, 112.3, 111.7, 74.9, 61.5, 53.2, 20.5, 19.6, 19.2, 13.9. **HRMS (ESI-Orbitrap, *m/z*):** [M+H]$^+$ Calcd for $C_{18}H_{22}N_3O_4^+$ 344.1605. Found 344.1600.

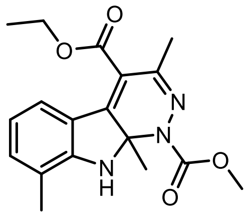

3y

4-Ethyl 1-methyl 3,8,9a-trimethyl-9,9a-dihydro-1H-pyridazino[3,4-b]indole-1,4-dicarboxylate (3y): compound **3y** was isolated by column chromatography (cyclohexane/ethyl acetate 7:3) in 60% yield (100 mg); orange solid; mp: 141–143 °C. **^{1}H NMR (400 MHz, DMSO-*d*$_6$)** δ 7.36 (d, *J* = 7.9 Hz, 1H), 7.16 (d, *J* = 7.2 Hz, 1H), 6.73 (t, *J* = 7.6 Hz, 1H), 6.63 (s, 1H), 4.41–4.30 (m, 2H), 3.80 (s, 3H), 2.20 (s, 3H), 2.11 (s, 3H), 1.30 (t, *J* = 7.1 Hz, 3H), 1.25 (s, 3H). **^{13}C{^{1}H} NMR (101 MHz, DMSO-*d*$_6$)** δ 165.6, 155.0, 151.2, 144.6, 143.4, 134.5, 122.8, 121.2, 119.7, 118.2, 112.6, 75.0, 61.5, 53.2, 19.6, 19.4, 16.4, 13.9. **HRMS (ESI-Orbitrap, *m/z*):** [M+H]$^+$ Calcd for $C_{18}H_{22}N_3O_4^+$ 344.1605. Found 344.1603.

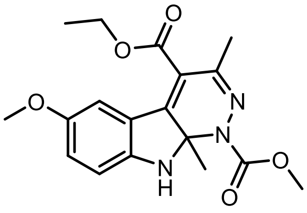

3z

4-Ethyl 1-methyl 6-methoxy-3,9a-dimethyl-9,9a-dihydro-1*H*-pyridazino[3,4-*b*]indole-1,4-dicarboxylate (3z): compound **3z** was isolated by column chromatography (cyclohexane/ethyl acetate 7:3) in 56% yield (100 mg); orange solid; mp: 149–151 °C. **^{1}H NMR (400 MHz, DMSO-*d*$_6$)** δ 7.09 (s, 1H), 7.05–7.00 (m, 2H), 6.86 (d, *J* = 8.6 Hz, 1H), 4.37 (q, *J* = 7.1 Hz, 2H), 3.78 (s, 3H), 3.68 (s, 3H), 2.12 (s, 3H), 1.32 (t, *J* = 7.1 Hz, 3H), 1.21 (s, 3H). **^{13}C{^{1}H} NMR (101 MHz, DMSO-*d*$_6$)** δ 165.6, 154.9, 152.6, 147.7, 145.1, 143.3, 122.5, 118.6, 112.9, 112.4, 108.2, 75.1, 61.6, 55.4, 53.2, 19.7, 19.2, 13.9. **HRMS (ESI-Orbitrap, *m/z*):** [M+H]$^+$ Calcd for $C_{18}H_{22}N_3O_5^+$ 360.1554. Found 360.1541.

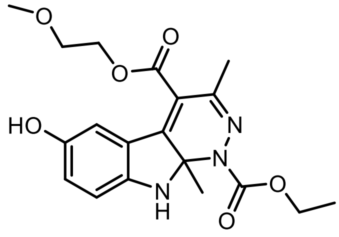

3za

1-Ethyl 4-(2-methoxyethyl) 6-hydroxy-3,9a-dimethyl-9,9a-dihydro-1*H*-pyridazino[3,4-*b*]indole-1,4-dicarboxylate (3za): compound **3za** was isolated by column chromatography (cyclohexane/ethyl acetate 6:4) in 62% yield (120 mg); dark red oil. **^{1}H NMR (400 MHz, DMSO-*d*$_6$)** δ 8.97 (s, 1H), 6.96 (d, *J* = 2.4 Hz, 1H), 6.84–6.75 (m, 3H), 4.50–4.35 (m, 2H), 4.34–4.14 (m, 2H), 3.69–3.59 (m, 2H), 3.27 (s, 3H), 2.11 (s, 3H), 1.27 (t, *J* = 7.1 Hz, 3H), 1.20 (s, 3H). **^{13}C{^{1}H} NMR (101 MHz, DMSO-*d*$_6$)** δ 165.6, 154.4, 150.4, 146.5, 145.4, 143.2, 122.9, 118.8, 112.7, 112.1, 110.3, 74.9, 69.5, 64.1, 61.9, 58.0, 19.6, 19.2, 14.4. **HRMS (ESI-Orbitrap, *m/z*):** [M+H]$^+$ Calcd for $C_{19}H_{24}N_3O_6^+$ 390.1660. Found 390.1667.

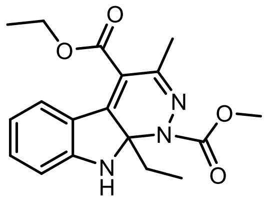

3zb

4-Ethyl 1-methyl 9a-ethyl-3-methyl-9,9a-dihydro-1*H*-pyridazino[3,4-*b*]indole-1,4-dicarboxylate (3zb): compound **3zb** was isolated by column chromatography (cyclohexane/ethyl acetate 7:3) in 60% yield (103 mg); red solid; mp: 106–108 °C. **^{1}H NMR (400 MHz, DMSO-*d*$_6$)** δ 7.49 (s, 1H), 7.44 (d, *J* = 7.9 Hz, 1H), 7.28 (td, *J* = 8.3 Hz, *J* = 1.2 Hz, 1H), 6.85 (d, *J* = 8.3 Hz, 1H), 6.70 (td, *J* = 7.9 Hz, *J* = 0.9 Hz, 1H), 4.41–4.29 (m, 2H), 3.78 (s, 3H), 2.10 (s, 3H), 1.86–1.60 (m, 2H), 1.30 (t, *J* = 7.1 Hz, 3H), 0.62 (t, *J* = 7.5 Hz, 3H). **^{13}C{^{1}H} NMR (101 MHz, DMSO-*d*$_6$)** δ 165.6, 154.6, 153.3, 143.5, 143.0, 133.9, 124.7, 119.5, 118.6, 113.0, 110.7, 77.9, 61.5, 53.1, 27.3, 19.5, 13.9, 7.2. **HRMS (ESI-Orbitrap, *m/z*):** [M+H]$^+$ Calcd for $C_{18}H_{22}N_3O_4^+$ 344.1605. Found 344.1603.

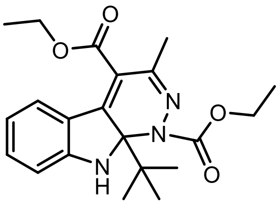

3zc

Diethyl 9a-(*tert*-butyl)-3-methyl-9,9a-dihydro-1*H*-pyridazino[3,4-*b*]indole-1,4-dicarboxylate (3zc): compound **3zc** was isolated by column chromatography (cyclohexane/ethyl acetate 7:3) in 60% yield (115 mg); orange solid; mp: 164–166 °C. **^{1}H NMR (400 MHz, DMSO-*d*$_6$)** δ 7.66 (s, 1H), 7.34 (d, *J* = 7.8 Hz, 1H), 7.22 (t, *J* = 8.1 Hz, 1H), 6.85 (d, *J* = 8.1 Hz, 1H), 6.62 (t, *J* = 7.8 Hz, 1H), 4.41–4.28 (m, 2H), 4.26–4.06 (m, 2H), 2.07 (s, 3H), 1.30 (t, *J* = 7.1 Hz, 3H), 1.25 (t, *J* = 7.1 Hz, 3H), 0.77 (s, 9H). **^{13}C{^{1}H} NMR (101 MHz, DMSO-*d*$_6$)** δ 165.8, 153.3, 152.4, 144.0, 139.4, 133.4, 123.7, 120.5, 117.8, 114.9, 109.5, 82.4, 61.8, 61.5, 44.0, 24.4, 19.1, 14.4, 13.9. **HRMS (ESI-Orbitrap, *m/z*):** [M+H]$^+$ Calcd for $C_{21}H_{28}N_3O_4^+$ 386.2074. Found 386.2072.

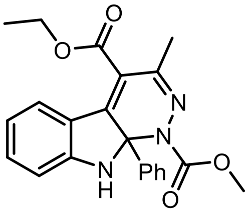

3zd

4-Ethyl 1-methyl 3-methyl-9a-phenyl-9,9a-dihydro-1*H*-pyridazino[3,4-*b*]indole-1,4-dicarboxylate (3zd): compound **3zd** was isolated by column chromatography (cyclohexane/ethyl acetate 7:3) in 65% yield (127 mg); orange solid; mp: 156–158 °C. **^{1}H NMR (400 MHz, DMSO-*d*$_6$)** δ 8.13 (s, 1H), 7.44 (d, *J* = 7.8 Hz, 1H), 7.36 (dt, *J* = 8.2 Hz, *J* = 1.0 Hz, 1H), 7.31–7.27 (m, 3H), 7.14–7.08 (m, 2H), 6.99 (d, *J* = 8.2 Hz, 1H), 6.77 (t, *J* = 7.8 Hz, 1H), 4.42–4.25 (m, 2H), 3.79 (s, 3H), 2.11 (s, 3H), 1.28 (t, *J* = 7.1 Hz, 3H). **^{13}C{^{1}H} NMR (101 MHz, DMSO-*d*$_6$)** δ 165.5, 154.7, 152.8, 143.5, 142.4, 139.5, 134.2, 128.8, 128.7, 125.2, 125.1, 119.2, 118.9, 113.2, 110.8, 78.2, 61.7, 53.4, 19.5, 13.9. **HRMS (ESI-Orbitrap, *m/z*):** [M+H]$^+$ Calcd for $C_{22}H_{22}N_3O_4^+$ 392.1605. Found 392.1598.

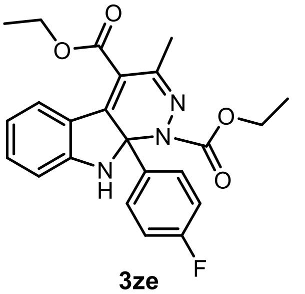

3ze

Diethyl 9a-(4-fluorophenyl)-3-methyl-9,9a-dihydro-1*H*-pyridazino[3,4-*b*]indole-1,4-dicarboxylate (3ze): compound **3ze** was isolated by column chromatography (cyclohexane/ethyl acetate 8:2) in 54% yield (114 mg); red oil. **^{1}H NMR (400 MHz, DMSO-*d*$_6$)** δ 8.15 (s, 1H), 7.45 (d, *J* = 7.8 Hz, 1H), 7.36 (td, *J* = 8.3 Hz, *J* = 1.2 Hz, 1H), 7.17–7.14 (m, 4H), 6.99 (d, *J* = 8.3 Hz, 1H), 6.77 (td, *J* = 7.8 Hz, *J* = 0.8 Hz, 1H), 4.39–4.16 (m, 4H), 2.12 (s, 3H), 1.28 (t, *J* = 6.6 Hz, 3H), 1.25 (t, *J* = 6.6 Hz, 3H). **^{13}C{^{1}H} NMR (101 MHz, DMSO-*d*$_6$)** δ 165.4, 162.1 (d, J_{CF} = 245.4 Hz), 154.2, 152.6, 143.3, 142.4, 135.8 (d, J_{CF} = 3.0 Hz), 134.3, 127.4 (d, J_{CF} = 8.4 Hz), 125.3, 119.3, 118.8, 115.6 (d, J_{CF} = 21.6 Hz), 113.4, 110.9, 77.7, 62.4, 61.7, 19.5, 14.3, 13.9. **^{19}F{^{1}H} NMR (376 MHz, DMSO-*d*$_6$)** δ -113.31. **HRMS (ESI-Orbitrap, *m/z*):** $[M+H]^+$ Calcd for $C_{23}H_{23}FN_3O_4^+$ 424.1667. Found 424.1661.

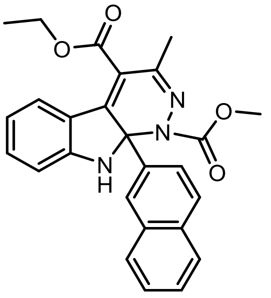

3zf

4-Ethyl 1-methyl 3-methyl-9a-(naphthalen-2-yl)-9,9a-dihydro-1*H*-pyridazino[3,4-*b*]indole-1,4-dicarboxylate (3zf): compound **3zf** was isolated by column chromatography (cyclohexane/ethyl acetate 7.5:2.5) in 37% yield (80 mg); dark orange oil. **^{1}H NMR (400 MHz, DMSO-*d*$_6$)** δ 8.26 (s, 1H), 7.87–7.81 (m, 3H), 7.57 (d, *J* = 2.0 Hz, 1H), 7.52–7.44 (m, 3H), 7.39 (td, *J* = 8.3 Hz, *J* = 1.2 Hz, 1H), 7.25 (dd, *J* = 8.7, *J* = 2.0 Hz, 1H), 7.04 (d, *J* = 8.3 Hz, 1H), 6.81 (td, *J* = 7.8 Hz, *J* = 0.9 Hz, 1H), 4.40–4.27 (m, 2H), 3.80 (s, 3H), 2.13 (s, 3H), 1.28 (t, *J* = 7.1 Hz, 3H). **^{13}C{^{1}H} NMR (101 MHz, DMSO-*d*$_6$)** δ 165.4, 154.7, 152.8, 143.3, 142.5, 136.7, 134.3, 132.8, 132.2, 128.8, 128.3, 127.3, 126.7, 126.6, 125.2, 124.1, 123.0, 119.3, 119.0, 113.4, 110.9, 78.2, 61.7, 53.5, 19.5, 13.9. **HRMS (ESI-Orbitrap, *m/z*):** $[M+H]^+$ Calcd for $C_{26}H_{24}N_3O_4^+$ 442.1761. Found 442.1756.

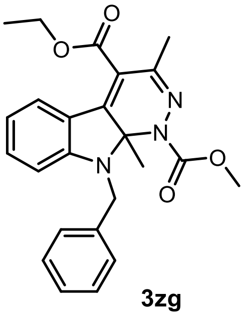

3zg

4-Ethyl 1-methyl 9-benzyl-3,9a-dimethyl-9,9a-dihydro-1*H*-pyridazino[3,4-*b*]indole-1,4-dicarboxylate (3zg): compound **3zg** was isolated by column chromatography (cyclohexane/ethyl acetate 8:2) in 37% yield (77.6 mg); orange oil. **^{1}H NMR (400 MHz, DMSO-*d*$_6$)** δ 7.49 (dd, *J* = 7.9 Hz, *J* = 0.7 Hz, 1H), 7.39–7.13 (m, 6H), 6.85 (td, *J* = 7.9 Hz, *J* = 0.7 Hz, 1H), 6.26 (d, *J* = 8.2 Hz, 1H), 5.54 (d, *J* = 16.1 Hz, 1H), 4.40 (q, *J* = 7.1 Hz, 2H), 4.07 (d, *J* = 16.1 Hz, 1H), 3.55 (s, 3H), 2.16 (s, 3H), 1.35 (s, 3H), 1.32 (t, *J* = 7.1 Hz, 3H). **^{13}C{^{1}H} NMR (101 MHz, DMSO-*d*$_6$)** δ 165.6, 155.3, 154.7, 144.5, 142.6, 139.5, 133.8, 128.3, 126.3, 126.2, 124.4, 120.3, 119.4, 113.0, 111.5, 80.4, 61.8, 53.2, 53.0, 19.2, 16.1, 13.9. **HRMS (ESI-Orbitrap, *m/z*):** $[M+H]^+$ Calcd for $C_{24}H_{26}N_3O_4^+$ 420.1918; Found 420.1915.

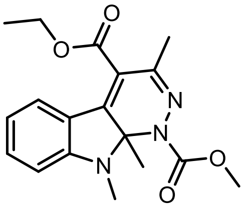

3zh

4-Ethyl 1-methyl 3,9,9a-trimethyl-9,9a-dihydro-1*H*-pyridazino[3,4-*b*]indole-1,4-dicarboxylate (3zh): compound **3zh** was isolated by column chromatography (cyclohexane/ethyl acetate 7.5:2.5) in 60% yield (103 mg); red solid; mp: 110–112 °C. **^{1}H NMR (400 MHz, DMSO-*d*$_6$)** δ 7.48 (d, *J* = 7.8 Hz, 1H), 7.41 (t, *J* = 8.2 Hz, 1H), 6.89 (d, *J* = 8.2 Hz, 1H), 6.83 (t, *J* = 7.8 Hz, 1H), 4.42–4.32 (m, 2H), 3.72 (s, 3H), 3.11 (s, 3H), 2.14 (s, 3H), 1.31 (t, *J* = 7.1 Hz, 3H), 1.24 (s, 3H). **^{13}C{^{1}H} NMR (101 MHz, DMSO-*d*$_6$)** δ 165.6, 156.3, 153.7, 145.9, 143.4, 134.2, 124.8, 119.8, 119.3, 112.4, 111.1, 80.0, 61.6, 53.1, 34.9, 19.4, 15.5, 13.8. **HRMS (ESI-Orbitrap, *m/z*):** $[M+H]^+$ Calcd for $C_{18}H_{22}N_3O_4^+$ 344.1605; Found 344.1597.

5. Preparation method for crystal growth of compound 3zh. About 30 mg of compound **3zh** was added to a 25 ml flask, then diethyl ether (about 10 mL) solvent was added. After 12 h, the crystal crystallized.

6. Procedure for scale-up reaction.

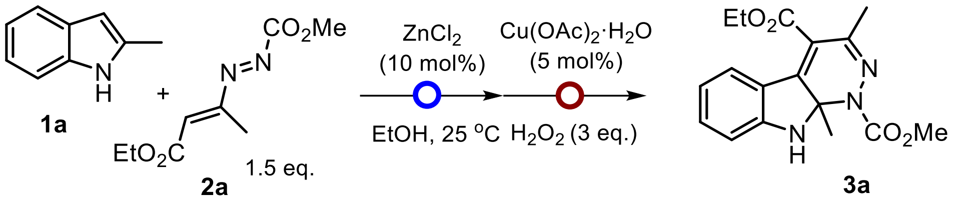

To a stirred mixture of indole **1a** (1.05 g, 8.0 mmol) and azoalkene **2a** (2.40 g, 12 mmol, 1.5 eq.) in ethanol (30 mL), $ZnCl_2$ (109.0 mg, 0.8 mmol, 10 mol%) was added. Upon disappearance of **1a** (12 h, TLC check), to the same reaction mixture $Cu(OAc)_2{\cdot}H_2O$ (79.9 mg, 0.4 mmol, 5 mol%) and H_2O_2 (40 wt%, aqueous, 1.9 mL, 3 eq.) were sequentially added. After TLC showed complete consumption of intermediate **A** (TLC check), the solvent was removed, and the crude mixture was purified by column chromatography on silica gel to afford the product **3a** (1.56 g, 59% yield).

7. Derivatization of 3a,d.

Access to 4a.

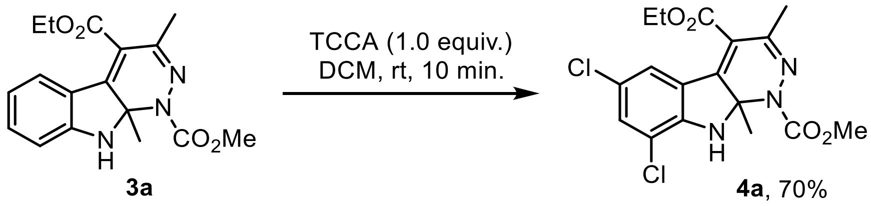

Pyridazinoindoline **3a** (66 mg, 0.2 mmol) was dissolved in dichloromethane (3 mL), and trichloroisocyanuric acid (TCCA, 47 mg, 0.2 mmol, 1.0 equiv) was added. The reaction was stirred at room temperature and monitored by TLC. Upon completion (10 min.), the mixture was directly purified by flash column chromatography on silica gel (cyclohexane/ethyl acetate 8:2) to yield compound **4a** (56 mg, 70% yield).

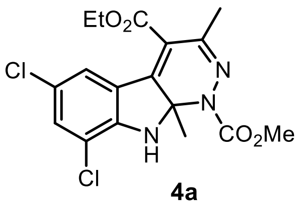

4a

4-Ethyl 1-methyl 6,8-dichloro-3,9a-dimethyl-9,9a-dihydro-1*H*-pyridazino[3,4-*b*]indole-1,4-dicarboxylate (4a): compound **4a** was isolated by column chromatography (cyclohexane/ethyl acetate 8:2) in 70% yield (56 mg); orange solid; mp: 144–146 °C. **^{1}H NMR (400 MHz, DMSO-*d*$_6$)** δ 7.63 (d, *J* = 1.8 Hz, 1H), 7.53 (d, *J* = 1.8 Hz, 1H), 6.97 (s, 1H), 4.45–4.32 (m, 2H), 3.81 (s, 3H), 2.16 (s, 3H), 1.31 (t, *J* = 7.1 Hz, 3H), 1.30 (s, 3H). **^{13}C{^{1}H} NMR (101 MHz, DMSO-*d*$_6$)** δ 164.6, 154.6, 147.5, 143.5, 141.8, 132.4, 123.9, 123.0, 121.6, 116.6, 115.0, 75.8, 62.0, 53.4, 19.7, 19.1, 13.8. **HRMS (ESI-Orbitrap, *m/z*):** [M+H]$^+$ Calcd for $C_{17}H_{18}Cl_2N_3O_4^+$ 398.0669; Found 398.0677.

Access to 5a.

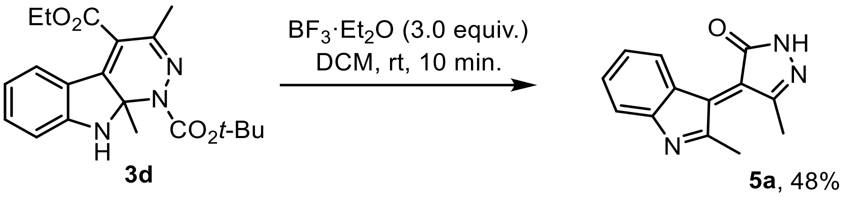

Pyridazinoindoline **3d** (74 mg, 0.2 mmol) was dissolved in dichloromethane (2 mL), and boron trifluoride diethyl etherate ($BF_3{\cdot}Et_2O$, 74 µL, 0.6 mmol, 3.0 equiv) was added. The reaction was stirred at room temperature and monitored by TLC. Upon completion, the mixture was quenched with saturated aqueous $NaHCO_3$, extracted, dried over anhydrous Na_2SO_4, and concentrated under reduced pressure. The residue was purified by flash column chromatography on silica gel (ethyl acetate/methanol 95:5) to yield compound **5a** (22 mg, 48% yield).

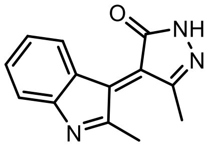

5a

5-Methyl-4-(2-methyl-3*H*-indol-3-ylidene)-2,4-dihydro-3*H*-pyrazol-3-one (5a): compound **5a** was isolated by column chromatography (ethyl acetate/methanol 9.9:0.1) in 48% yield (22 mg); white solid; mp: > 200 °C (dec.). **^{1}H NMR (400 MHz, DMSO-*d*$_6$)** δ 12.20 (s, 1H), 8.49 (d, *J* = 8.4 Hz, 1H), 7.68 (td, *J* = 7.6 Hz, *J* = 0.8 Hz, 1H), 7.44 (d, *J* = 7.6 Hz, 1H), 7.37 (td, *J* = 8.4 Hz, *J* = 1.1 Hz, 1H), 3.19 (s, 3H), 3.06 (s, 3H). **^{13}C{^{1}H} NMR (101 MHz, DMSO-*d*$_6$)** δ 159.9, 157.0, 154.9, 139.6, 131.8, 131.3, 127.9, 122.4, 120.1, 116.1, 114.5, 26.3, 23.8. **HRMS (ESI-Orbitrap, *m/z*):** [M+H]$^+$ Calcd for $C_{13}H_{12}N_3O^+$ 226.0975; Found 226.0963.

8. X-ray structure of compound 3zh (CCDC: 2467779)

Single-crystal diffraction data for compound **3zh** have been collected on a SuperNova dual source diffractometer with an Atlas detector at 150 K using a mirror monochromator and MoKα radiation with λ = 0,71073 Å. The diffraction data were processed using CrysAlis Pro software.[5] Structure was solved by direct methods, using Sir2014.[6] Full-matrix least-squares refinements on F[6] were done with anisotropic displacement parameters for all non-hydrogen atoms. Positions of all H atoms were seen in difference Fourier map. Aromatic and methylene H atoms were finally placed at calculated positions and treated as riding model. H atoms from methyl groups were also located using riding model, only torsion angle of idealized methyl group was refined in all cases according to electron density. Shelxl-2018/3 software[7] was used for structure refinement and interpretation. Drawing of the structure (Figure 1) was produced using Ortep-III.[8] Structure is monoclinic, with $P2_1/c$ space group and a = 14.9413(10), b = 13.7577(11), c = 7.9910(5) Å and β = 95.047(5)°. In the unit cell there are 4 molecules with formula $C_{18}H_{21}N_3O_4$. Molecules have chiral center at carbon labelled as C10 on Figure 1. Crystal structure is centrosymmetric and therefore racemic. Crystal data, data collection and structure refinement data are given in Table S1. Structural and other crystallographic details on data collection and refinement have been deposited with the Cambridge Crystallographic Data Centre as supplementary publication numbers CCDC Deposition Number 2467779. These data can be obtained free of charge via www.ccdc.cam.ac.uk/conts/retrieving.html (or from the CCDC, 12 Union Road, Cambridge CB2 1EZ, UK; fax: ţ44 1223 336033; e-mail: deposit@ccdc.cam.ac.uk).

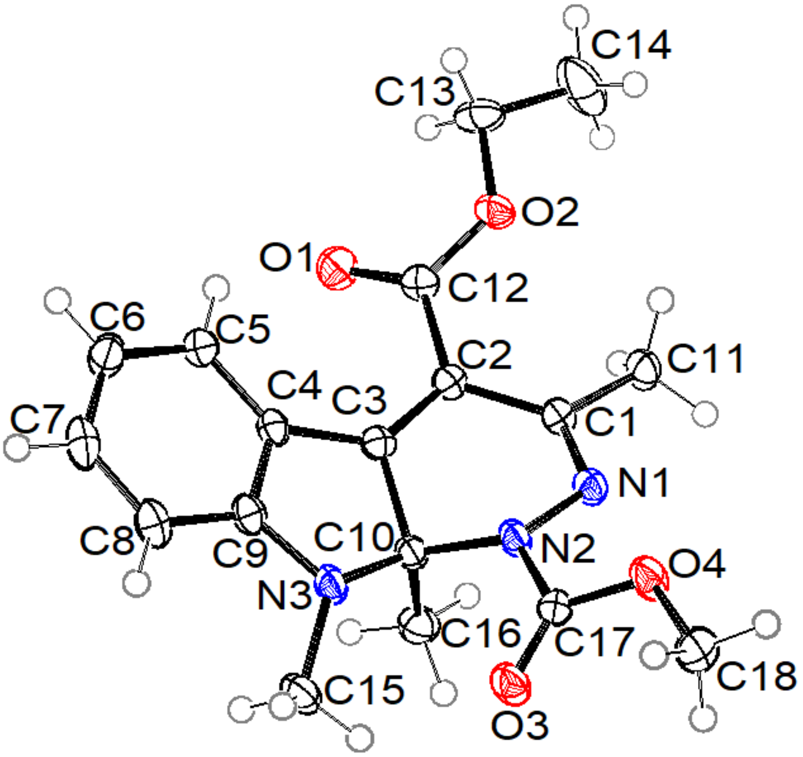

Figure 1. ORTEP diagram of compound **3zh** with ellipsoids at 50 % probability level (CCDC-2467779).

Table S1. Crystal data, data collection and structure refinement for compound 3zh.

Empirical formula	$C_{18}H_{21}N_3O_4$
Formula relative weight	343.38
Temperature/K	150(1)
Crystal system	monoclinic
Space group	$P2_1/c$, no.14
a/Å	14.9413(10)
b/Å	13.7577(11)
c/Å	7.9910(5)
$\alpha = \gamma$/°	90
β/°	95.047(5)
Volume/Å^3	1636.2(2)
Z	4
ρ_{calc}/gcm^{-3}	1.394
μ/mm^{-1}	0.100
F(000)	728
Crystal size/mm^3	0.3 × 0.1 × 0.08
Colour, shape	orange stick
Radiation Mo Kα, λ /Å	0.71073
Θ range - data collection/°	2.74 to 30.41
Index ranges	$-20 \leq h \leq 20$, $-18 \leq k \leq 18$, $-11 \leq l \leq 11$
Reflections collected	13232
Independent reflections	4390 [R_{int} = 0.0475]
Data/parameters	4390/231
Goodness-of-fit on F^2	1.043
Final R indexes	[I>=2σ (I)] R_1 = 0.0507, wR_2 = 0.1129
Final R indexes [all data]	R_1 = 0.0731 wR_2 = 0. 1277
Largest diff. peak/hole /e Å^{-3}	0.28/-0.36

9. References

[1] a) Nemoto, K.; Tanaka, S.; Konno, M.; Onozawa, S.; Chiba, M.; Tanaka, Y.; Sasaki, Y.; Okubo, R.; Hattori, T. Me_2AlCl-mediated carboxylation, ethoxycarbonylation, and carbamoylation of indoles. *Tetrahedron*. **2016**, *72*, 734–745. b) Choy, P. Y.; Lau, C. P.; Kwong, F. Y. Palladium-Catalyzed Direct and Regioselective C−H Bond Functionalization/Oxidative Acetoxylation of Indoles. *J. Org. Chem*. **2011**, *76*, 80–84.

[2] a) Attanasi, O. A.; Filippone, P.; Mei, A; Santeusanio, S. Effect of Metal Ions in Organic Synthesis; Part XXIII. Easy and High-Yield Direct Synthesis of 3-Aminocarbonyl-1-ureidopyrroles by the Copper(II) Chloride-Catalyzed Reaction of Aminocarbonylazoalkenes with 3-Oxoalkanamides. *Synthesis* **1984**, 671−672; b) Attanasi, O. A.; Filippone, P.; Mei, A.; Santeusanio, S. Effect of Metal Ions in Organic Synthesis; Part XXIV. Facile One-Flask Synthesis of 1-Alkoxycarbonylamino-3-aminocarbonylpyrroles by Reaction of Alkoxycarbonylazoalkenes with 3-Oxoalkanamides under Copper(II) Chloride Catalysis. *Synthesis* **1984**, 873−874; c) Preti, L.; Attanasi, O. A.; Caselli, E.; Favi, G.; Ori, C.; Davoli, P.; Felluga, F.; Prati, F. One-Pot Synthesis of Imidazole-4-Carboxylates by Microwave-Assisted 1,5-Electrocyclization of Azavinyl Azomethine Ylides. *Eur. J. Org. Chem.* **2010**, 4312−4320.

[3] Corrieri, M.; De Crescentini, L.; Mantellini, F.; Mari, G.; Santeusanio, S.; Favi, G. Synthesis of Azacarbolines via $PhIO_2$-Promoted Intramolecular Oxidative Cyclization of α-Indolylhydrazones. *J. Org. Chem.* **2021**, *86*, 17918−17929.

[4] a) Qi, L.-W.; Mao, J.-H.; Zhang, J.; Tan, B. Organocatalytic asymmetric arylation of indoles enabled by azo groups. *Nat. Chem*. **2018**, *10*, 58−64. b) Barrett, K. T.; Metrano, A. J.; Rablen, P. R.; Miller, S. J. Spontaneous transfer of chirality in an atropisomerically enriched two-axis system. *Nature.* **2014**, *509*, 71−75.

[5] CrysAlisPro 1.171.42.90a, Rigaku Oxford Diffraction, Rigaku Corporation, Oxford, UK, 2023.

[6] Burla, M. C.; Caliandro, R.; Carrozzini, B.; Cascarano, G. L.; Cuocci, C.; Giacovazzo, C.; Mallamo, M.; Mazzone, A.; Polidori, G. Crystal structure determination and refinement via SIR2014. *J. Appl. Cryst.* **2015**, *48*, 306–309.

[7] Sheldrick, G. M. Crystal structure refinement with SHELXL. *Acta Cryst.* **2015**, *71*, 3–8.

[8] Farrugia, L. J. ORTEP-3 for Windows—A Version of ORTEP-III with a Graphical User Interface (GUI). *J. Appl. Crystallogr.* **1997**, *30*, 565–567.

10. ^{1}H and ^{13}C{^{1}H} NMR

Methyl 2-(4-ethoxy-3-(2-methyl-1*H*-indol-3-yl)-4-oxobutan-2-ylidene)hydrazinecarboxylate (A1):

^{1}H NMR (400 MHz, DMSO-d_6) of **A1**:

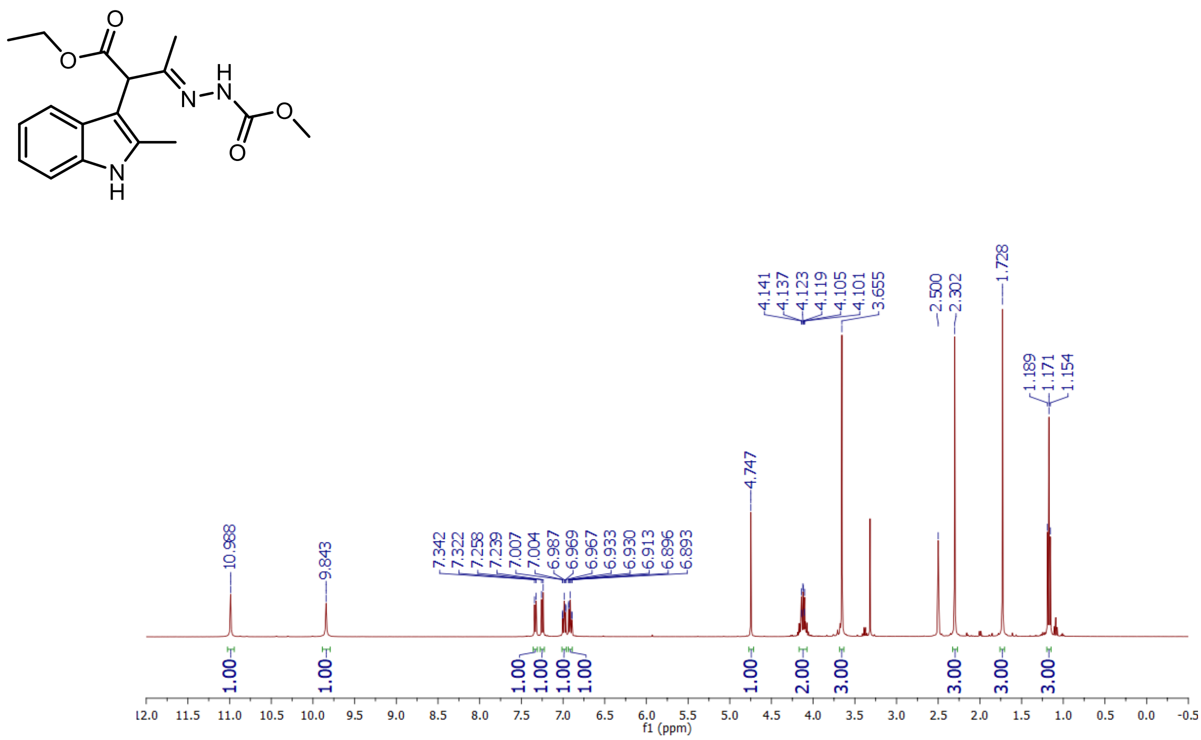

^{13}C{^{1}H} NMR (101 MHz, DMSO-d_6) of **A1**:

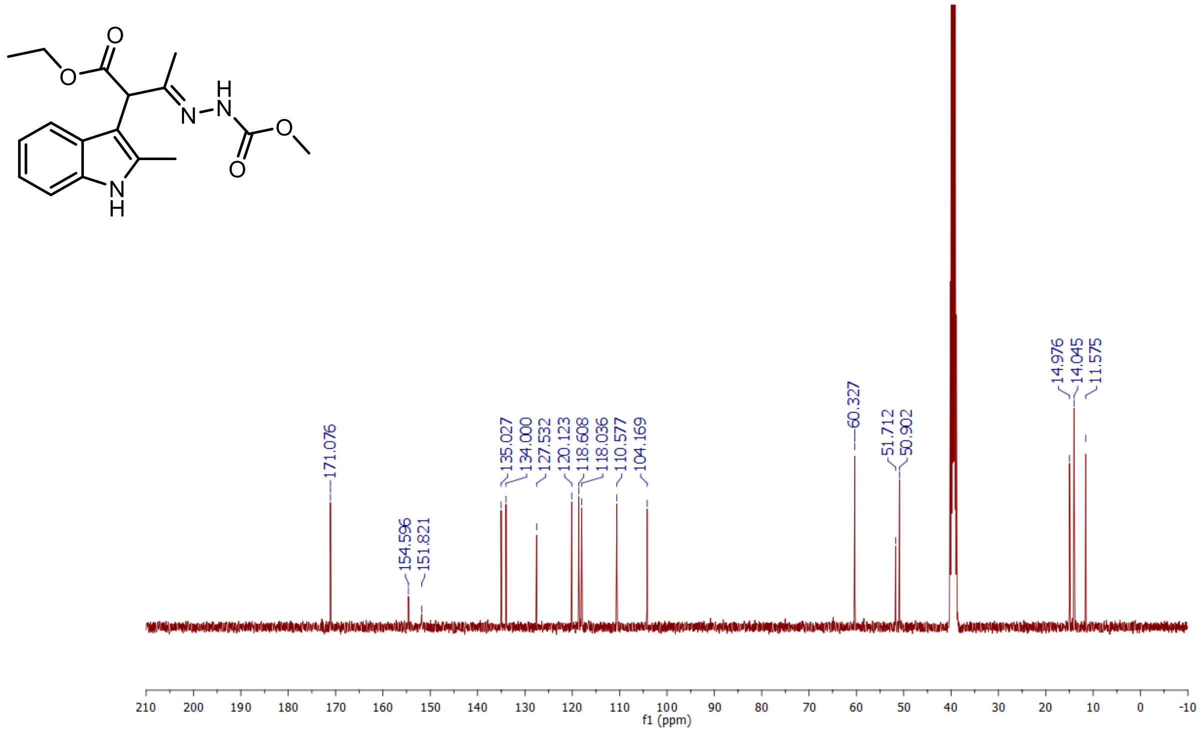

Methyl 2-(4-ethoxy-3-(2-methyl-1*H*-indol-3-yl)-4-oxobut-2-en-2-yl)hydrazine-1-carboxylate (A1')

^{1}H NMR (400 MHz, DMSO-d_6) of **A1'**:

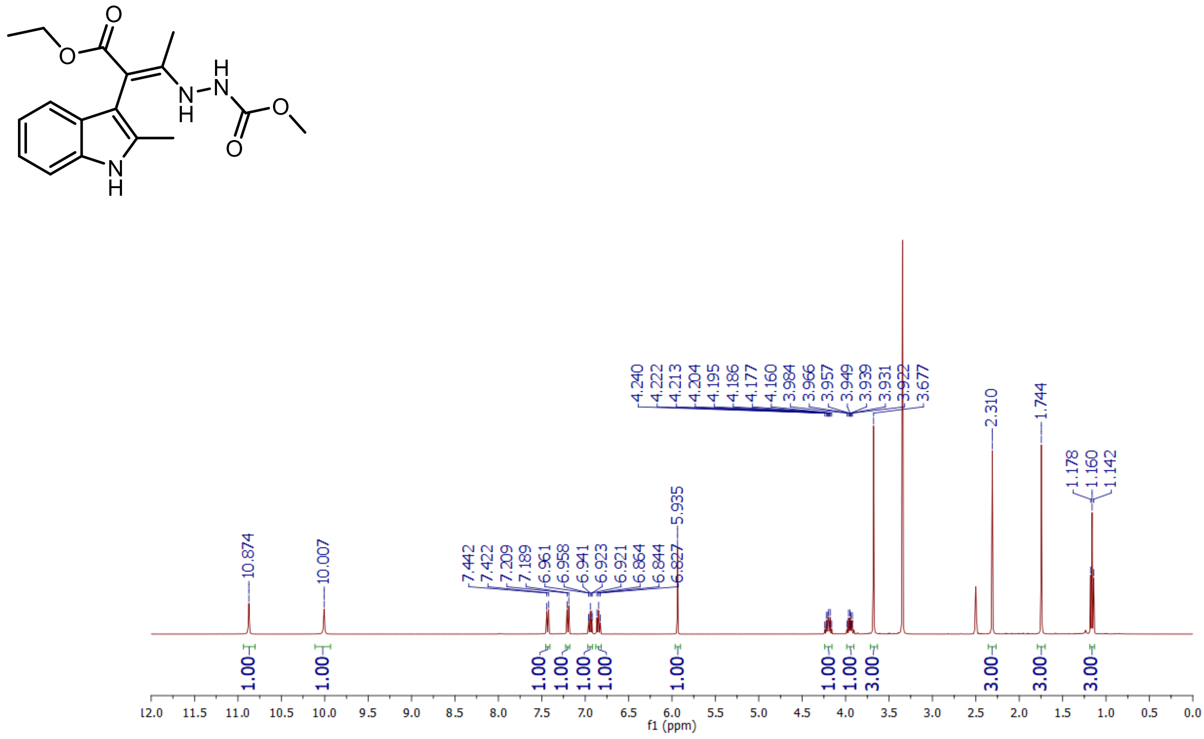

^{13}C{^{1}H} NMR (101 MHz, DMSO-d_6) of **A1'**:

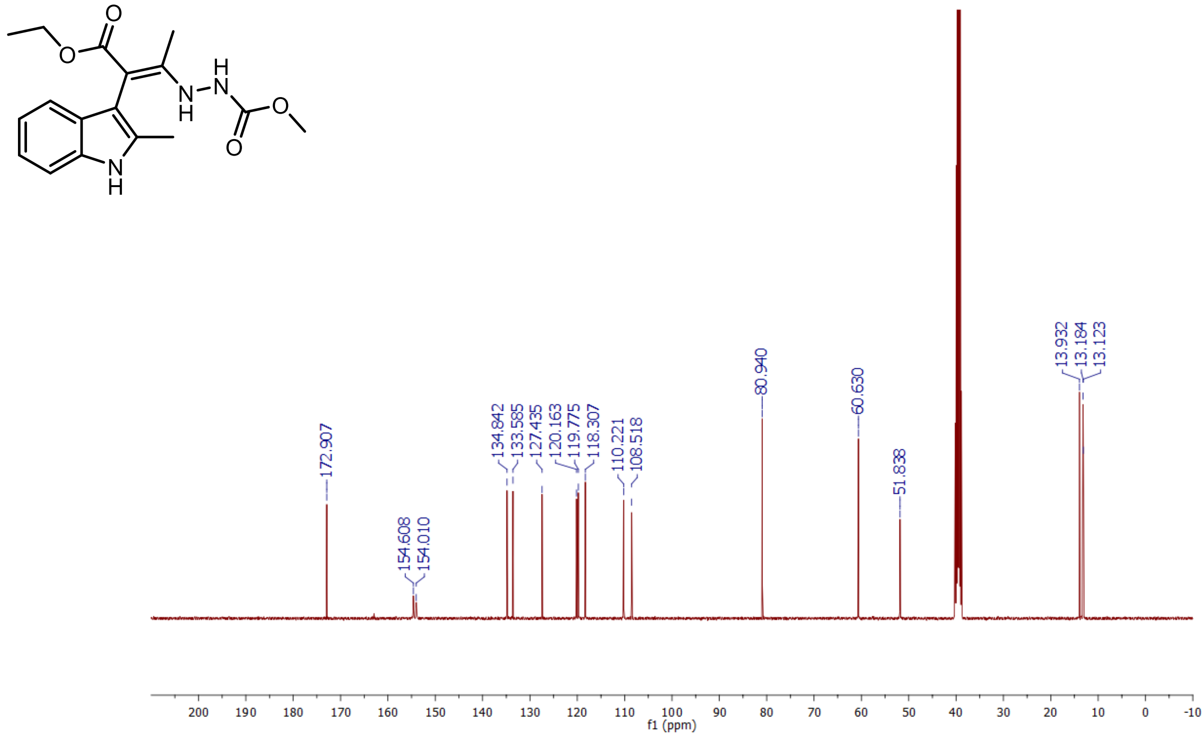

4-Ethyl 1-methyl 3,9a-dimethyl-9,9a-dihydro-1*H*-pyridazino[3,4-*b*]indole-1,4-dicarboxylate (3a):
^{1}H NMR (400 MHz, DMSO-d_6) of **3a**:

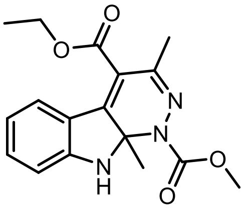

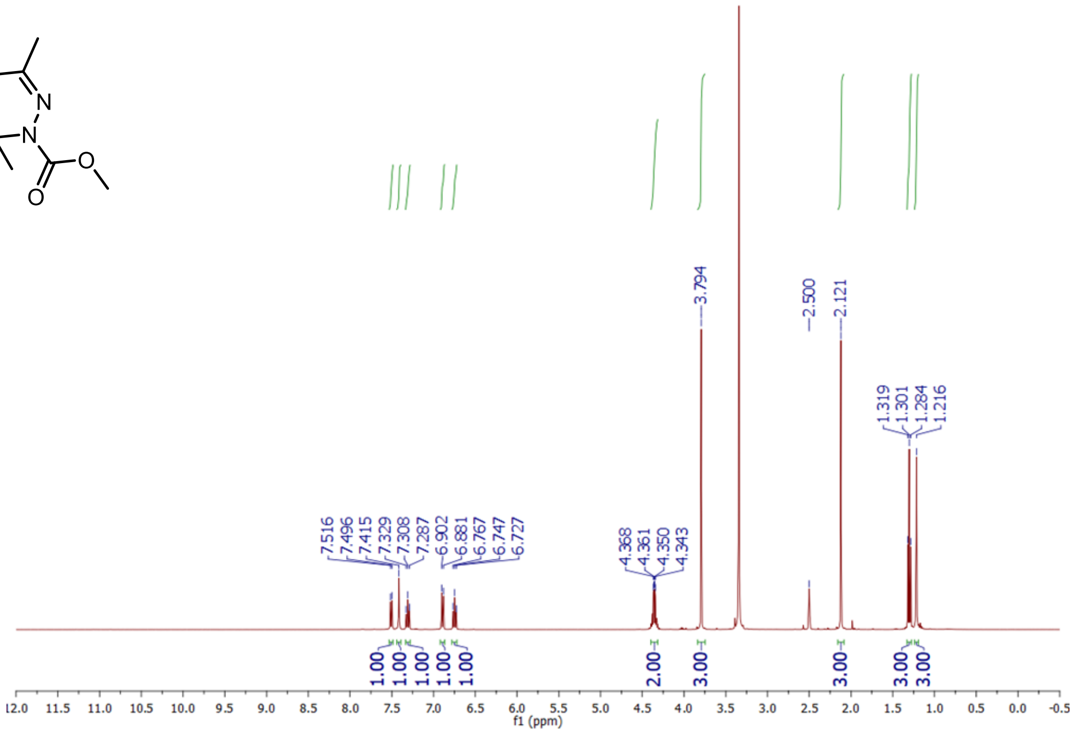

^{13}C{^{1}H} NMR (101 MHz, DMSO-d_6) of **3a**:

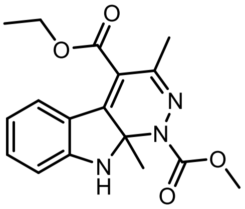

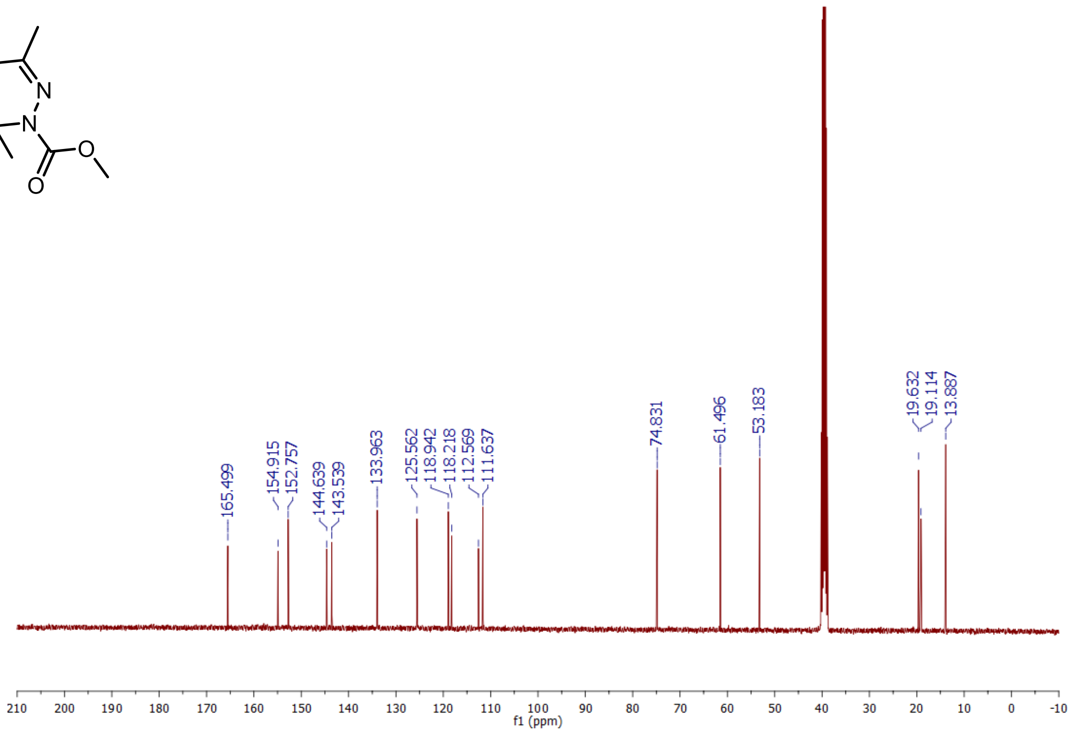

HMQC NMR (400 MHz, DMSO-d_6) of **3a**:

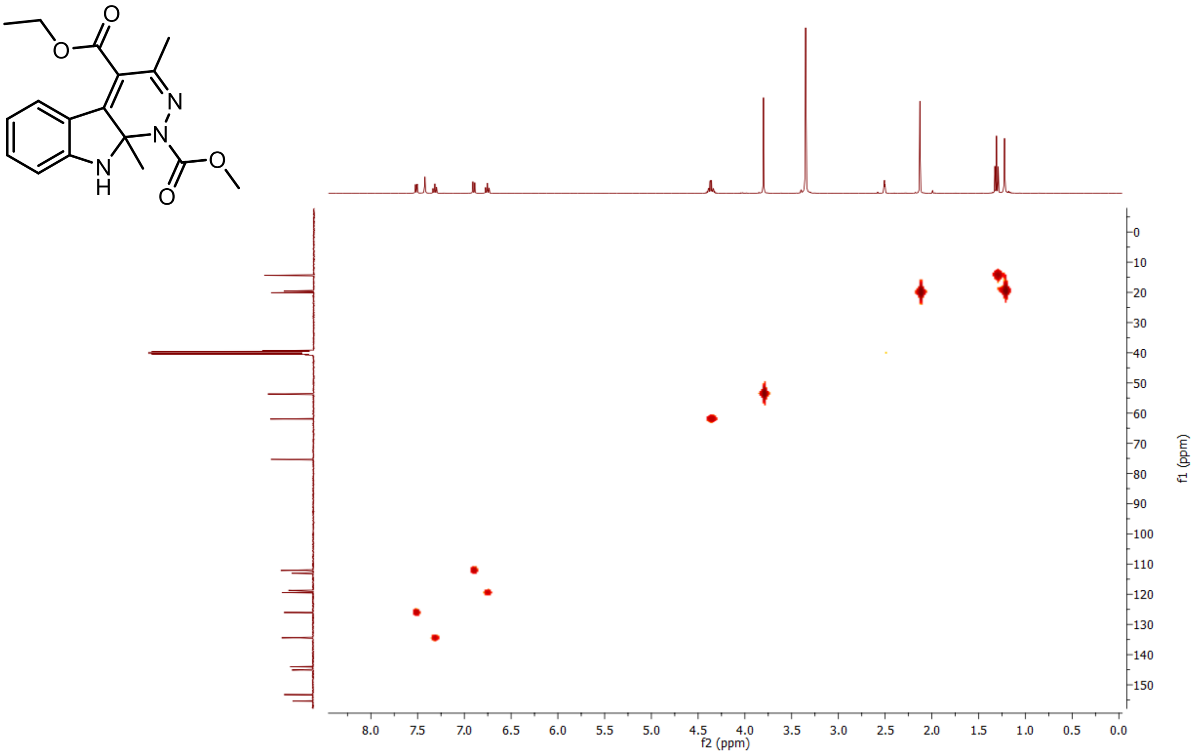

HMBC NMR (400 MHz, DMSO-d_6) of **3a**:

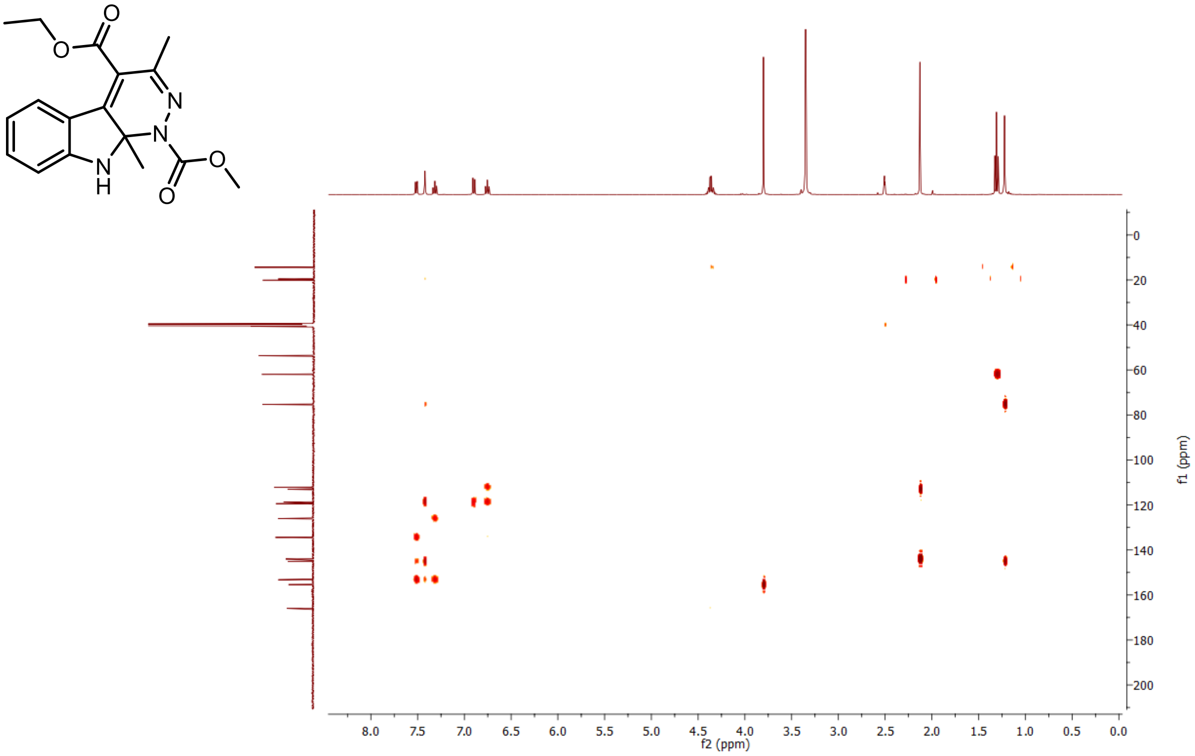

Diethyl 3,9a-dimethyl-9,9a-dihydro-1*H*-pyridazino[3,4-*b*]indole-1,4-dicarboxylate (3b):

^{1}H NMR (400 MHz, DMSO-d_6) of **3b**:

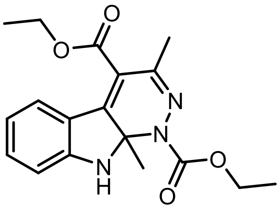

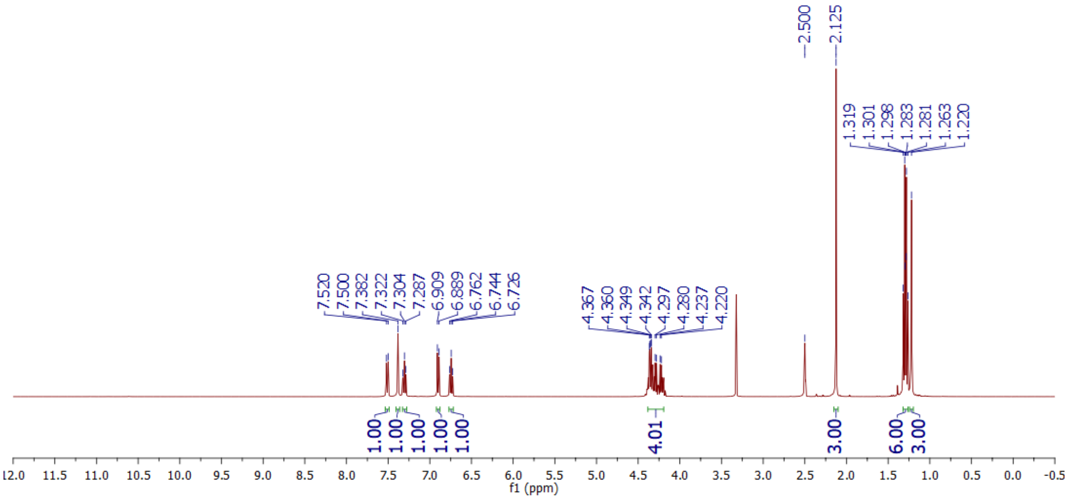

^{13}C{^{1}H} NMR (101 MHz, DMSO-d_6) of **3b**:

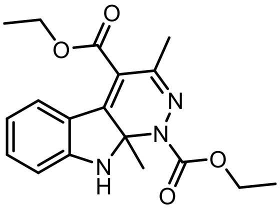

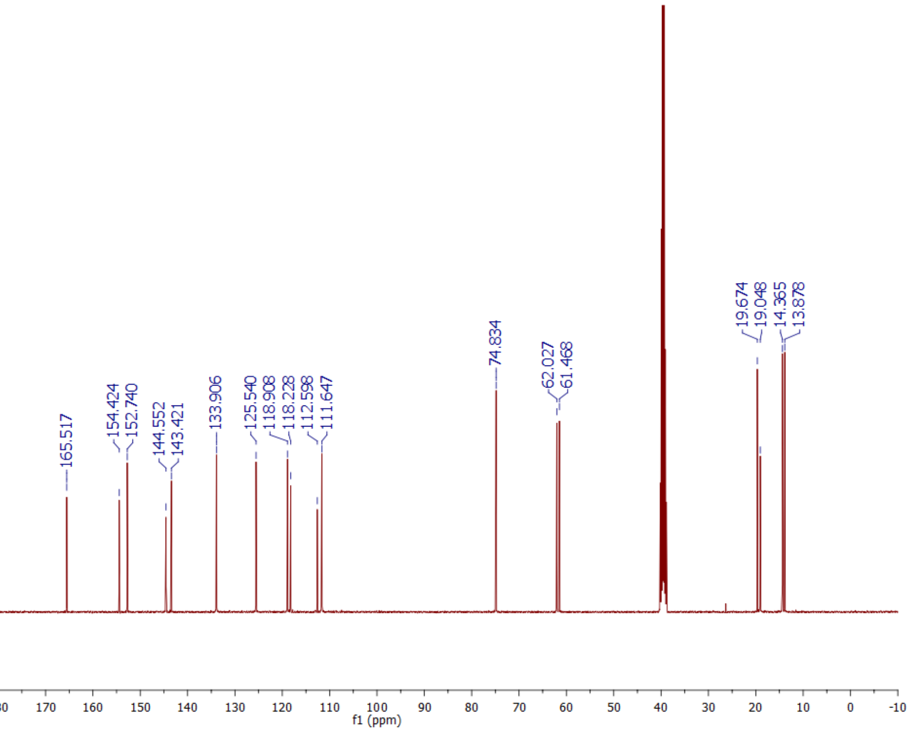

Dimethyl 3,9a-dimethyl-9,9a-dihydro-1*H*-pyridazino[3,4-*b*]indole-1,4-dicarboxylate (3c):

^{1}H NMR (400 MHz, DMSO-d_6) of **3c**:

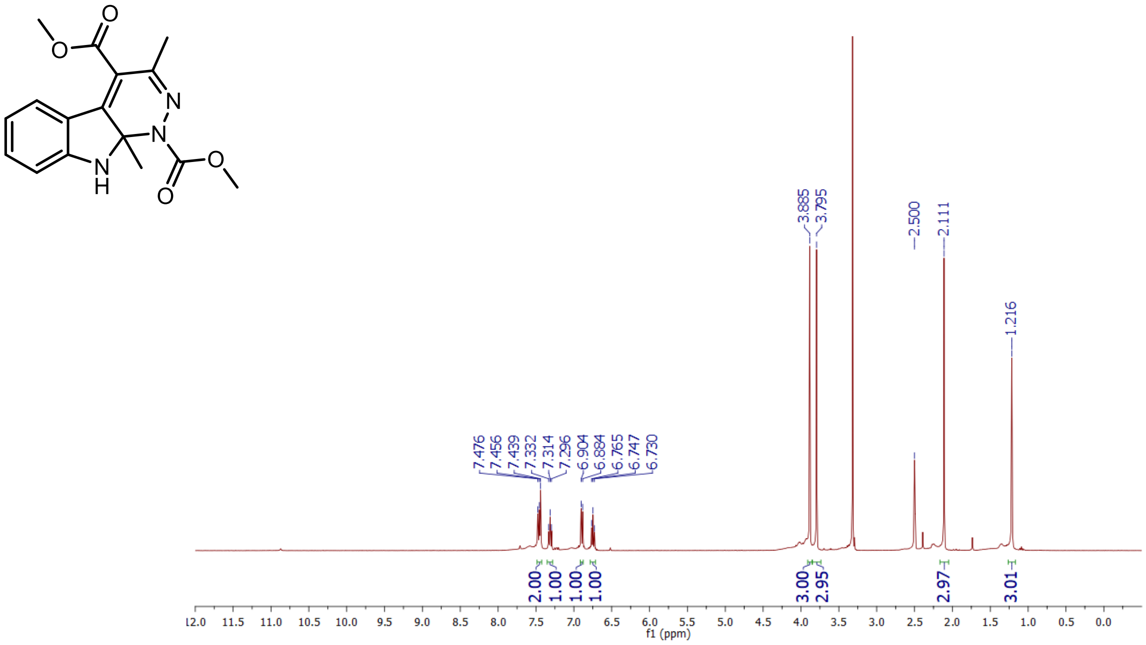

^{13}C{^{1}H} NMR (101 MHz, DMSO-d_6) of **3c**:

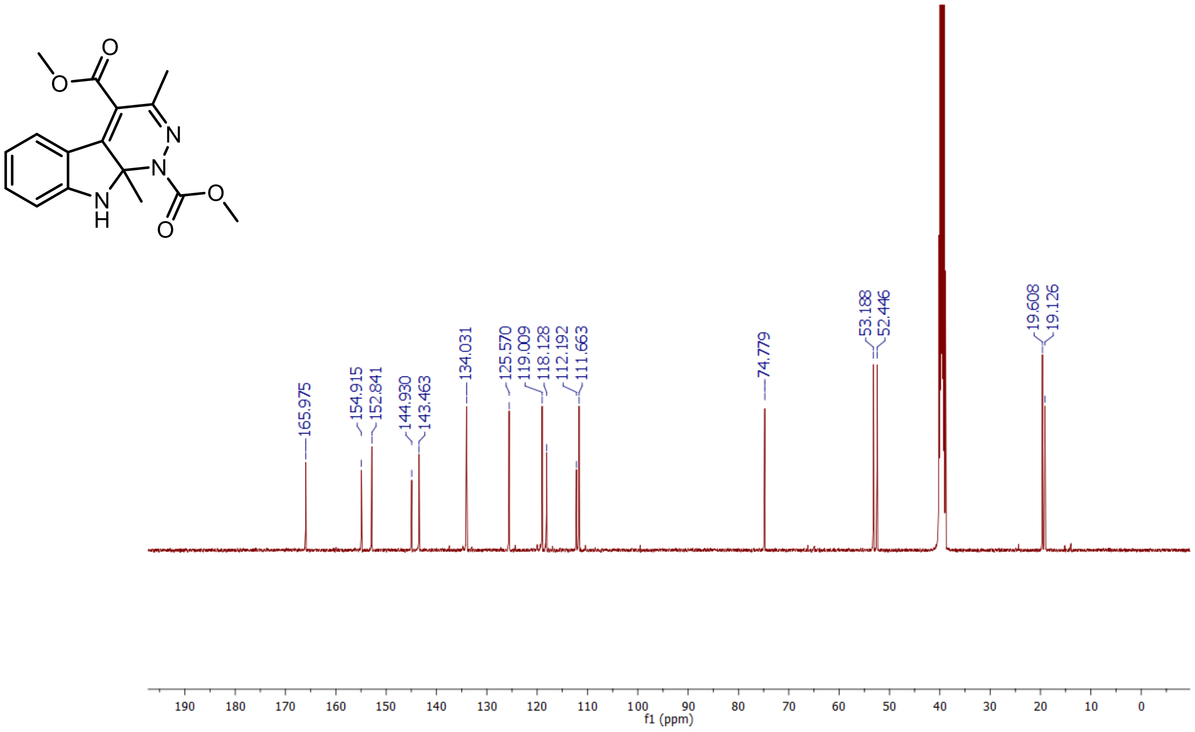

1-(*Tert*-butyl) 4-ethyl 3,9a-dimethyl-9,9a-dihydro-1*H*-pyridazino[3,4-*b*]indole-1,4-dicarboxylate (3d):

^{1}H NMR (400 MHz, DMSO-d_6) of **3d**:

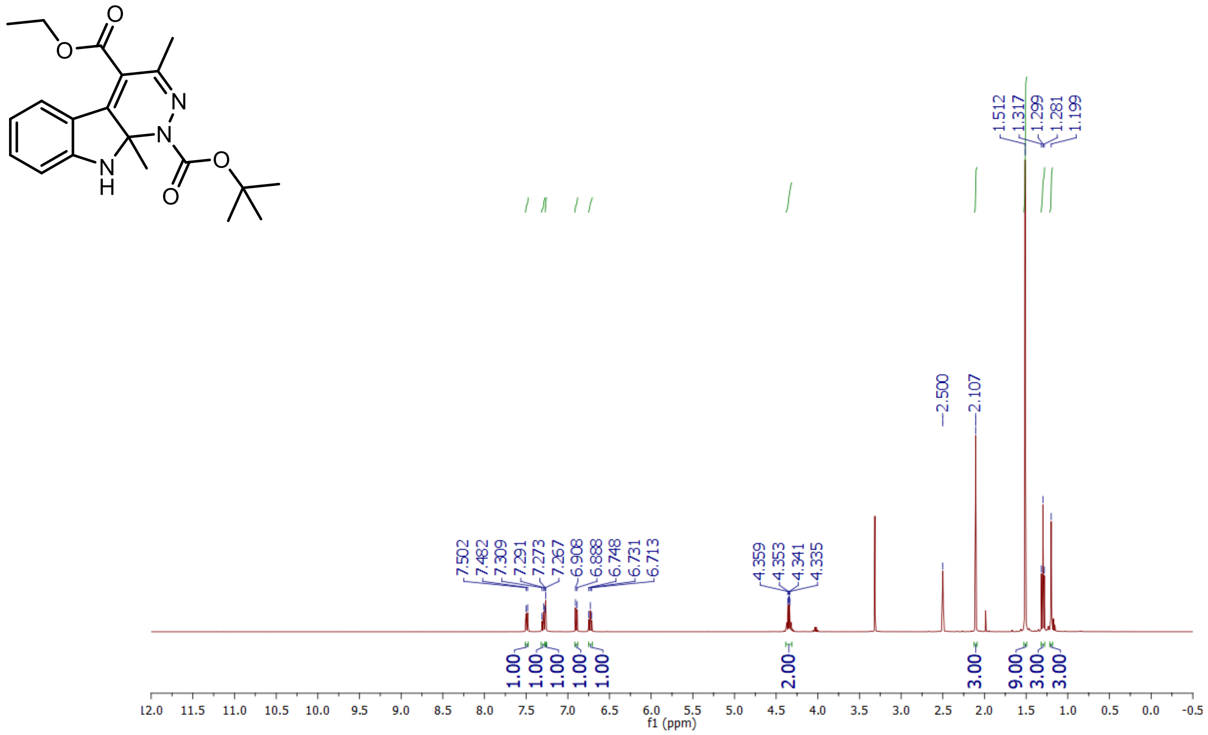

^{13}C{^{1}H} NMR (101 MHz, DMSO-d_6) of **3d**:

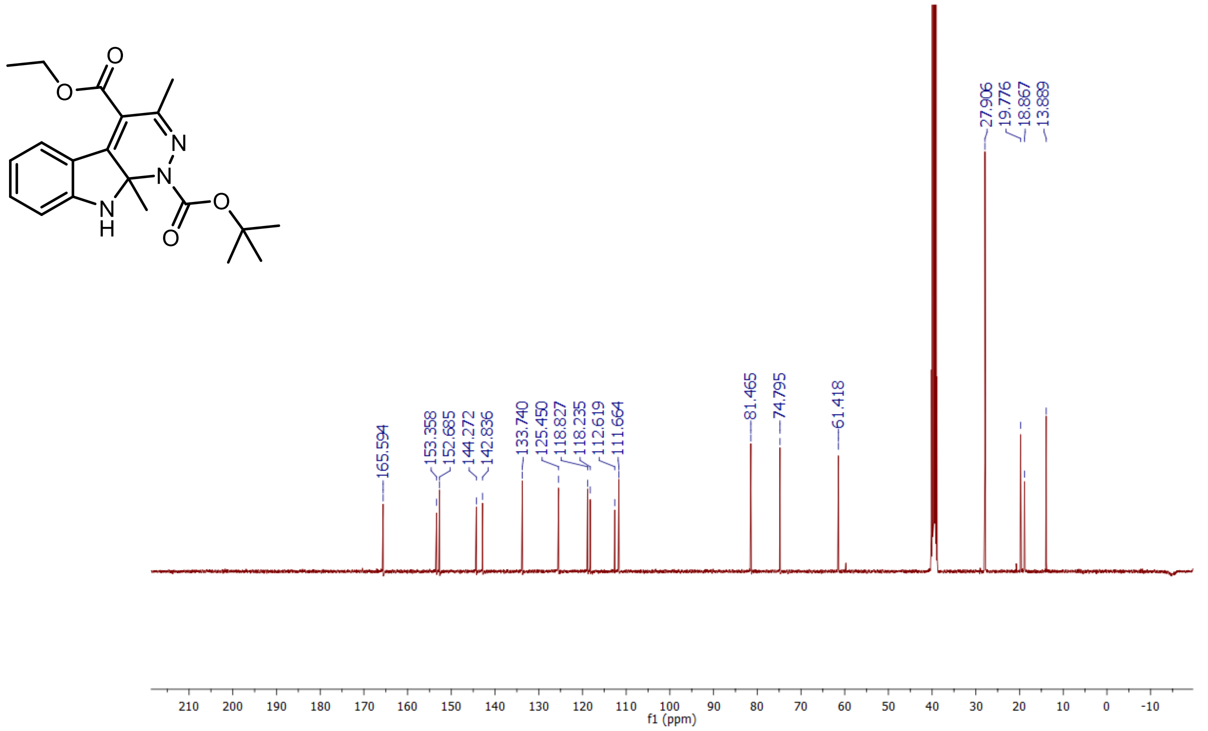

1-(*Tert*-butyl) 4-methyl 3,9a-dimethyl-9,9a-dihydro-1*H*-pyridazino[3,4-*b*]indole-1,4-dicarboxylate (3e):

^{1}H NMR (400 MHz, DMSO-d_6) of **3e**:

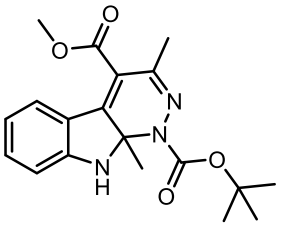

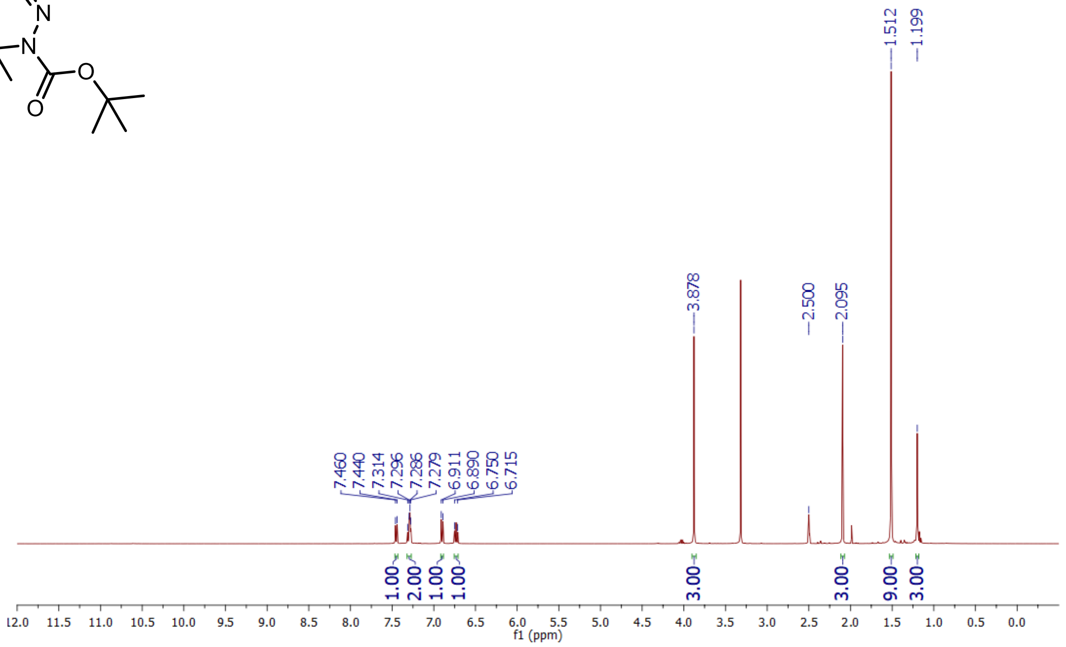

^{13}C{^{1}H} NMR (101 MHz, DMSO-d_6) of **3e**:

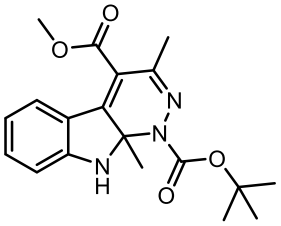

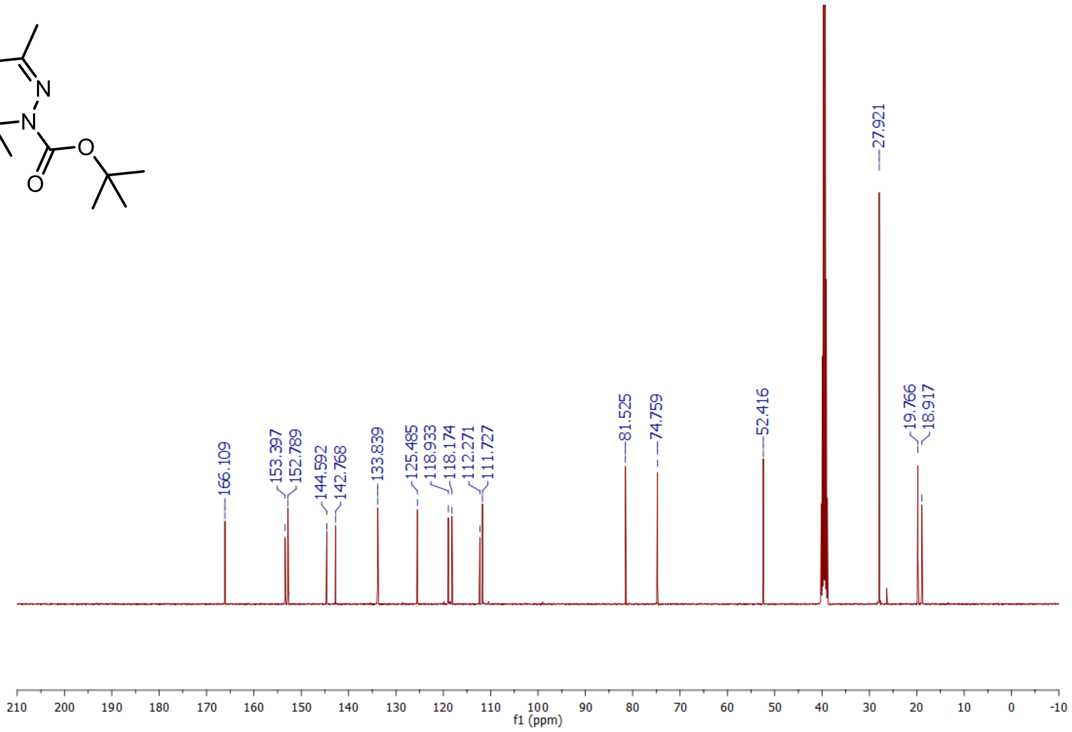

1-(*Tert*-butyl) 4-isopropyl 3,9a-dimethyl-9,9a-dihydro-1*H*-pyridazino[3,4-*b*]indole-1,4-dicarboxylate (3f):

^{1}H NMR (400 MHz, DMSO-d_6) of **3f**:

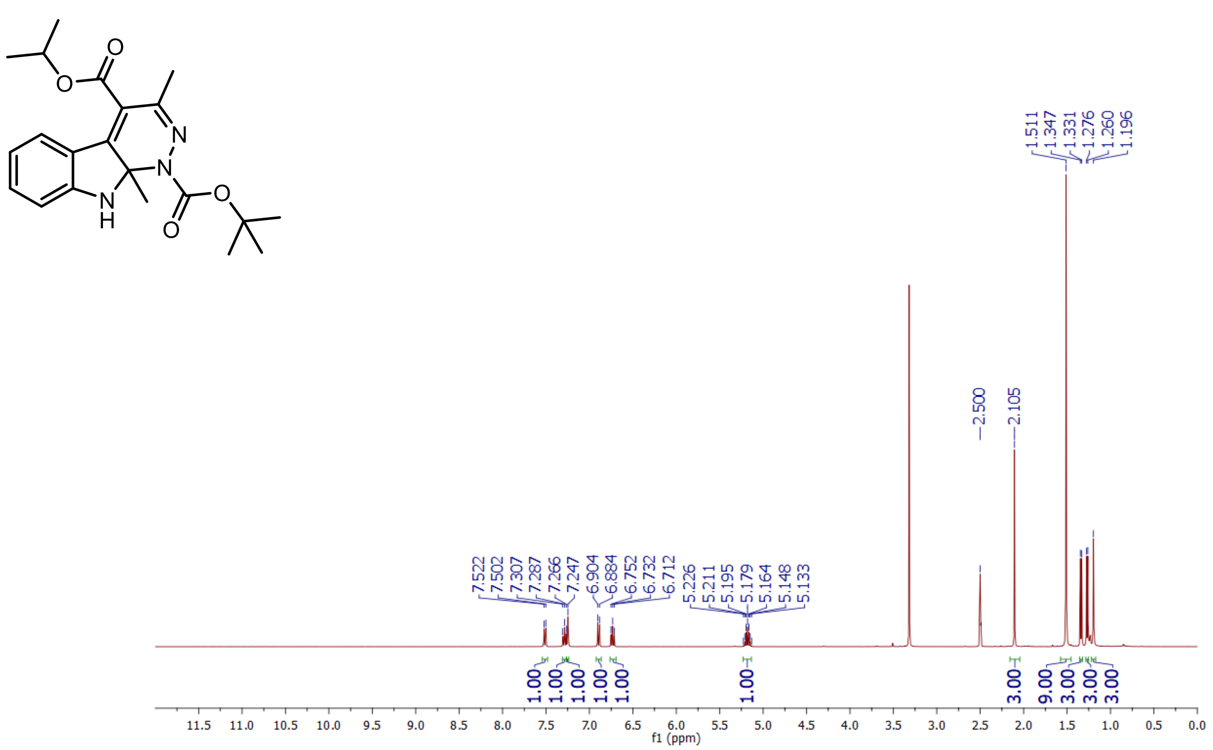

^{13}C{^{1}H} NMR (101 MHz, DMSO-d_6) of **3f**:

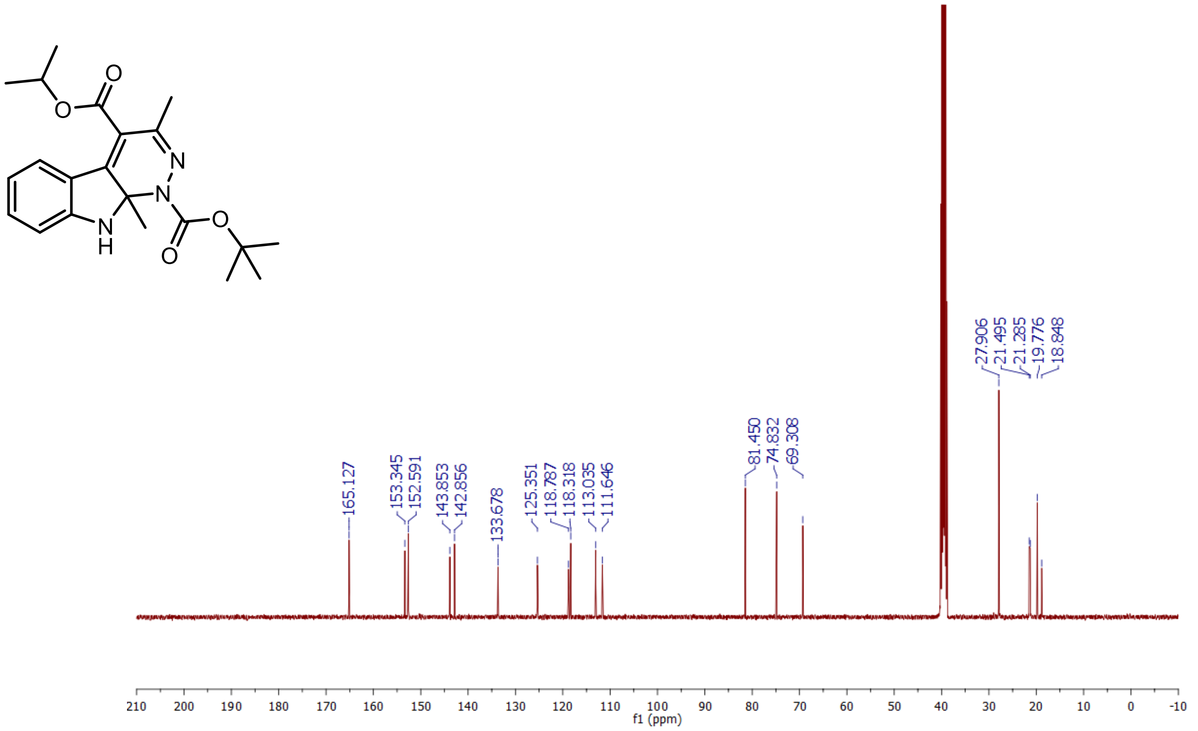

Di-*tert*-butyl 3,9a-dimethyl-9,9a-dihydro-1*H*-pyridazino[3,4-*b*]indole-1,4-dicarboxylate (3g):

^{1}H NMR (400 MHz, DMSO-d_6) of **3g**:

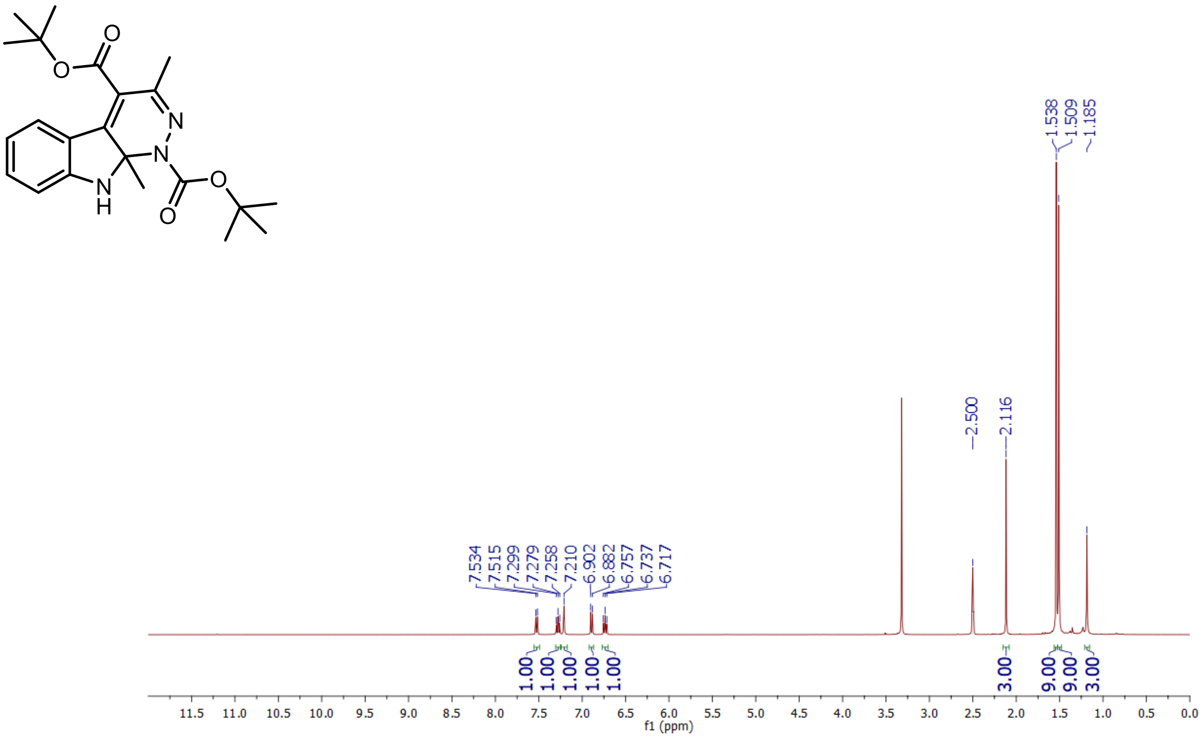

^{13}C{^{1}H} NMR (101 MHz, DMSO-d_6) of **3g**:

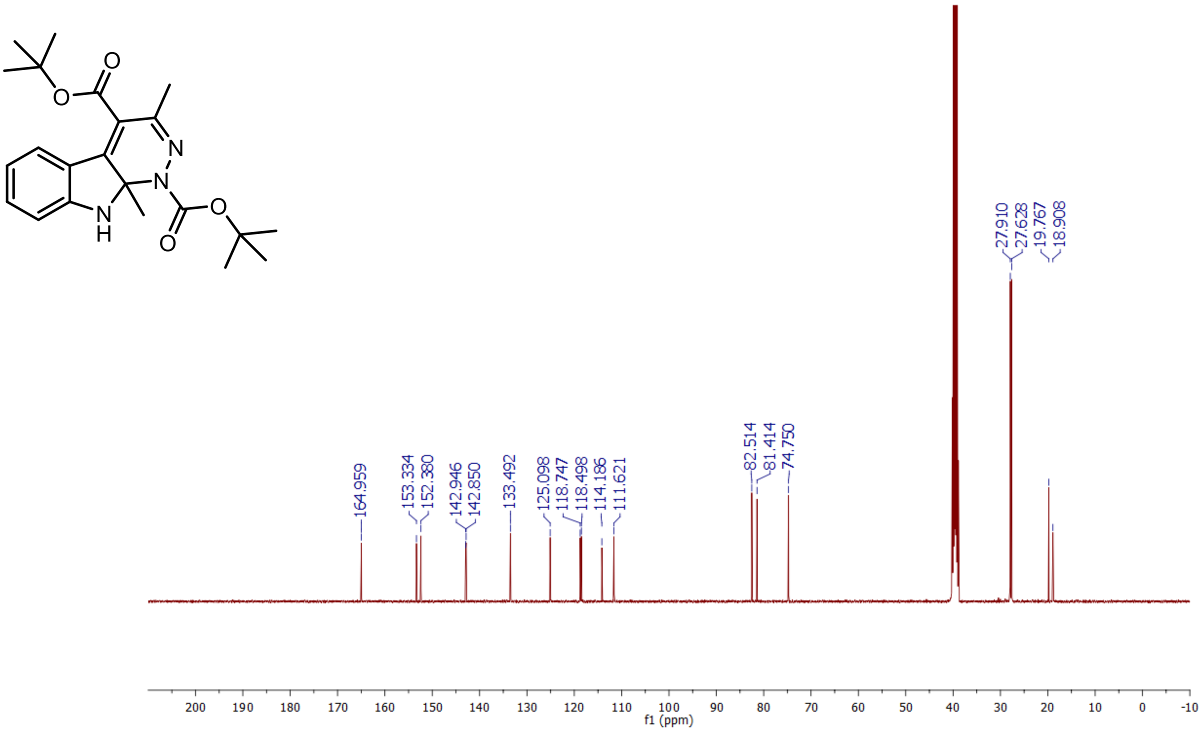

4-Benzyl 1-methyl 3,9a-dimethyl-9,9a-dihydro-1*H*-pyridazino[3,4-*b*]indole-1,4-dicarboxylate (3h):

^{1}H NMR (400 MHz, DMSO-d_6) of **3h**:

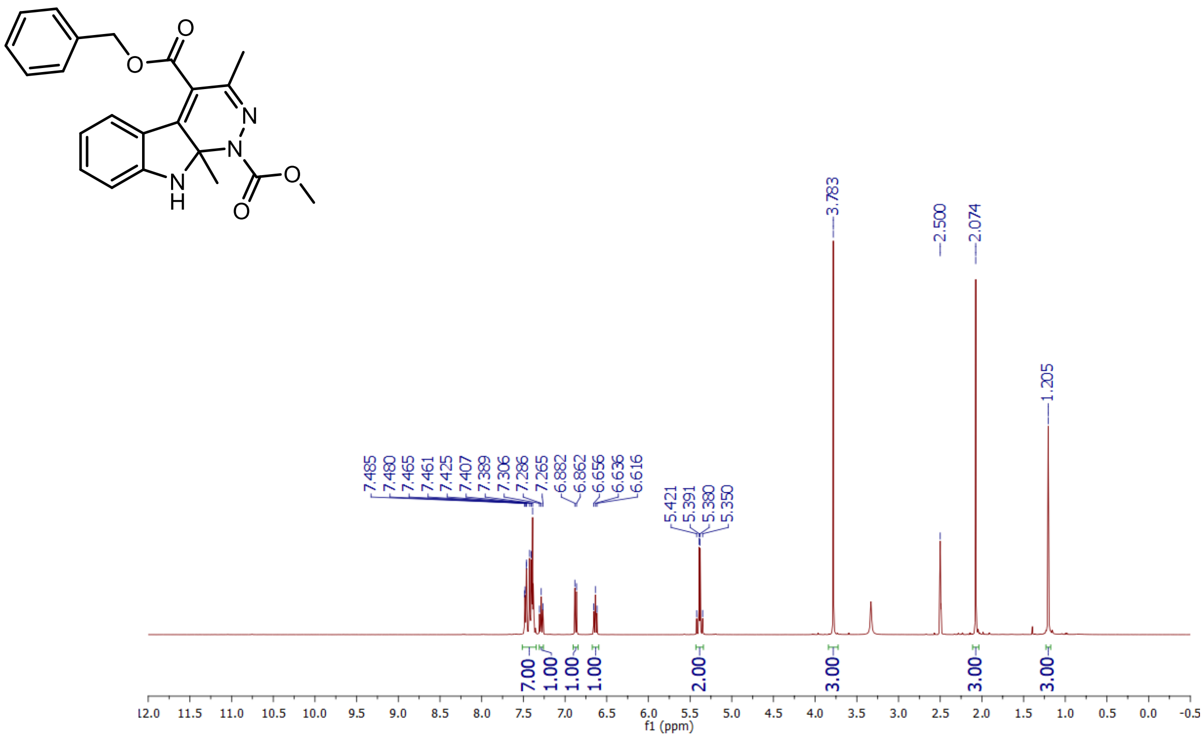

^{13}C{^{1}H} NMR (101 MHz, DMSO-d_6) of **3h**:

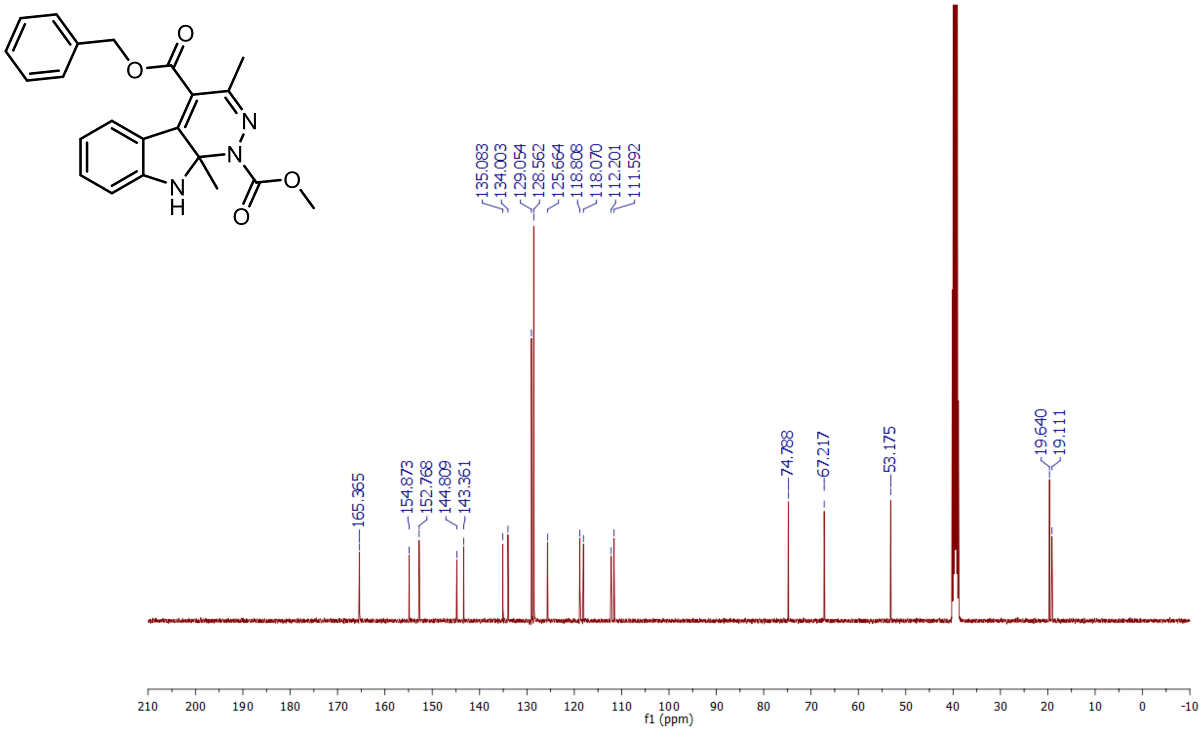

1-Ethyl 4-(2-methoxyethyl) 3,9a-dimethyl-9,9a-dihydro-1*H*-pyridazino[3,4-*b*]indole-1,4-dicarboxylate (3i):

^{1}H NMR (400 MHz, DMSO-d_6) of **3i**:

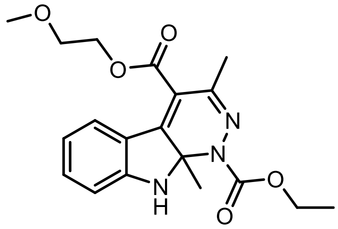

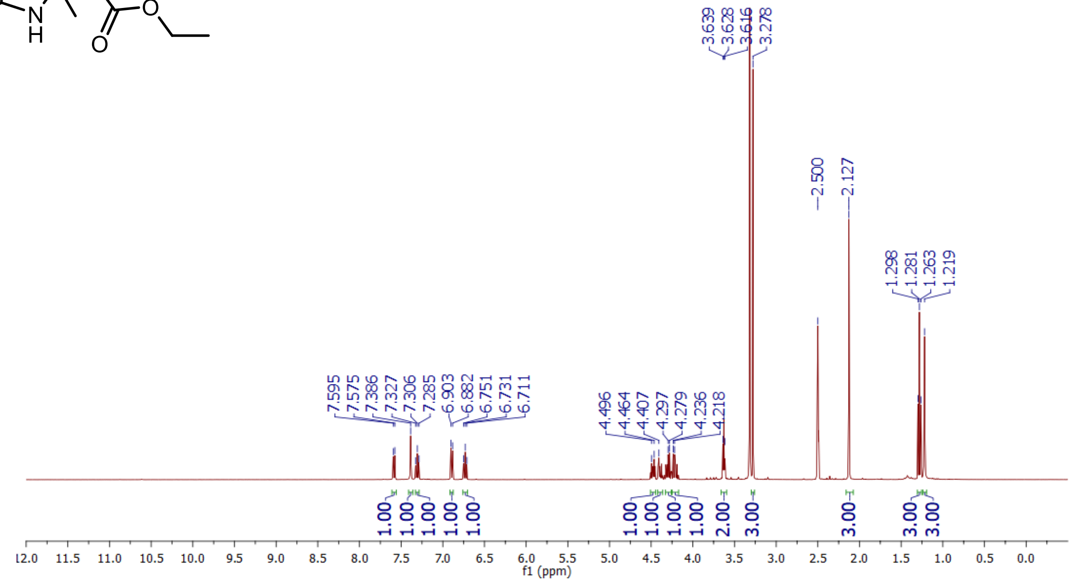

^{13}C{^{1}H} NMR (101 MHz, DMSO-d_6) of **3i**:

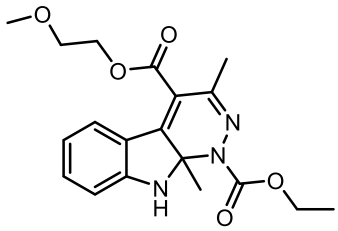

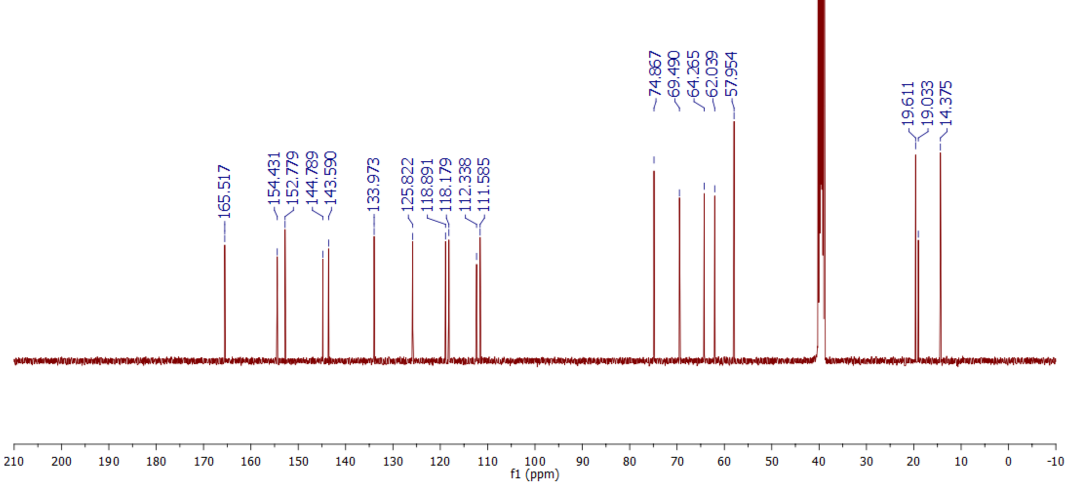

1-(*Tert*-butyl) 4-ethyl 9a-methyl-3-propyl-9,9a-dihydro-1*H*-pyridazino[3,4-*b*]indole-1,4-dicarboxylate (3j):

^{1}H NMR (400 MHz, DMSO-d_6) of **3j**:

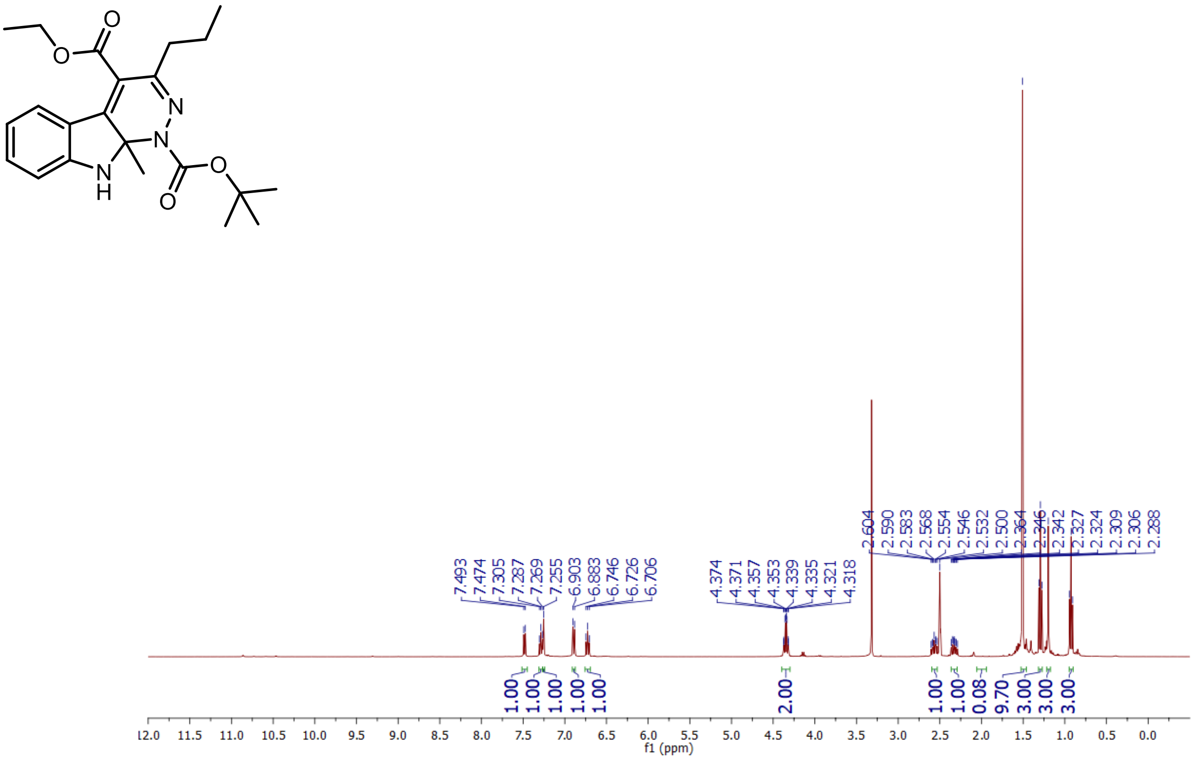

^{13}C{^{1}H} NMR (101 MHz, DMSO-d_6) of **3j**:

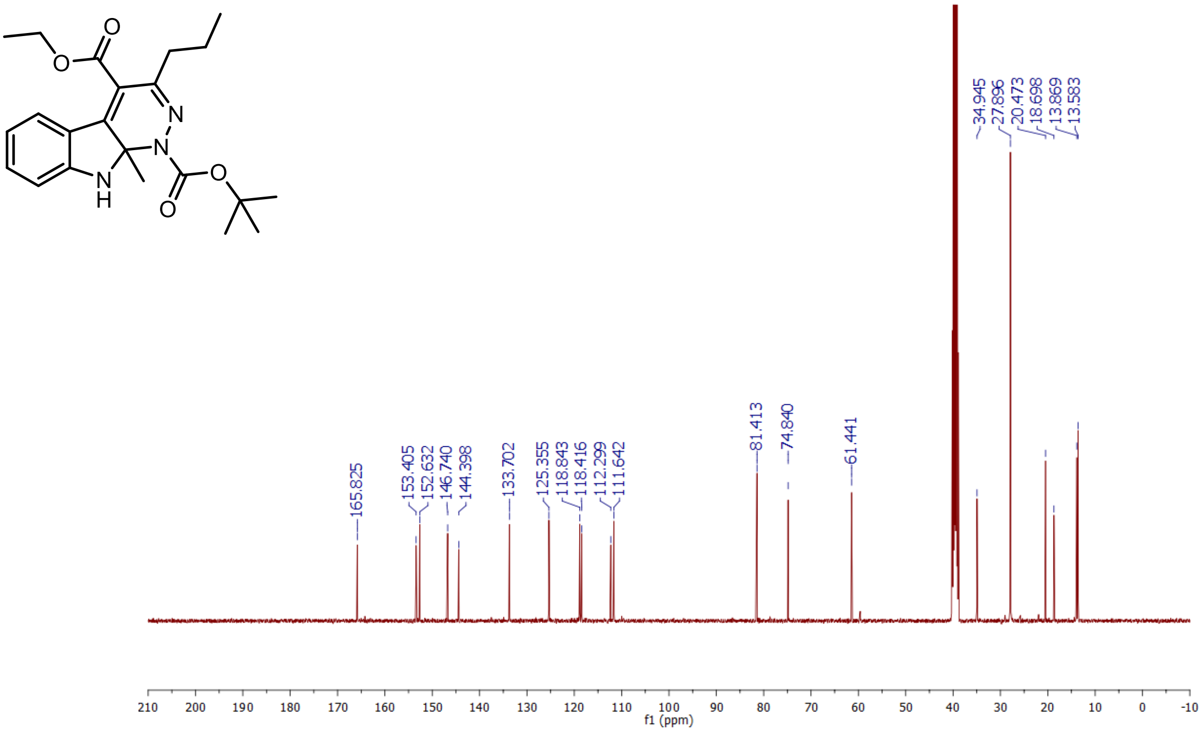

1-Methyl 4-propyl 3,9a-dimethyl-9,9a-dihydro-1*H*-pyridazino[3,4-*b*]indole-1,4-dicarboxylate (3k):

^{1}H NMR (400 MHz, DMSO-d_6) of **3k**:

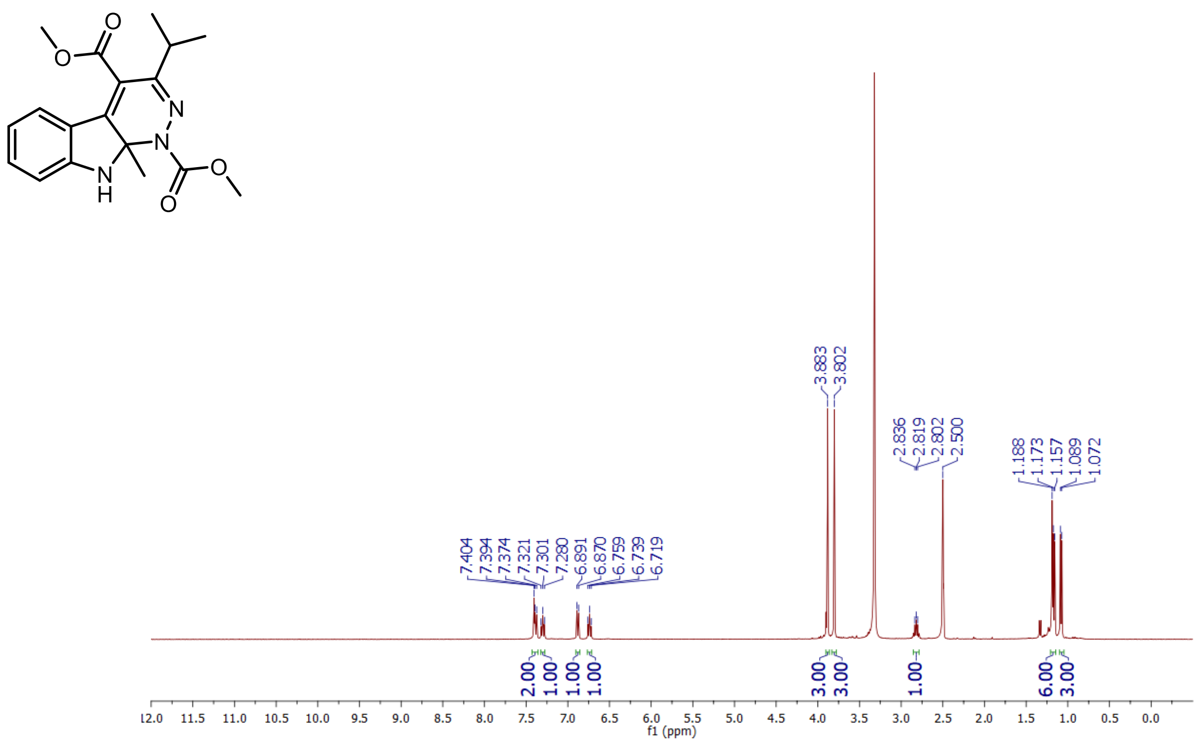

^{13}C{^{1}H} NMR (101 MHz, DMSO-d_6) of **3k**:

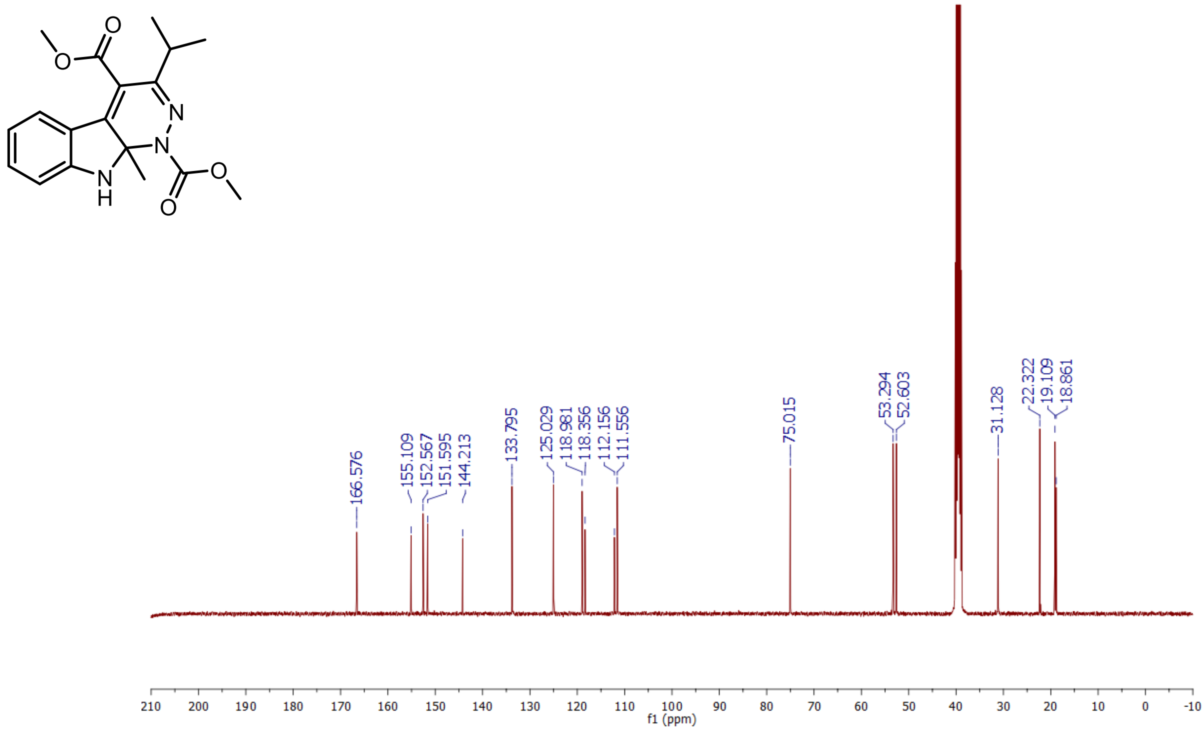

Methyl 4-(dimethylcarbamoyl)-3,9a-dimethyl-9,9a-dihydro-1*H*-pyridazino[3,4-*b*]indole-1-carboxylate (3l):

^{1}H NMR (400 MHz, DMSO-d_6) of **3l**:

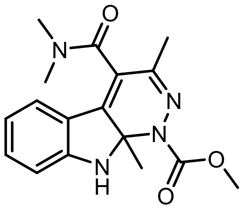

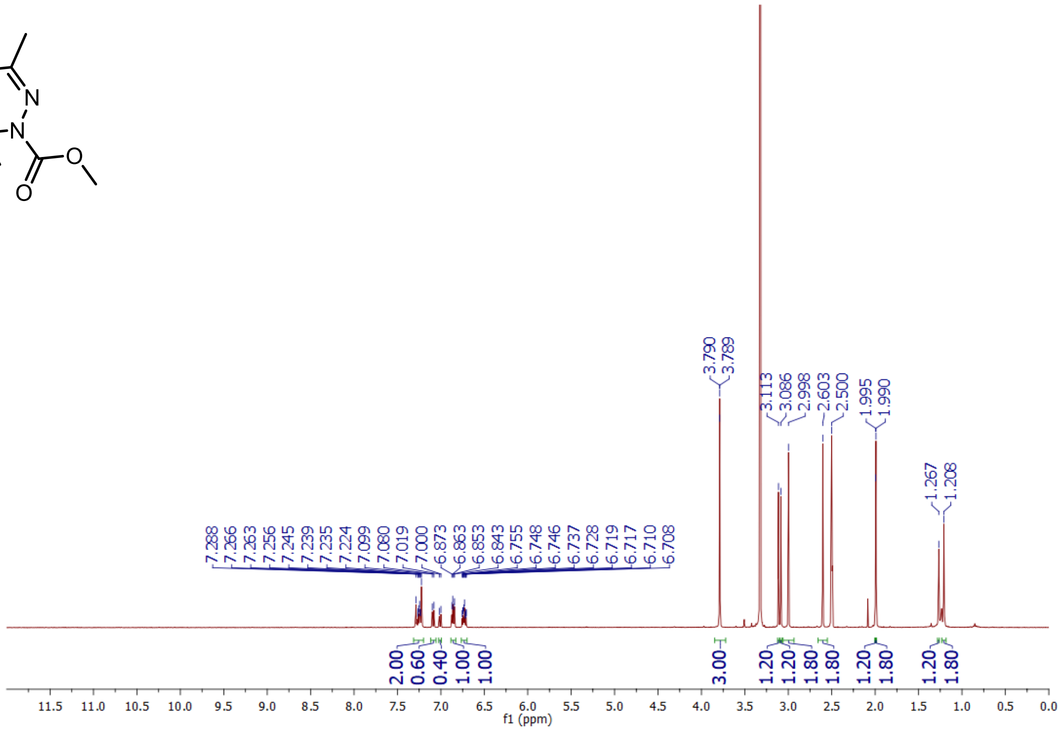

^{13}C{^{1}H} NMR (101 MHz, DMSO-d_6) of **3l**:

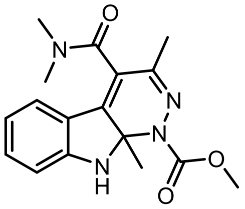

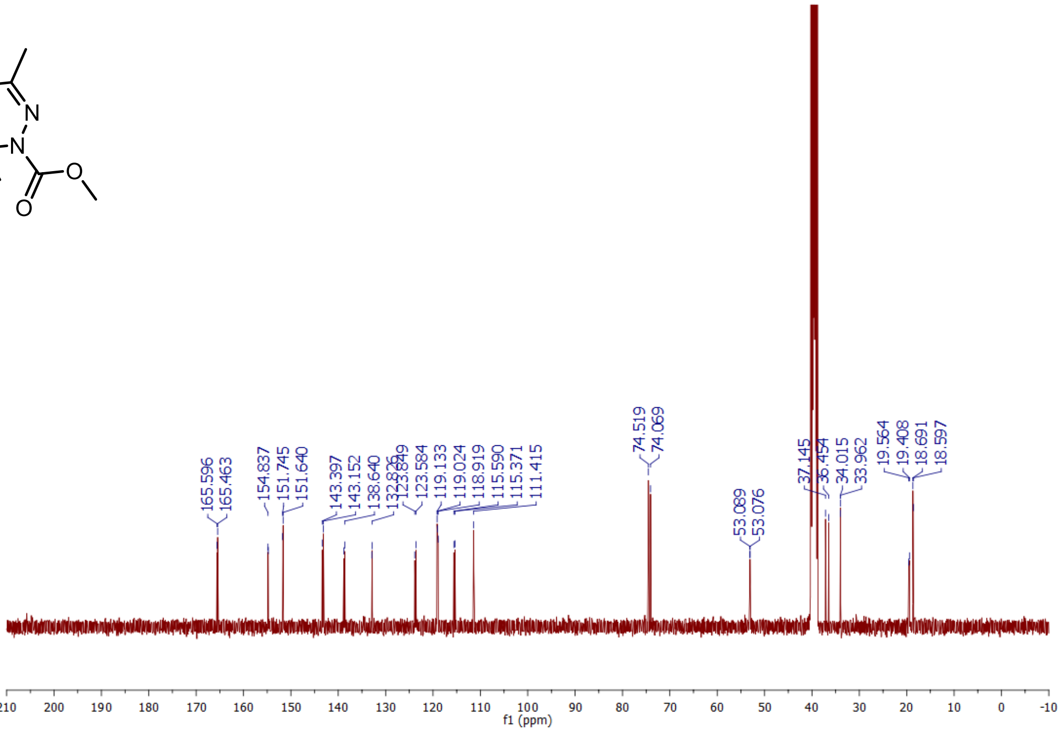

Benzyl 4-carbamoyl-3,9a-dimethyl-9,9a-dihydro-1*H*-pyridazino[3,4-*b*]indole-1-carboxylate (3m):
^{1}H NMR (400 MHz, DMSO-d_6) of **3m**:

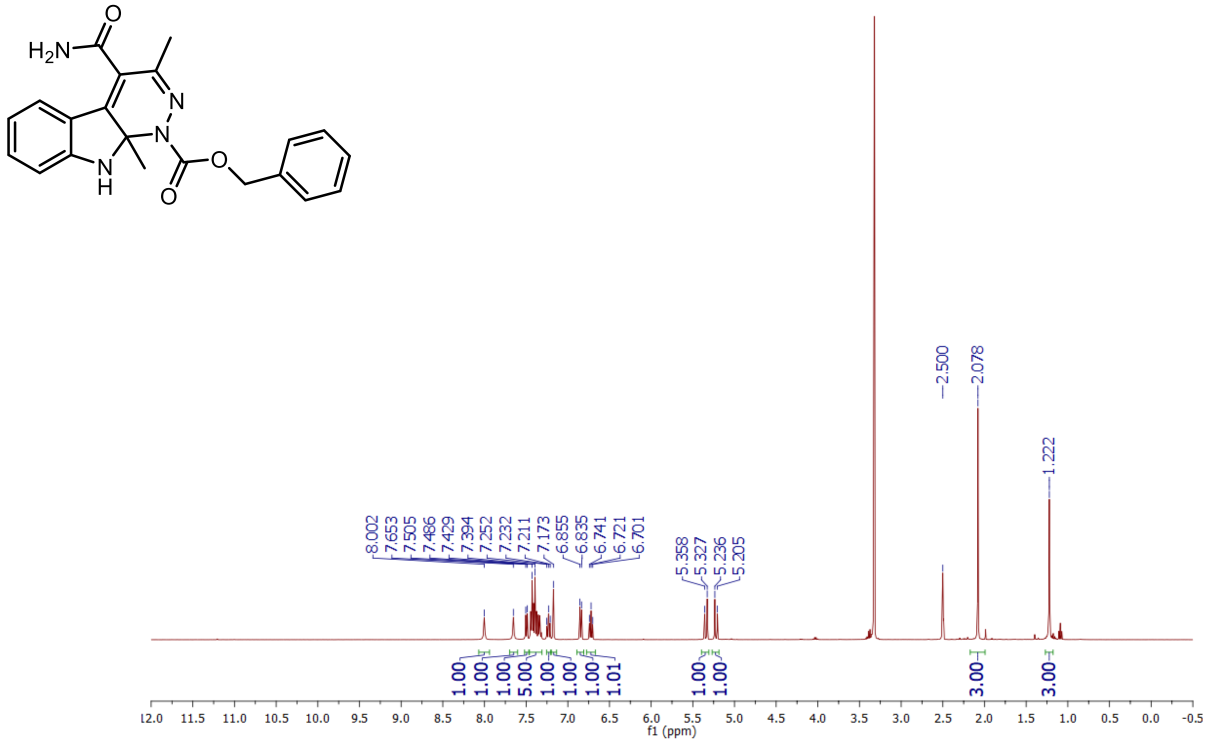

^{13}C{^{1}H} NMR (101 MHz, DMSO-d_6) of **3m**:

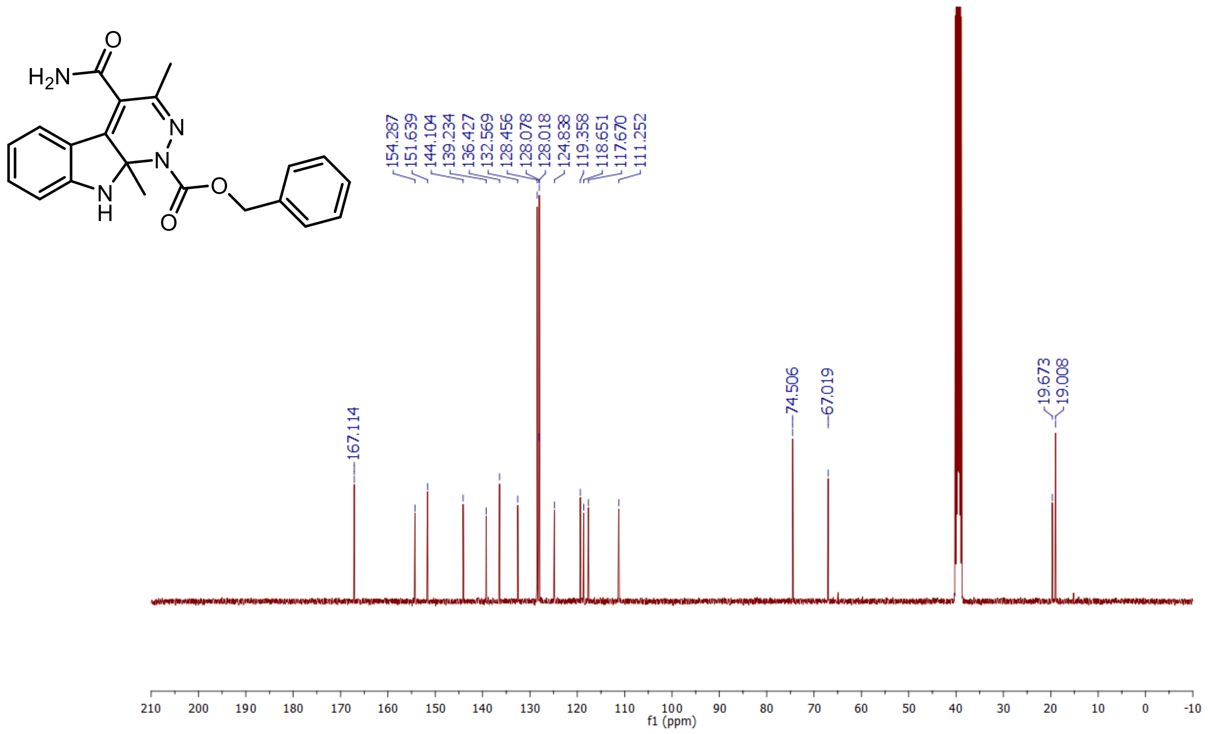

Ethyl 4-(dimethoxyphosphoryl)-3,9a-dimethyl-9,9a-dihydro-1*H*-pyridazino[3,4-*b*]indole-1-carboxylate (3n):

^{1}H NMR (400 MHz, DMSO-d_6) of **3n**:

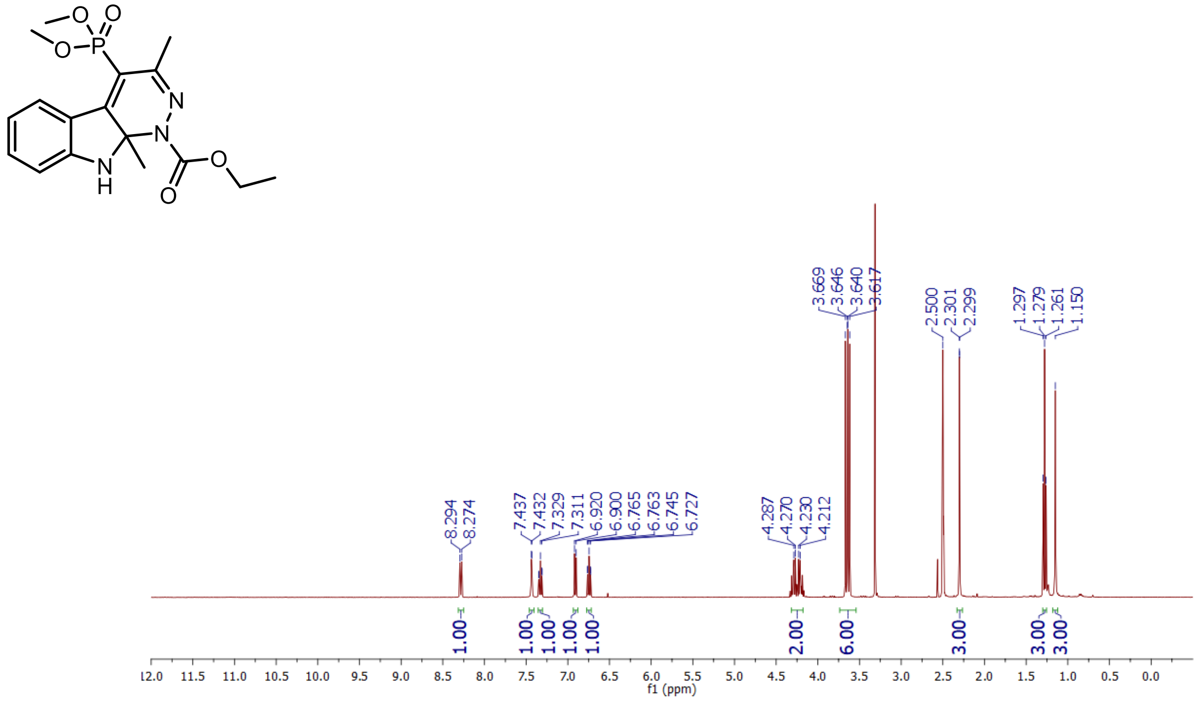

^{13}C{^{1}H} NMR (101 MHz, DMSO-d_6) of **3n**:

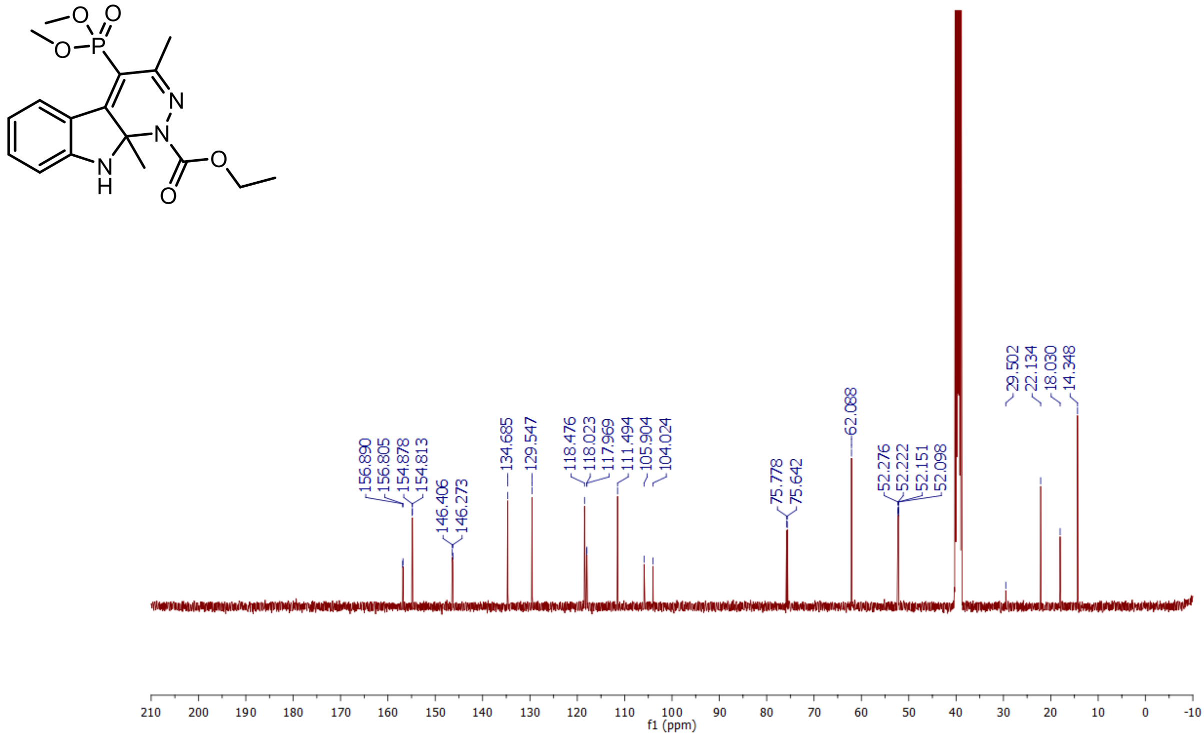

Methyl 3,9a-dimethyl-4-phenyl-9,9a-dihydro-1*H*-pyridazino[3,4-*b*]indole-1-carboxylate (3o):

^{1}H NMR (400 MHz, DMSO-d_6) of **3o**:

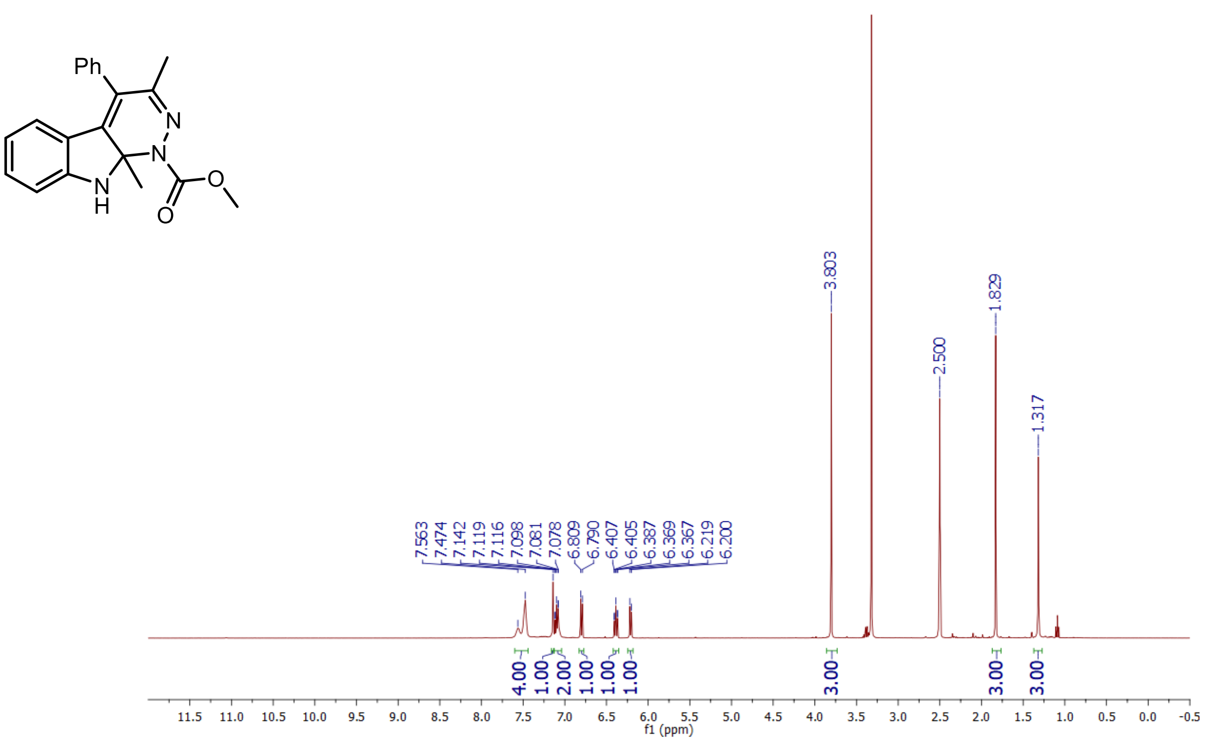

^{13}C{^{1}H} NMR (101 MHz, DMSO-d_6) of **3o**:

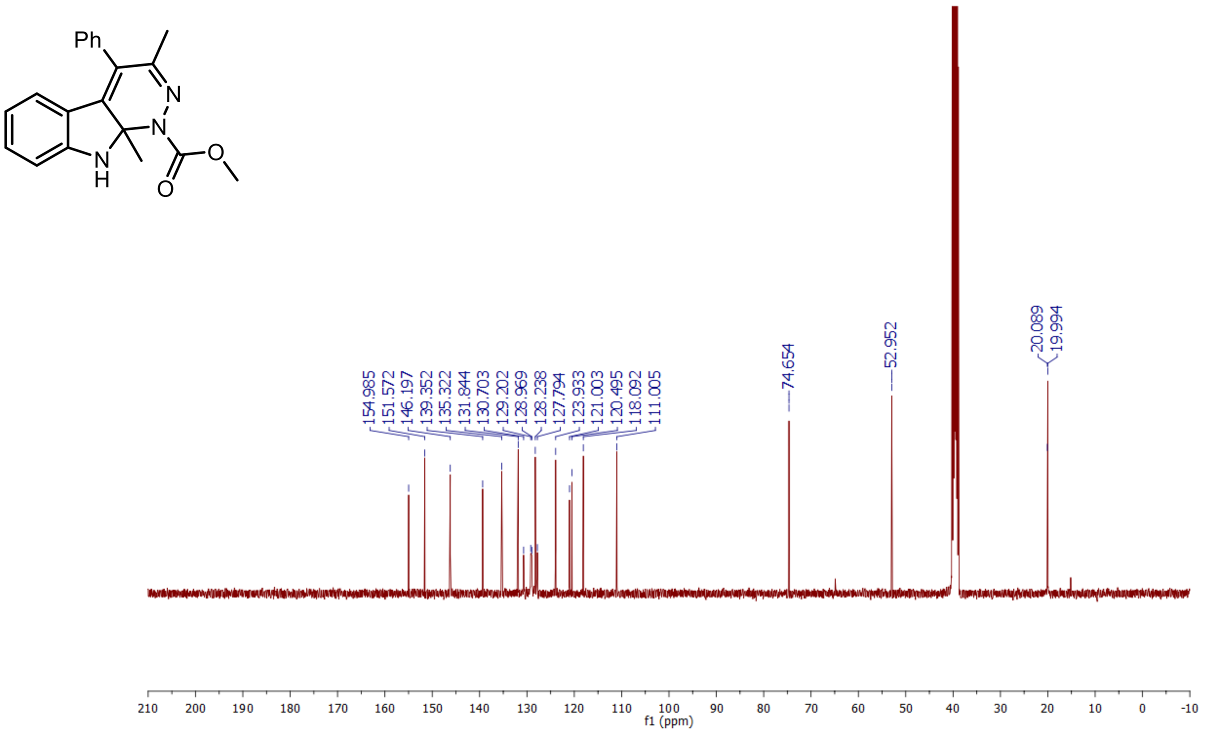

Ethyl 1-carbamoyl-3,9a-dimethyl-9,9a-dihydro-1*H*-pyridazino[3,4-*b*]indole-4-carboxylate (3p):

^{1}H NMR (400 MHz, DMSO-d_6) of **3p**:

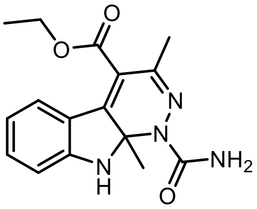

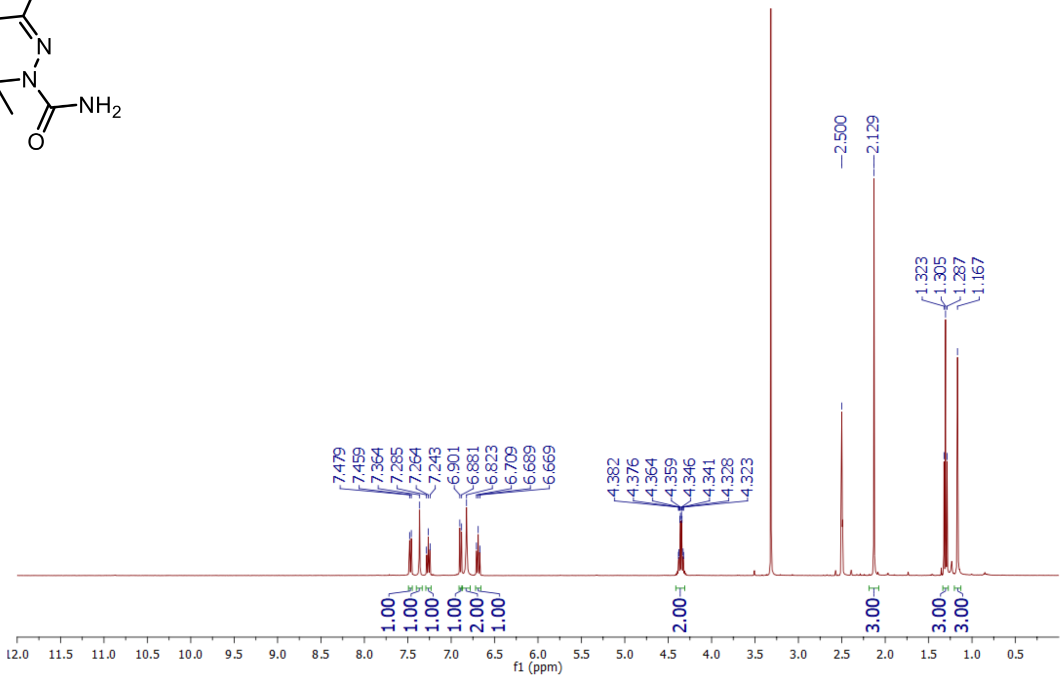

^{13}C{^{1}H} NMR (101 MHz, DMSO-d_6) of **3p**:

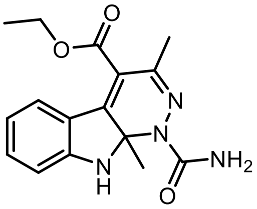

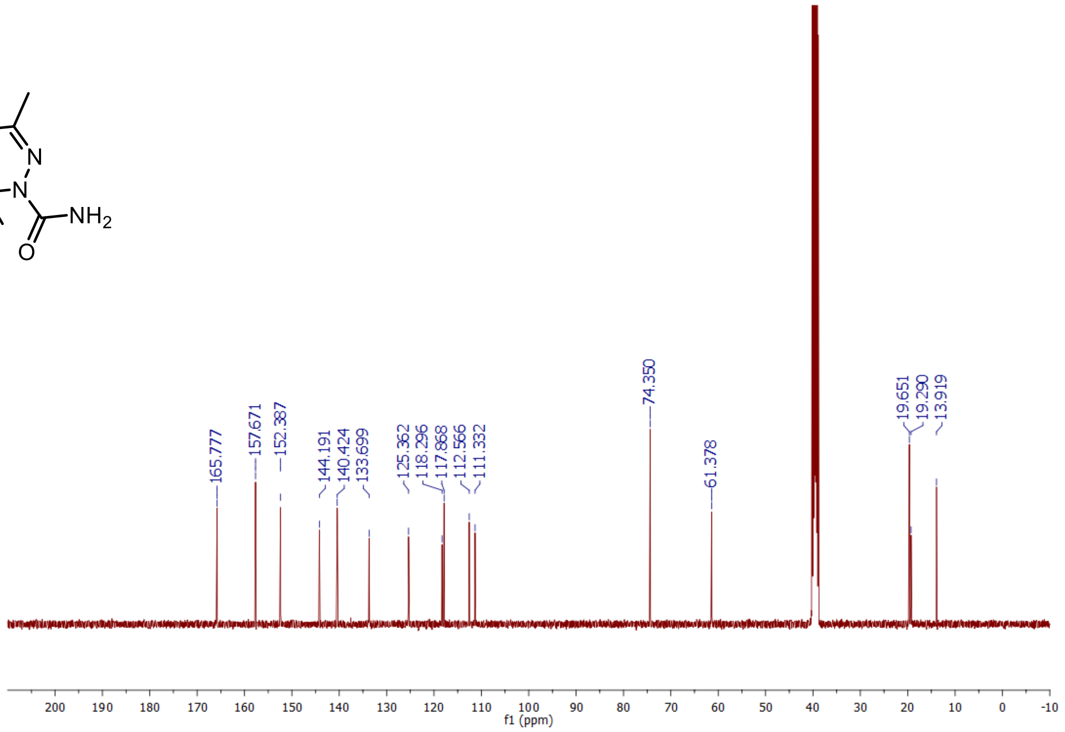

***Tert*-butyl 1-carbamoyl-3,9a-dimethyl-9,9a-dihydro-1*H*-pyridazino[3,4-*b*]indole-4-carboxylate (3q):**

^{1}H NMR (400 MHz, DMSO-d_6) of **3q**:

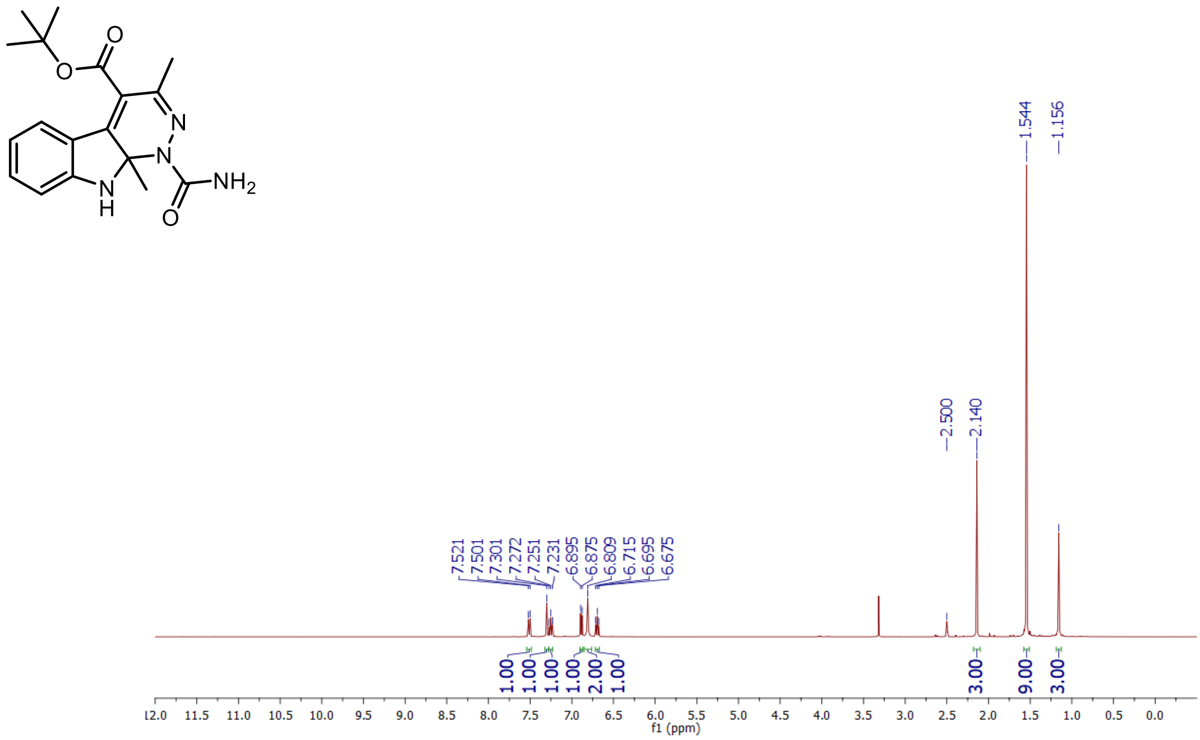

^{13}C{^{1}H} NMR (101 MHz, DMSO-d_6) of **3q**:

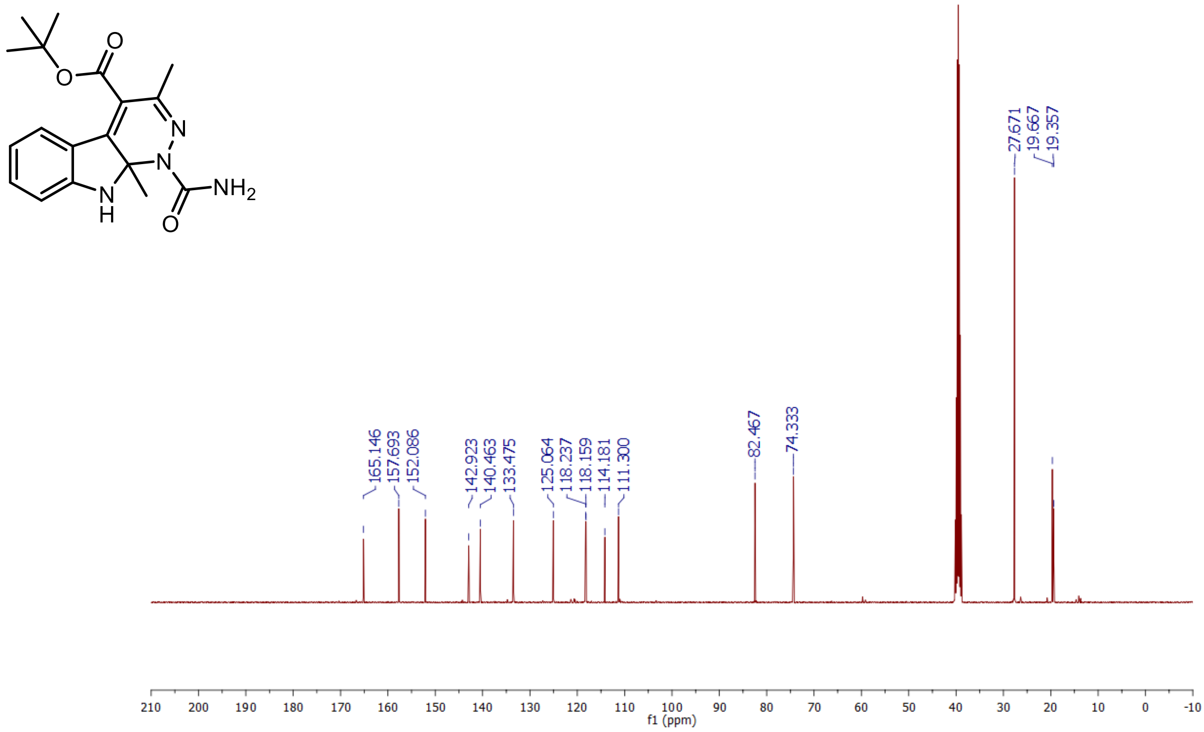

Allyl 1-carbamoyl-3,9a-dimethyl-9,9a-dihydro-1*H*-pyridazino[3,4-*b*]indole-4-carboxylate (3r):
^{1}H NMR (400 MHz, DMSO-d_6) of **3r**:

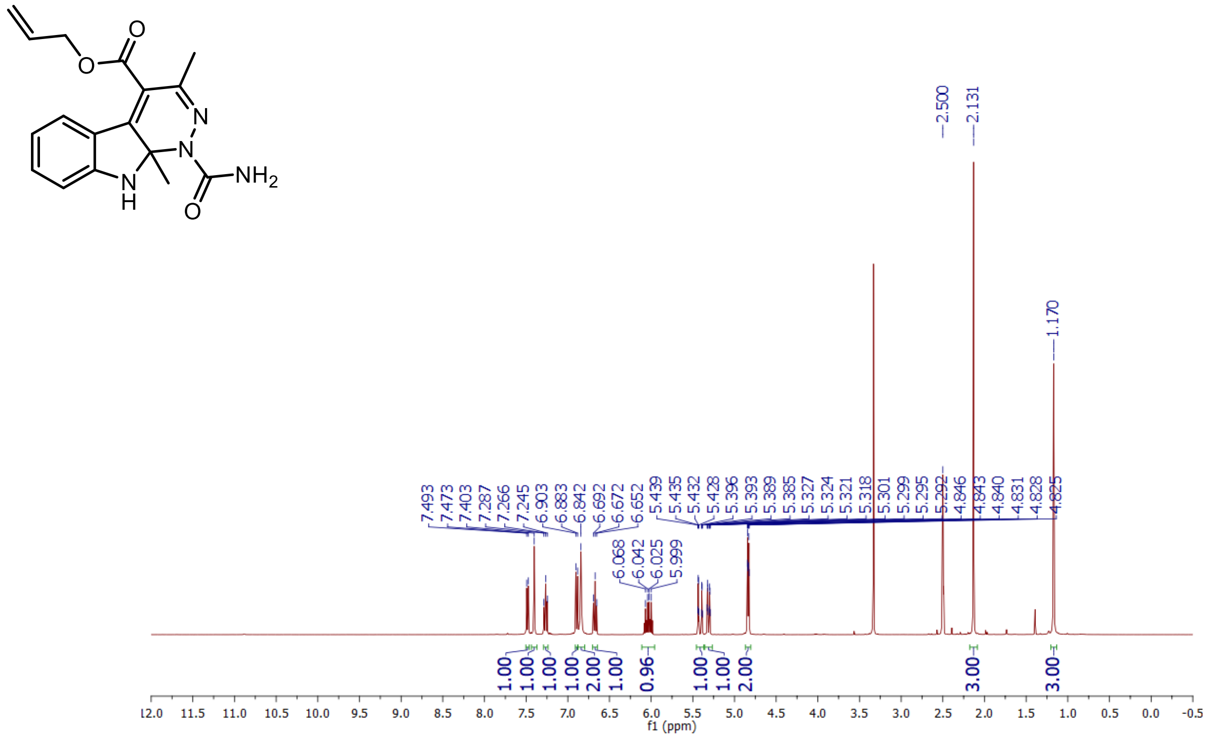

^{13}C{^{1}H} NMR (101 MHz, DMSO-d_6) of **3r**:

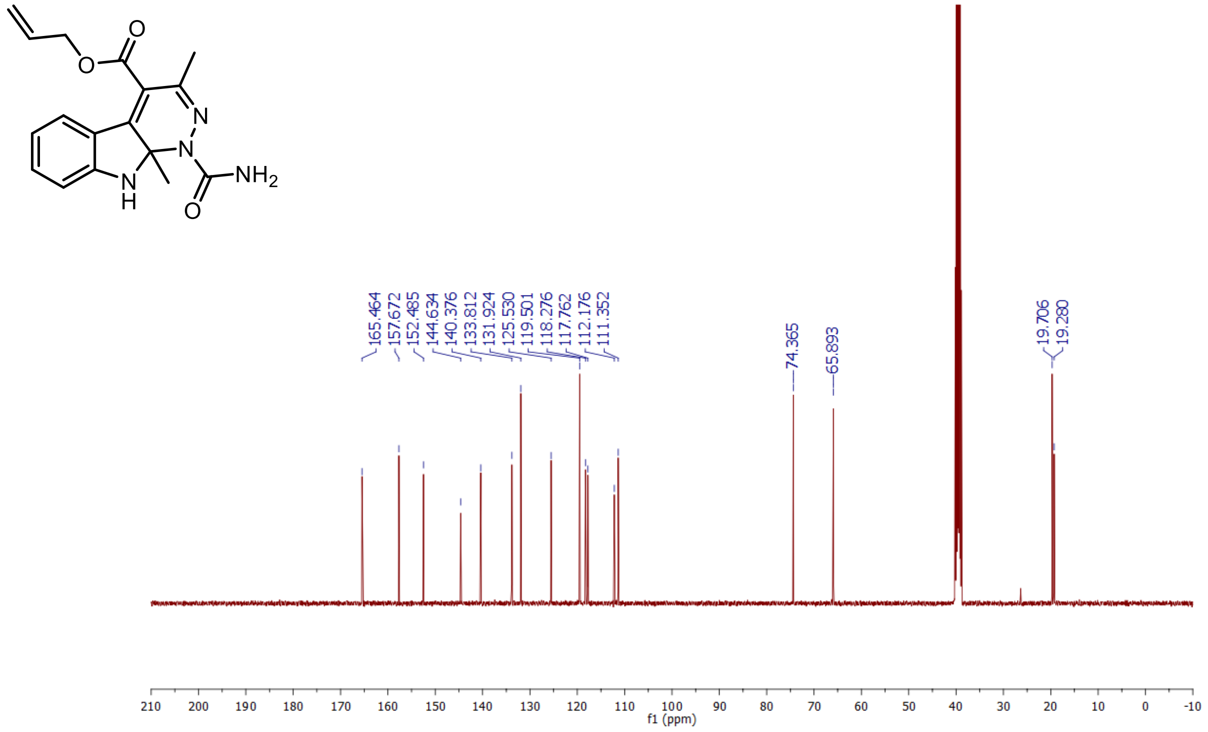

Diethyl 6-chloro-3,9a-dimethyl-9,9a-dihydro-1*H*-pyridazino[3,4-*b*]indole-1,4-dicarboxylate (3s):

^{1}H NMR (400 MHz, DMSO-d_6) of **3s**:

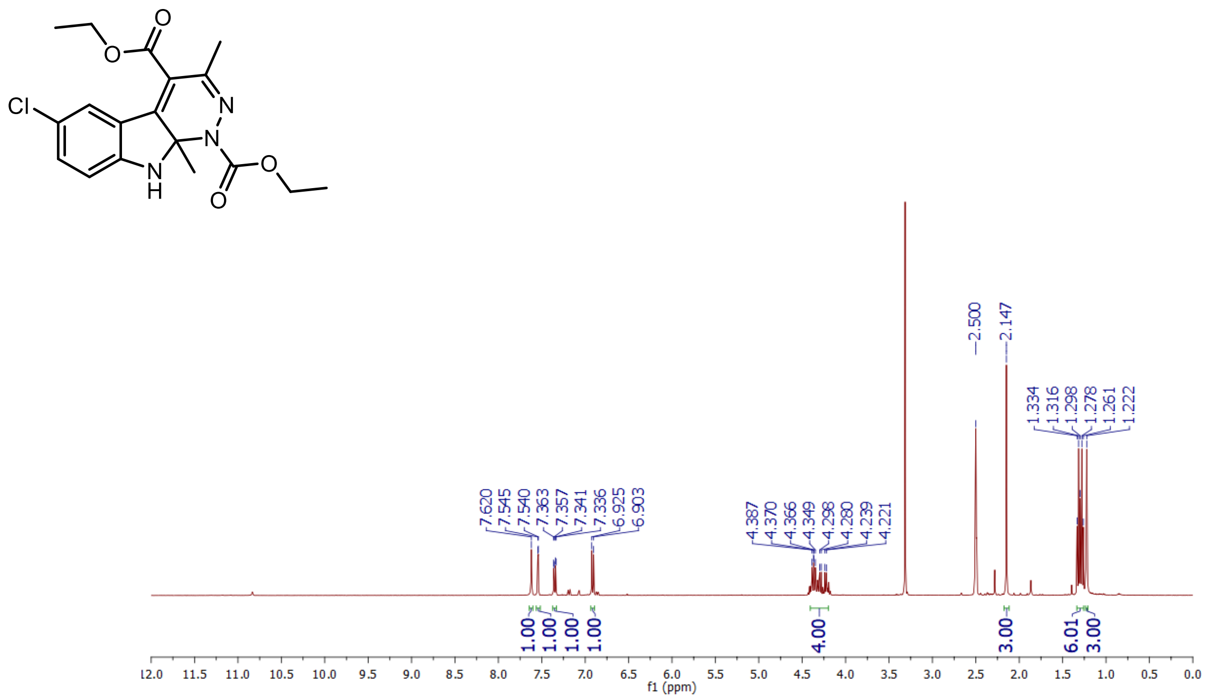

^{13}C{^{1}H} NMR (101 MHz, DMSO-d_6) of **3s**:

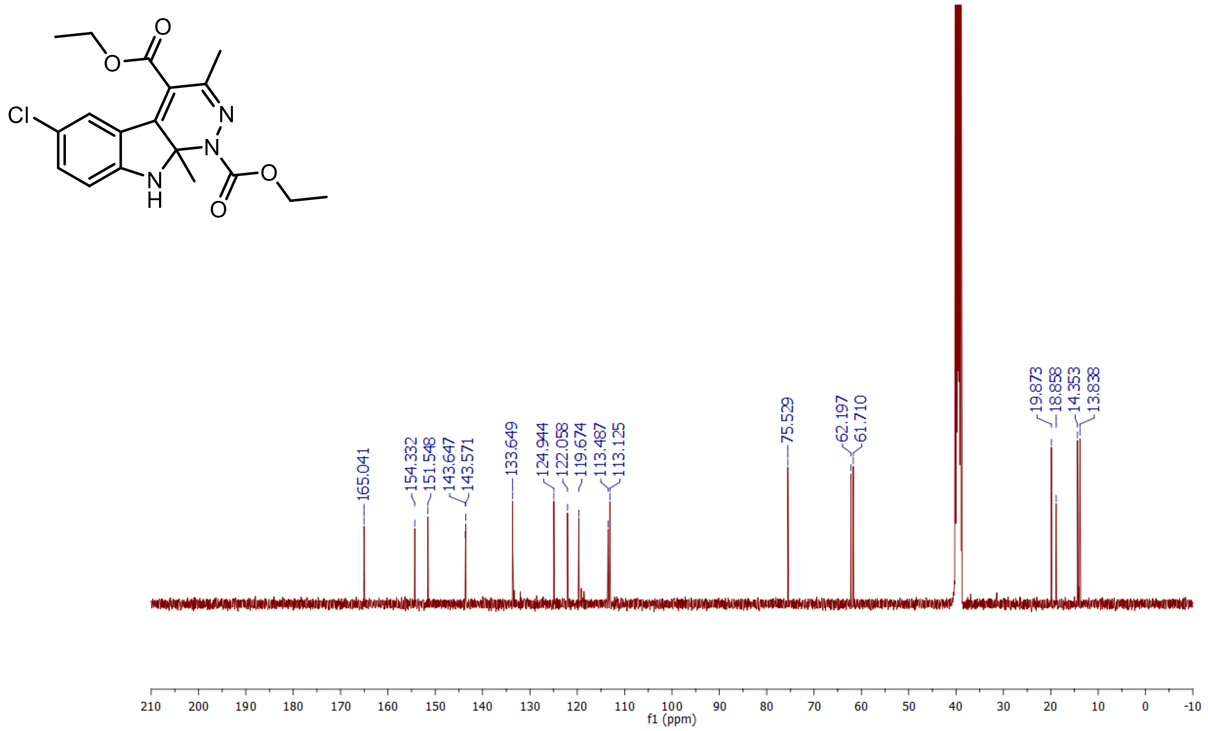

Diethyl 6-fluoro-3,9a-dimethyl-9,9a-dihydro-1*H*-pyridazino[3,4-*b*]indole-1,4-dicarboxylate (3t):

^{1}H NMR (400 MHz, DMSO-d_6) of **3t**:

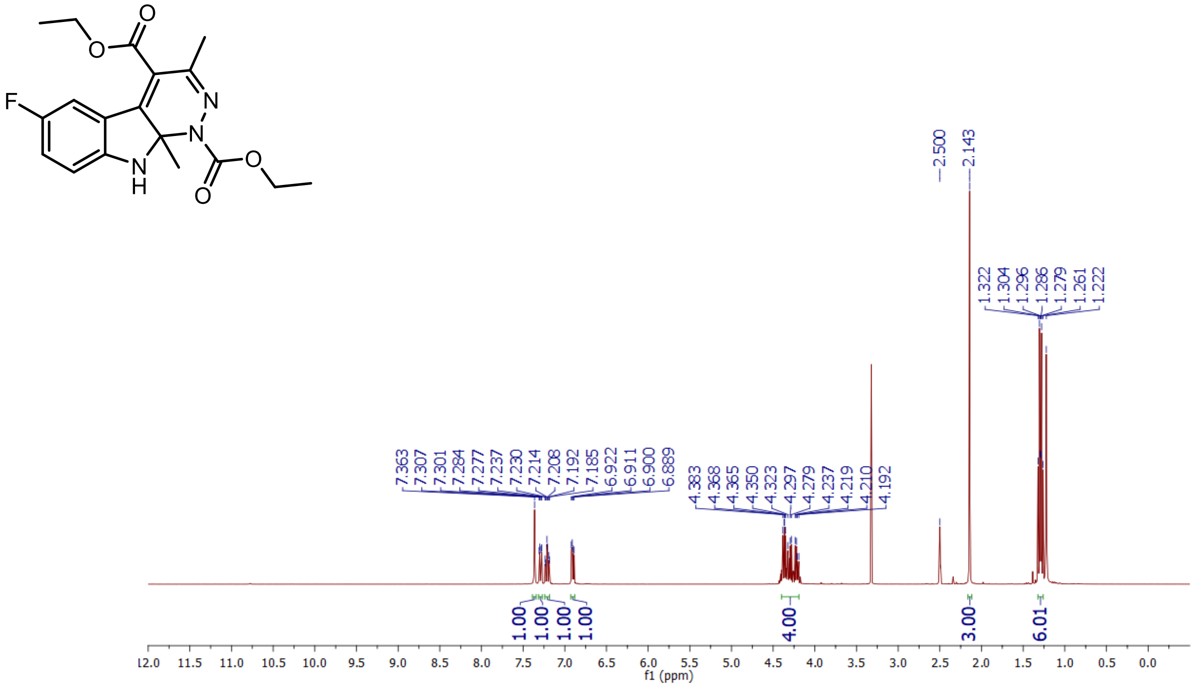

^{13}C{^{1}H} NMR (101 MHz, DMSO-d_6) of **3t**:

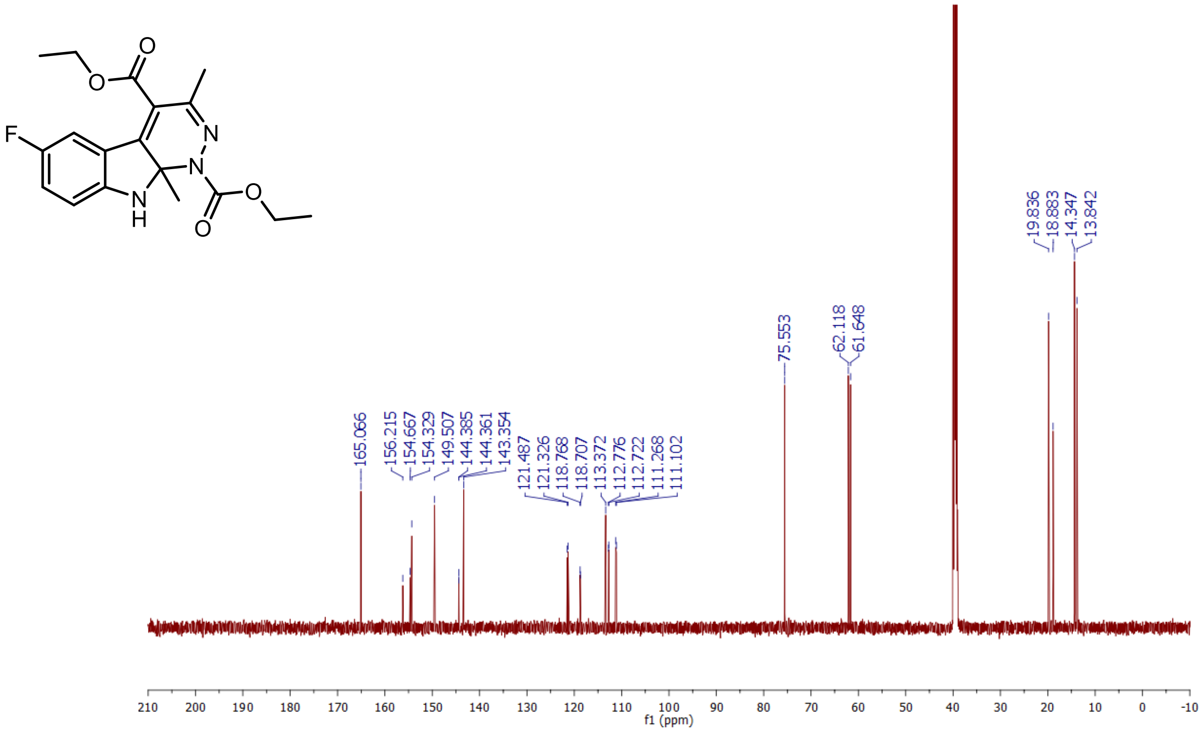

$^{19}F\{^{1}H\}$ NMR (376 MHz, DMSO-d_6) of **3t**:

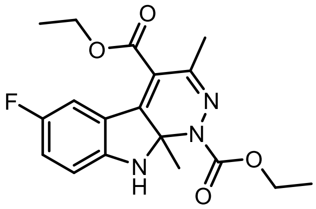

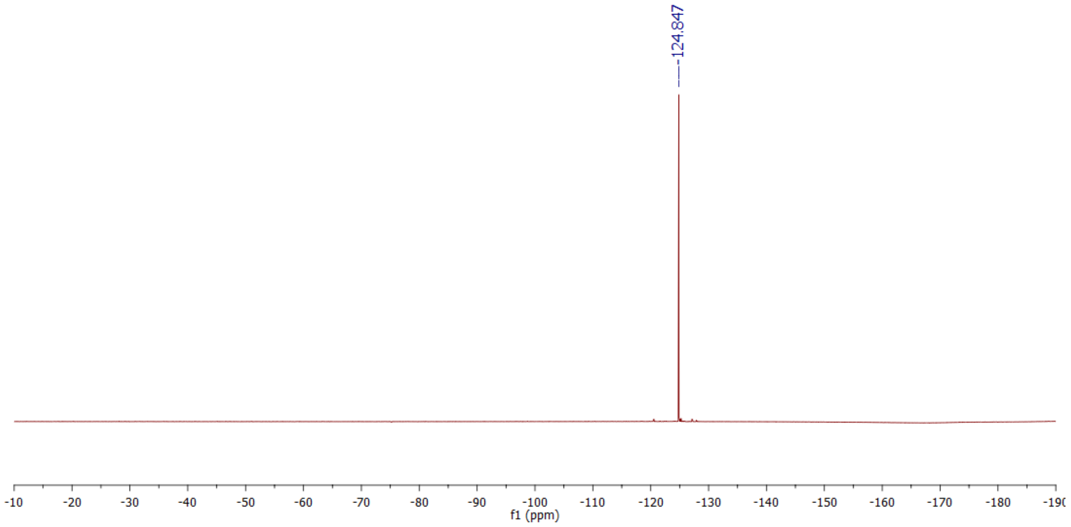

Tert-butyl 1-carbamoyl-7-fluoro-3,9a-dimethyl-9,9a-dihydro-1*H*-pyridazino[3,4-*b*]indole-4-carboxylate (3u):

^{1}H NMR (400 MHz, DMSO-d_6) of **3u**:

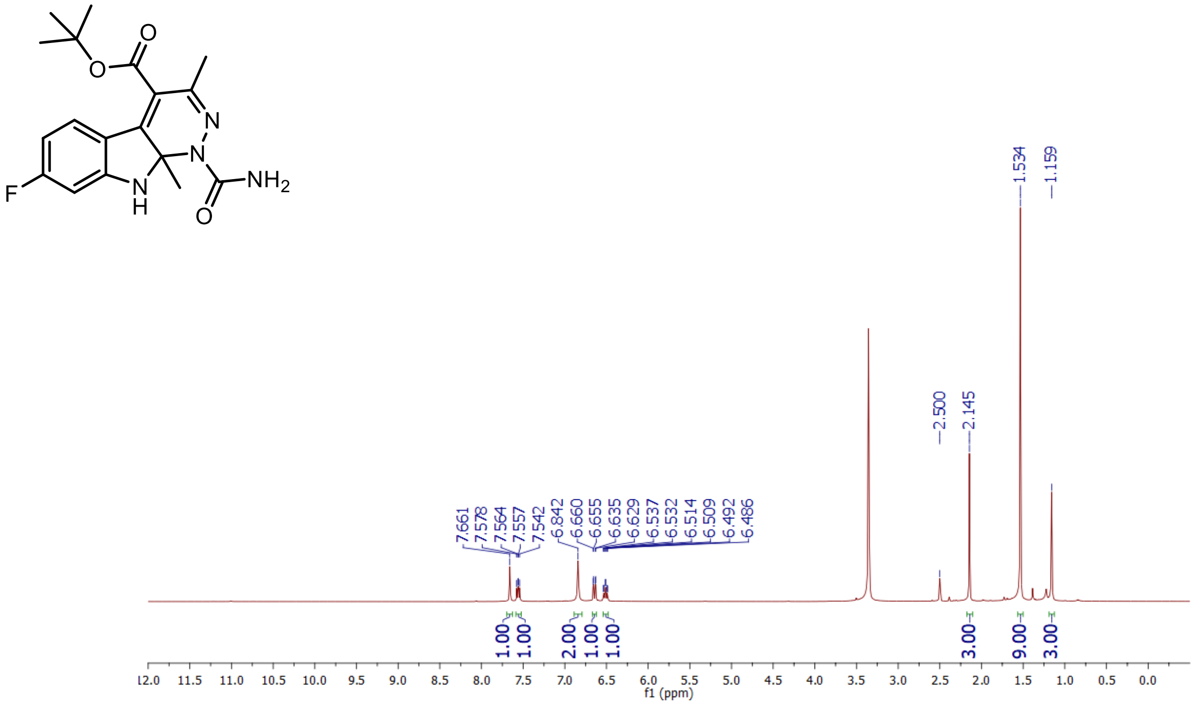

^{13}C{^{1}H} NMR (101 MHz, DMSO-d_6) of **3u**:

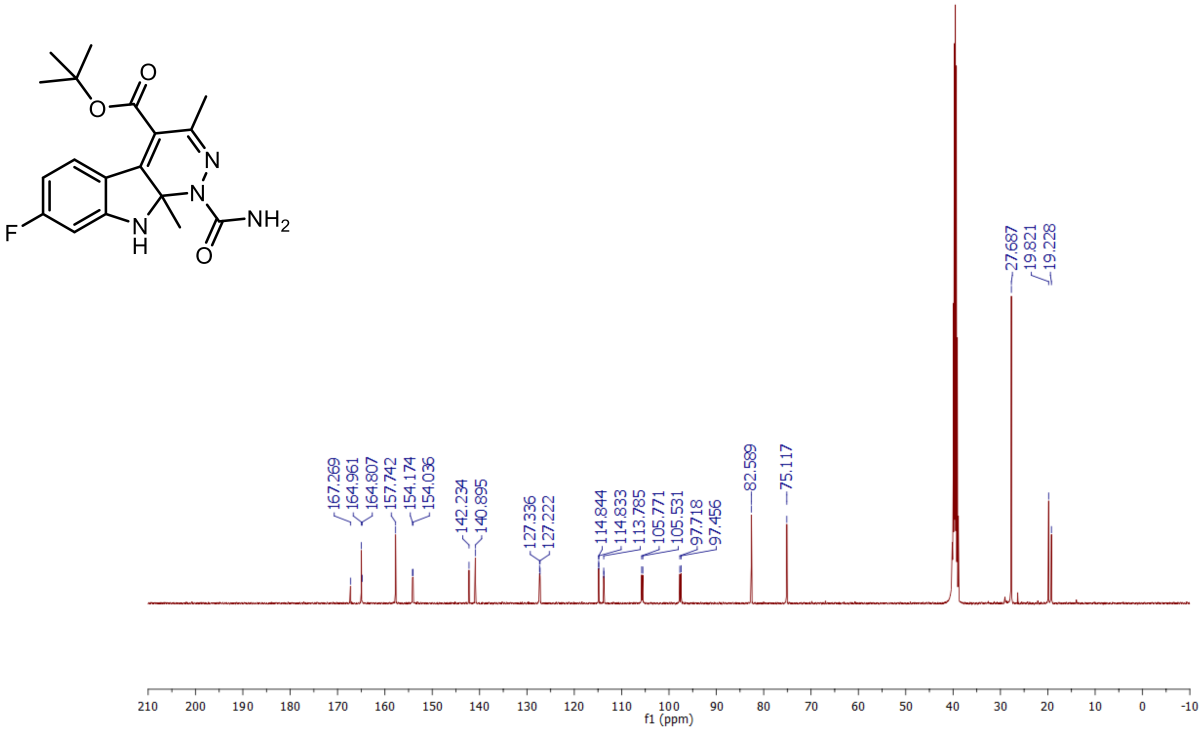

$^{19}F\{^{1}H\}$ NMR (376 MHz, DMSO-d_6) of **3u**:

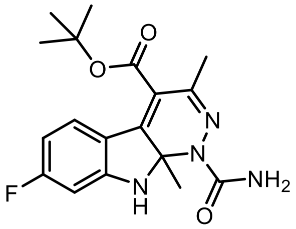

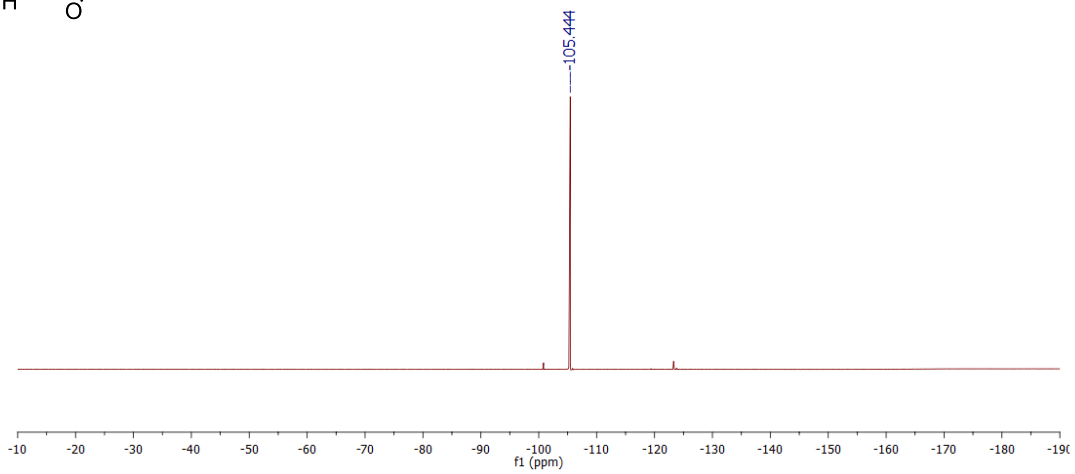

Benzyl 7-bromo-1-carbamoyl-3,9a-dimethyl-9,9a-dihydro-1*H*-pyridazino[3,4-*b*]indole-4-carboxylate (3v):

^{1}H NMR (400 MHz, DMSO-d_6) of **3v**:

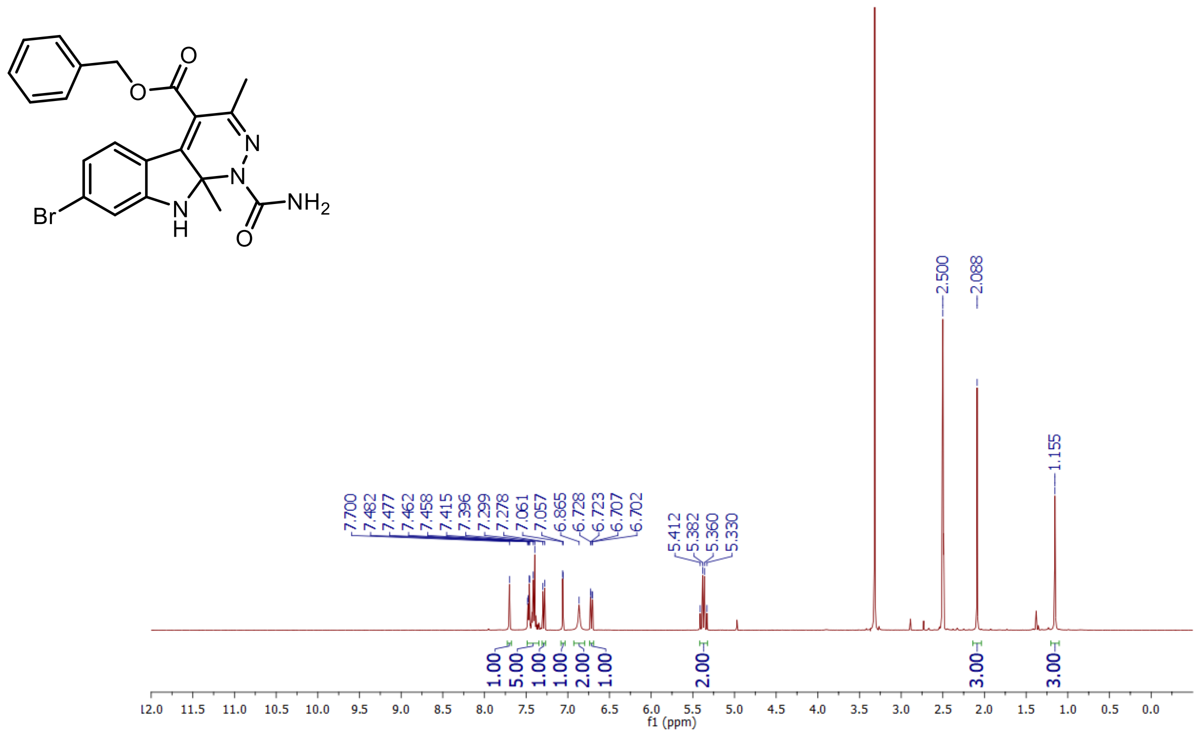

^{13}C{^{1}H} NMR (101 MHz, DMSO-d_6) of **3v**:

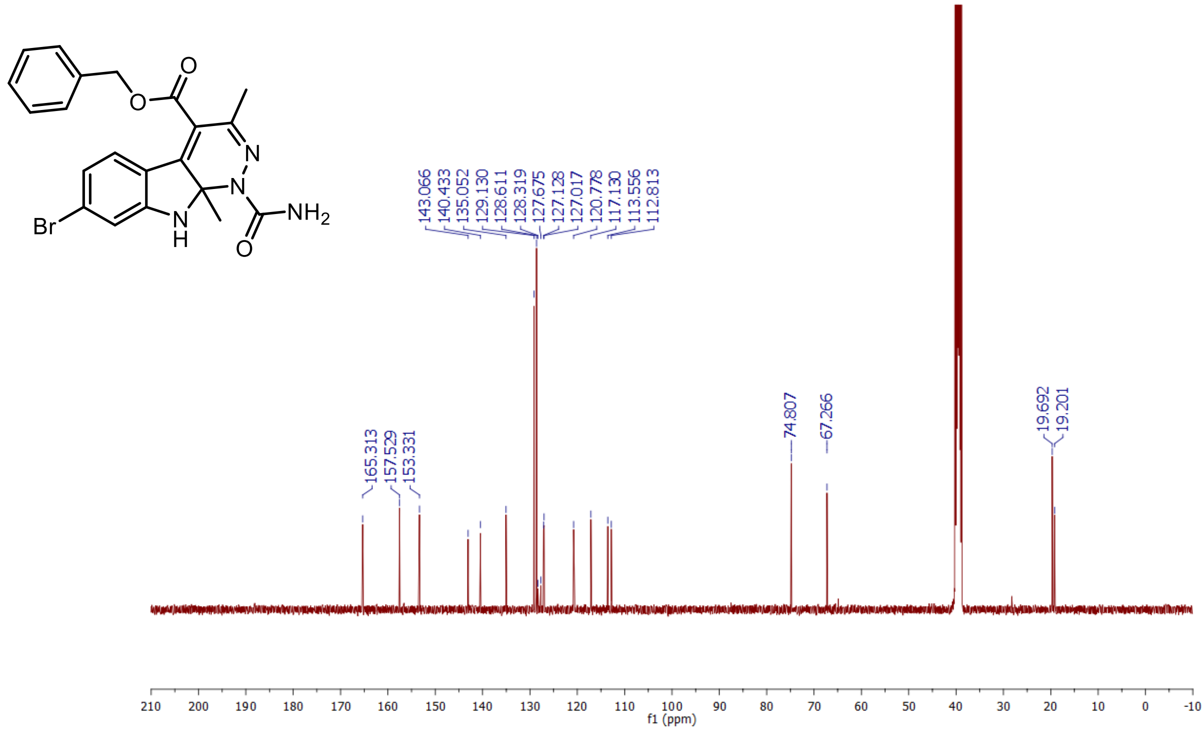

Benzyl 6-bromo-3,9a-dimethyl-1-(phenylcarbamoyl)-9,9a-dihydro-1*H*-pyridazino[3,4-*b*]indole-4-carboxylate (3w):

^{1}H NMR (400 MHz, DMSO-d_6) of **3w**:

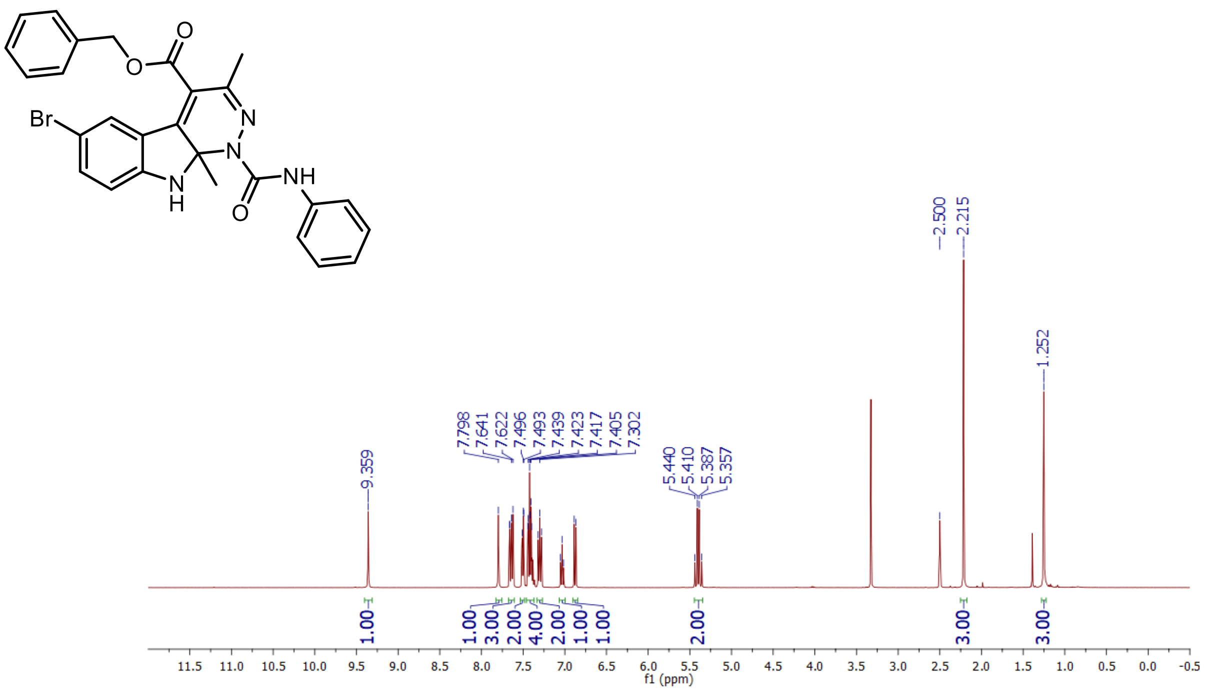

$^{13}\{^{1}H\}$ NMR (101 MHz, DMSO-d_6) of **3w**:

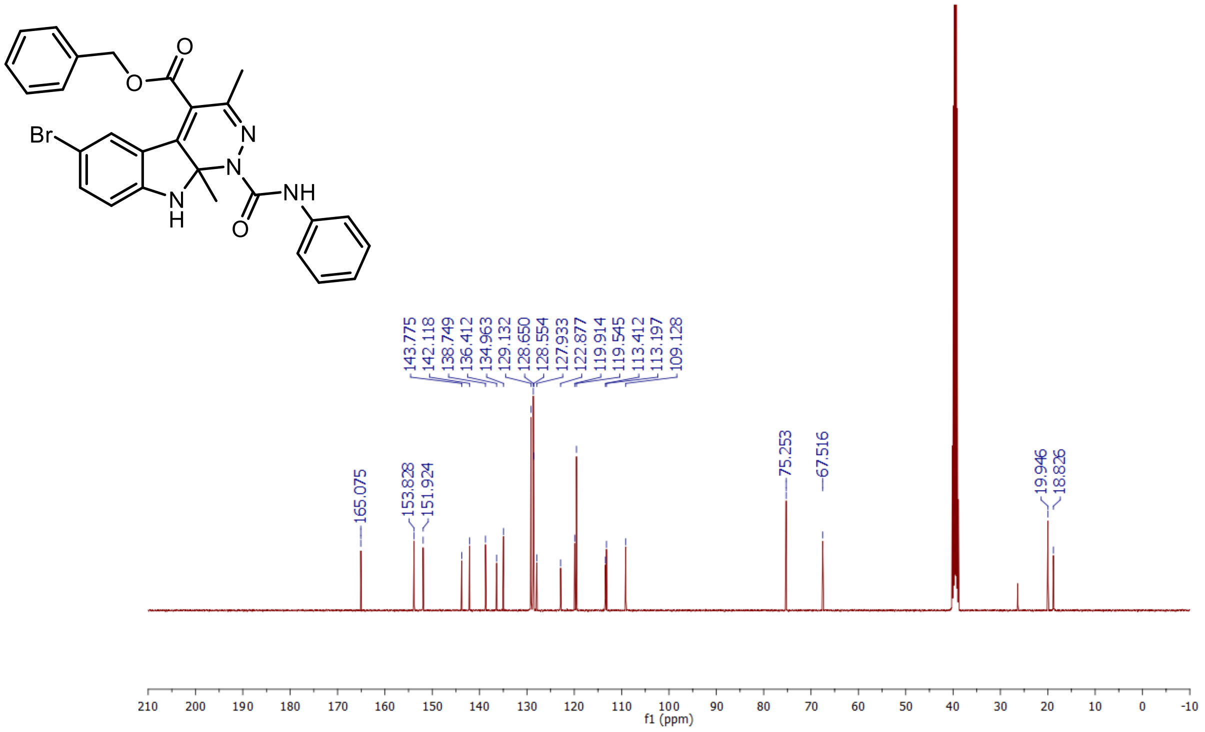

4-Ethyl 1-methyl 3,6,9a-trimethyl-9,9a-dihydro-1*H*-pyridazino[3,4-*b*]indole-1,4-dicarboxylate (3x):

^{1}H NMR (400 MHz, DMSO-d_6) of **3x**:

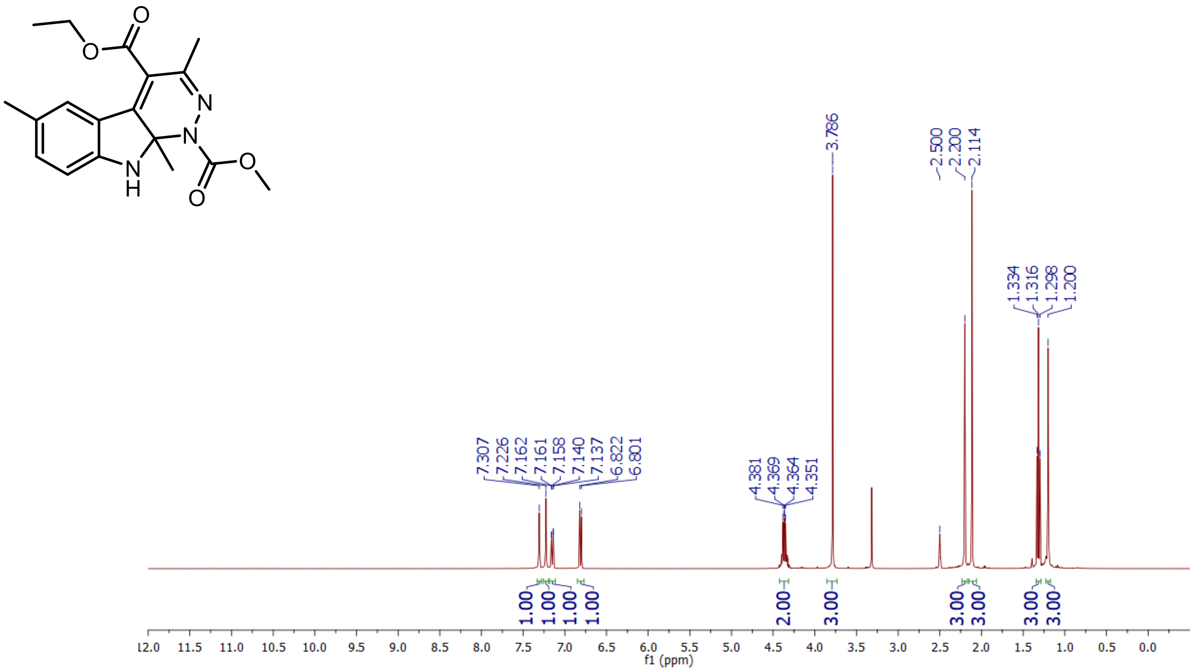

^{13}C{^{1}H} NMR (101 MHz, DMSO-d_6) of **3x**:

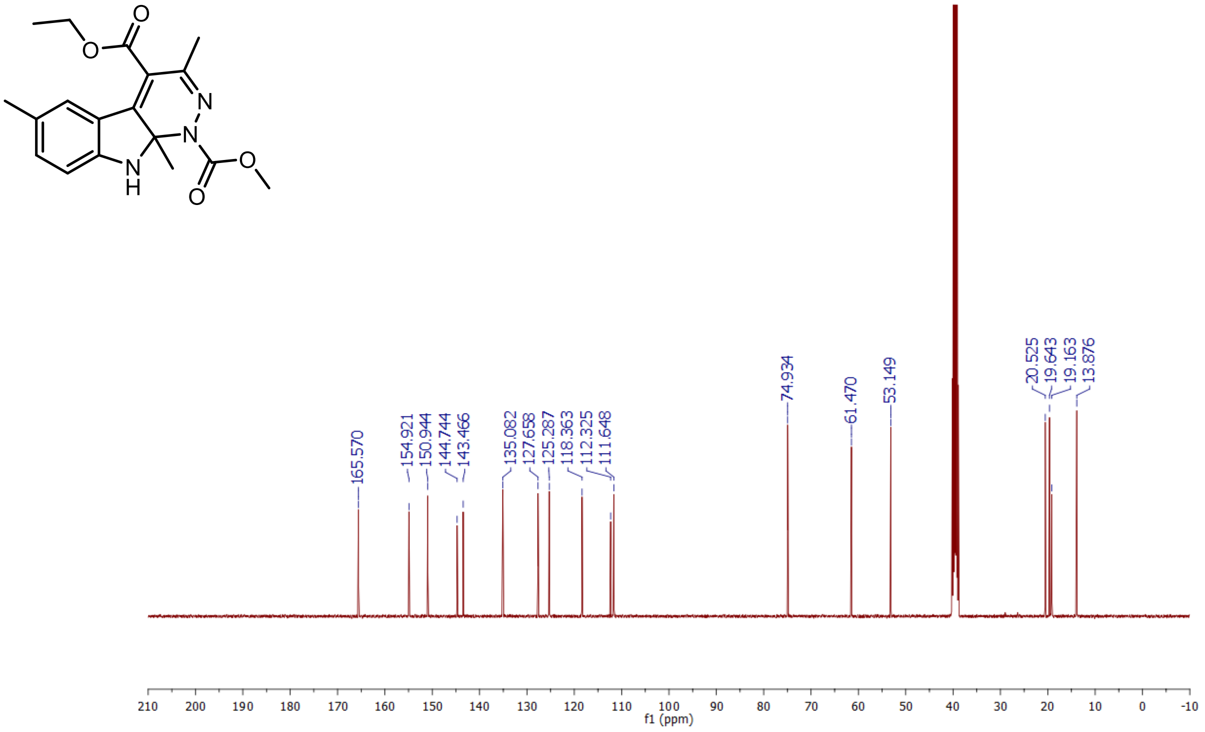

4-Ethyl 1-methyl 3,8,9a-trimethyl-9,9a-dihydro-1H-pyridazino[3,4-b]indole-1,4-dicarboxylate (3y):

^{1}H NMR (400 MHz, DMSO-d_6) of **3y**:

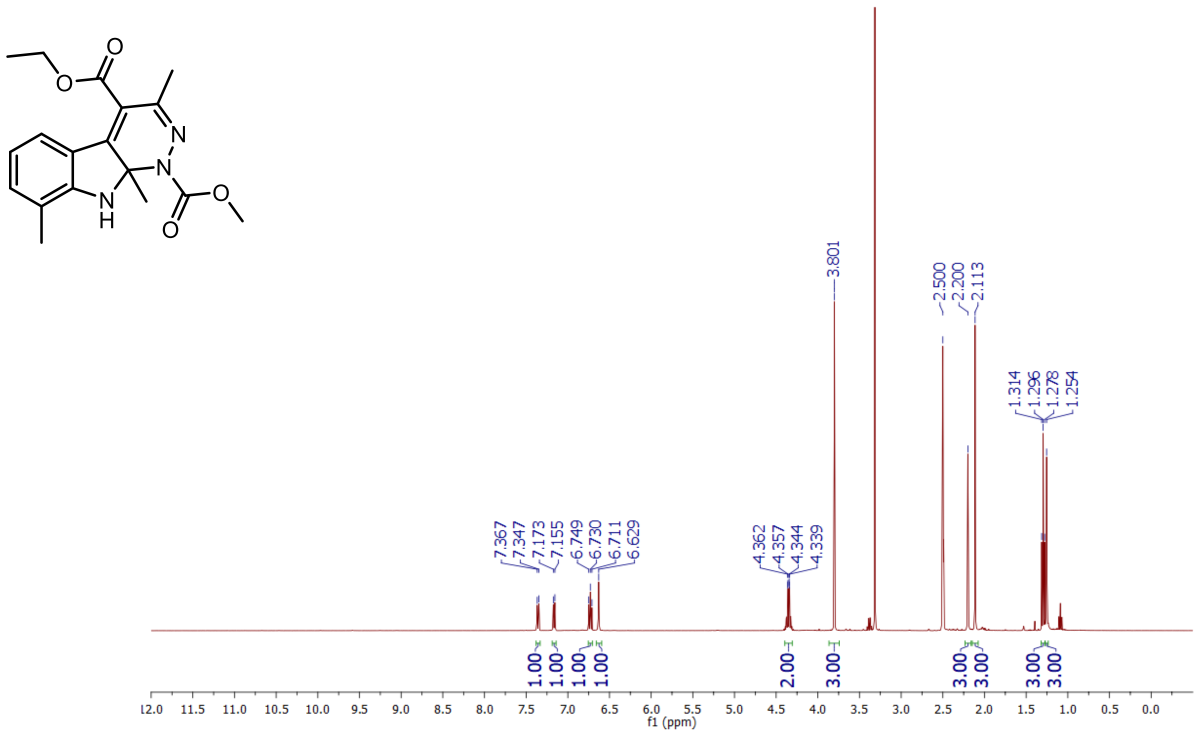

^{13}C{^{1}H} NMR (101 MHz, DMSO-d_6) of **3y**:

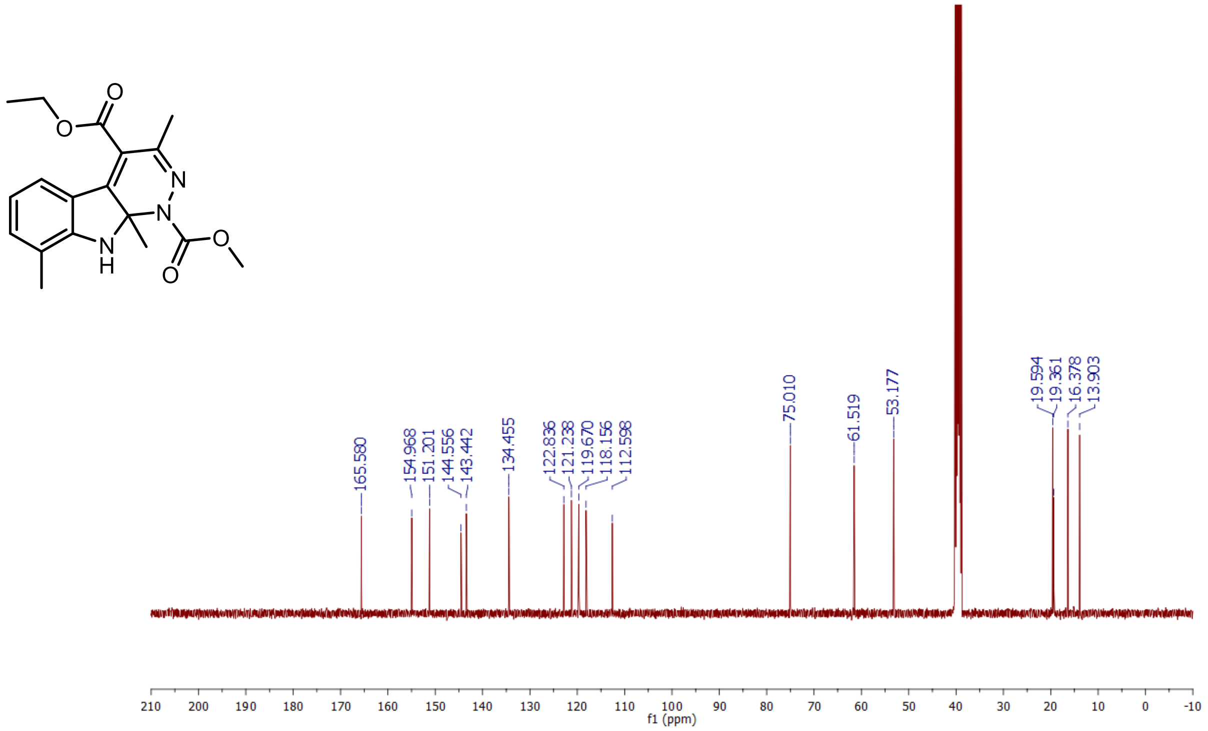

4-Ethyl 1-methyl 6-methoxy-3,9a-dimethyl-9,9a-dihydro-1*H*-pyridazino[3,4-*b*]indole-1,4-dicarboxylate (3z):

^{1}H NMR (400 MHz, DMSO-d_6) of **3z**:

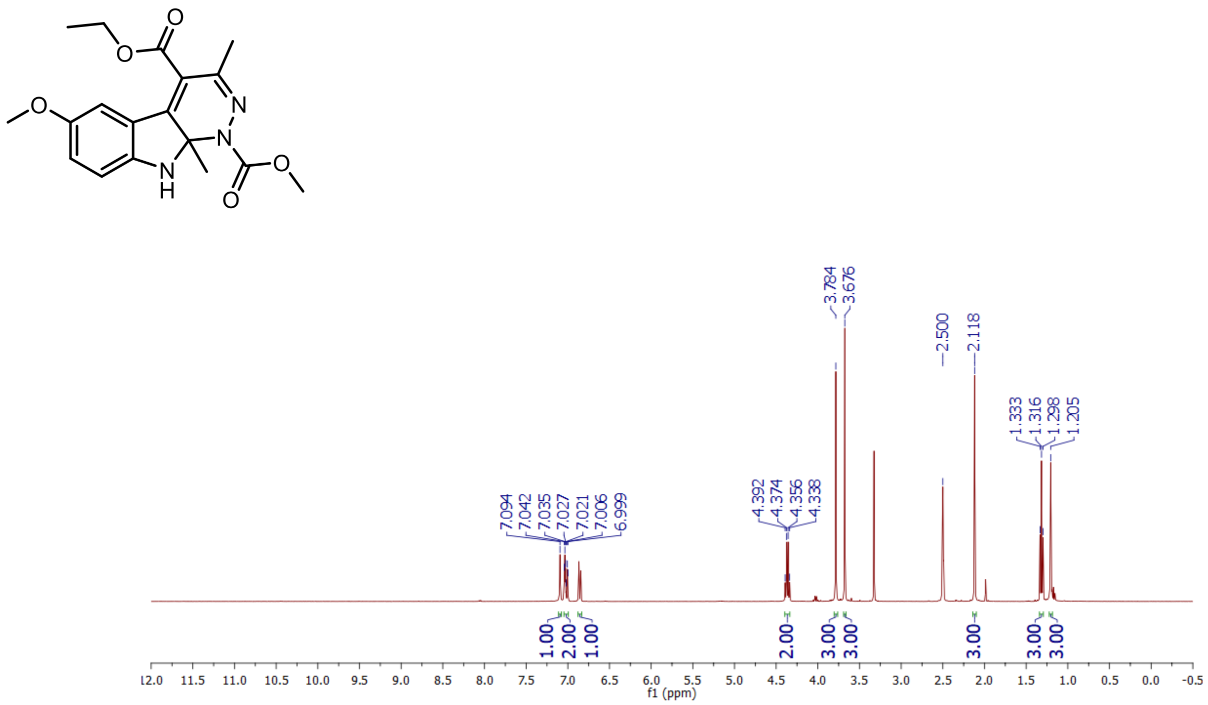

^{13}C{^{1}H} NMR (101 MHz, DMSO-d_6) of **3z**:

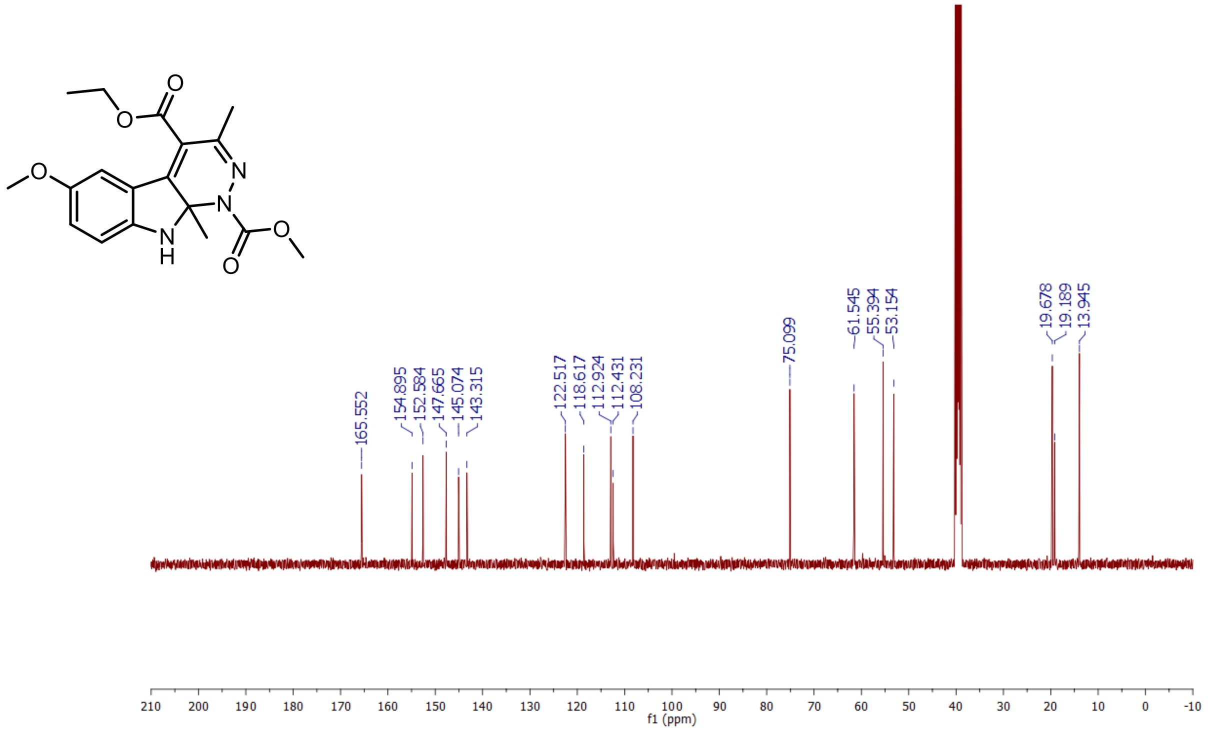

1-Ethyl 4-(2-methoxyethyl) 6-hydroxy-3,9a-dimethyl-9,9a-dihydro-1*H*-pyridazino[3,4-*b*]indole-1,4-dicarboxylate (3za):

^{1}H NMR (400 MHz, DMSO-d_6) of **3za**:

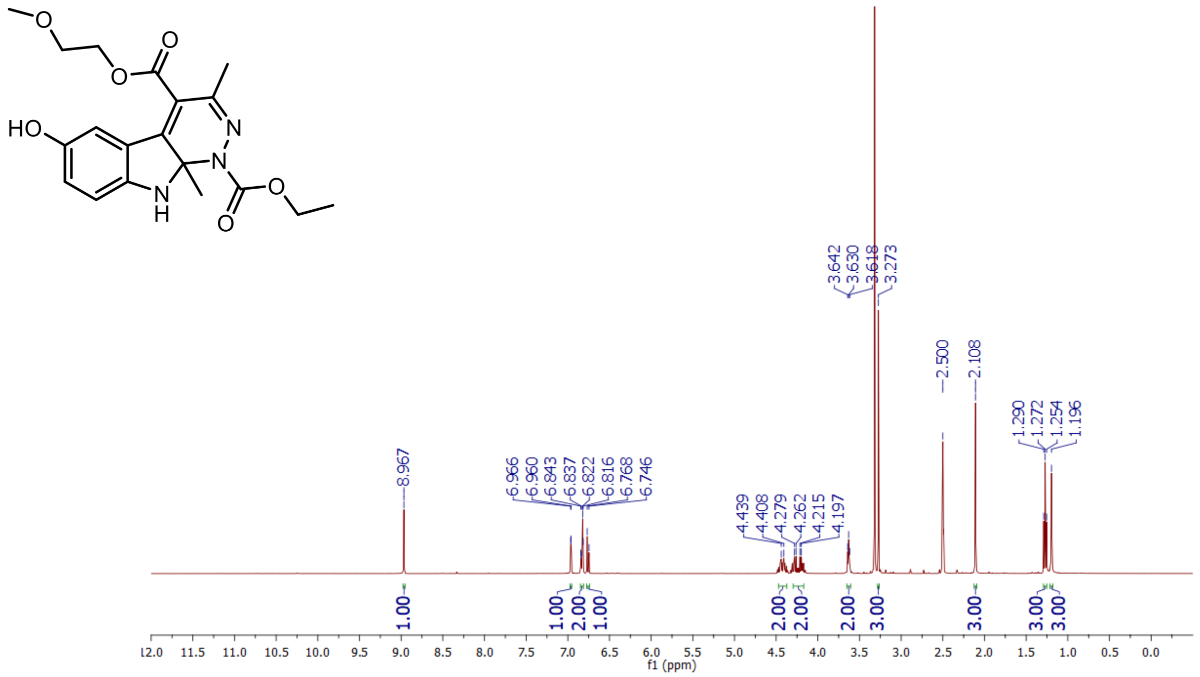

^{13}C{^{1}H} NMR (101 MHz, DMSO-d_6) of **3za**:

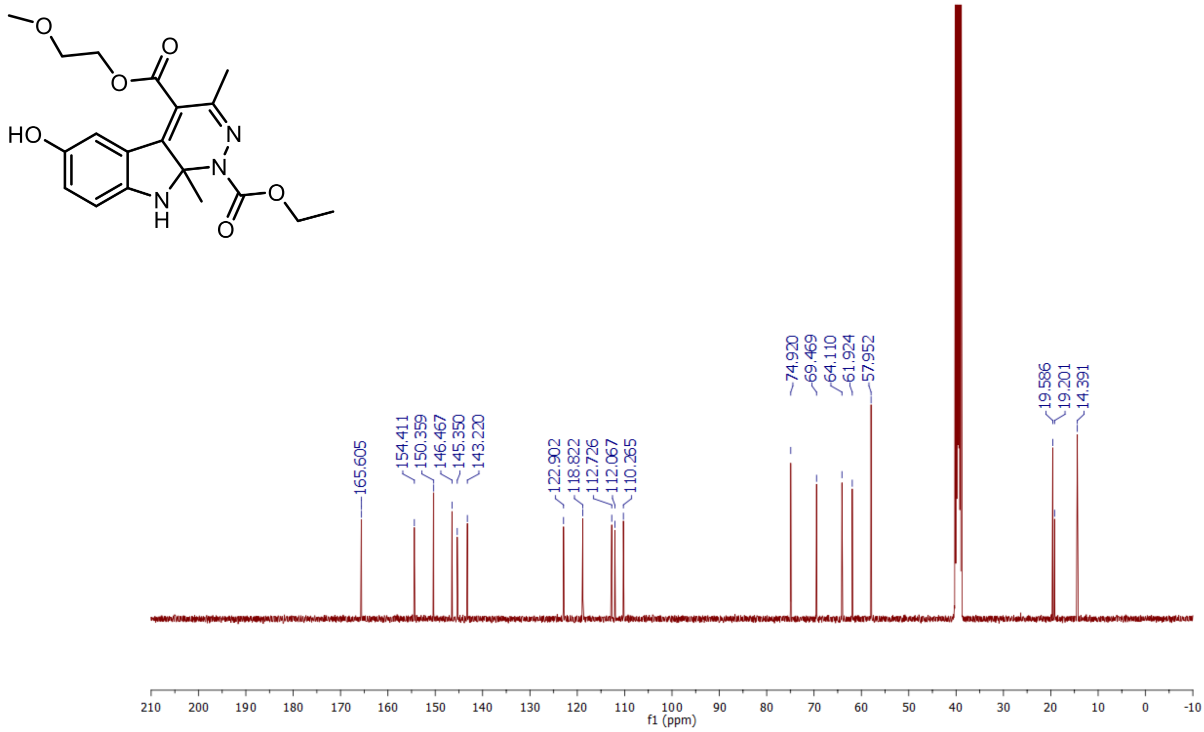

4-Ethyl 1-methyl 9a-ethyl-3-methyl-9,9a-dihydro-1*H*-pyridazino[3,4-*b*]indole-1,4-dicarboxylate (3zb):

^{1}H NMR (400 MHz, DMSO-d_6) of **3zb**:

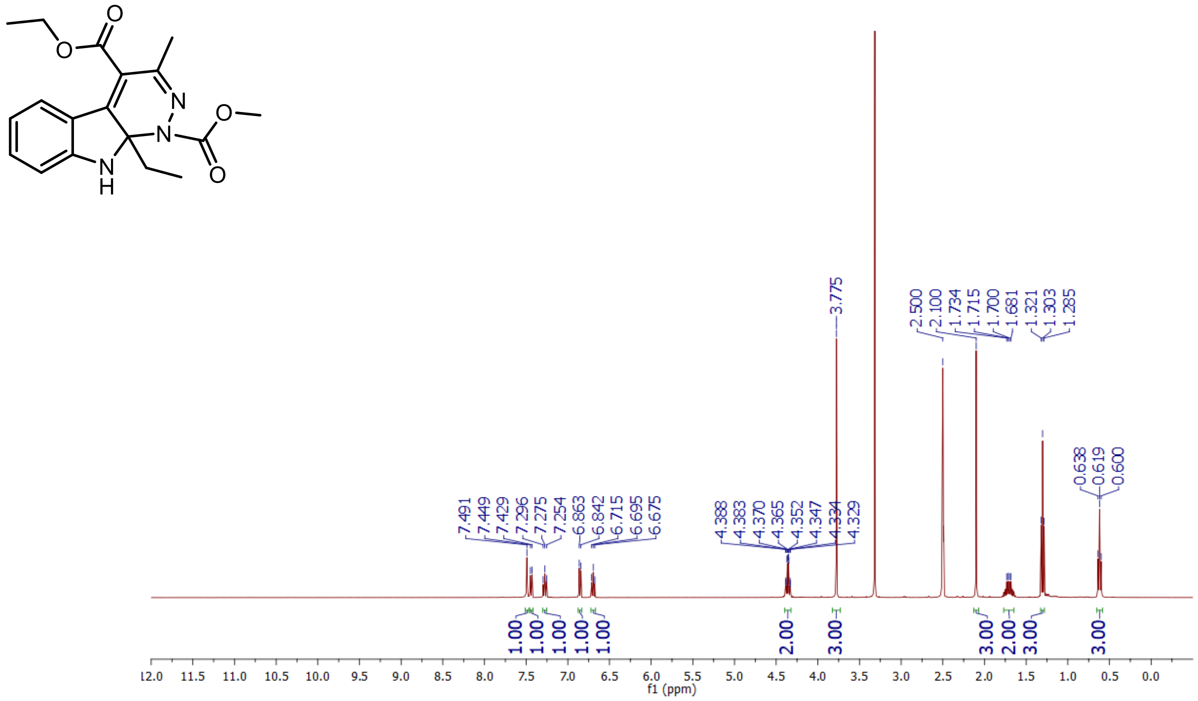

$^{13}C\{^{1}H\}$ NMR (101 MHz, DMSO-d_6) of **3zb**:

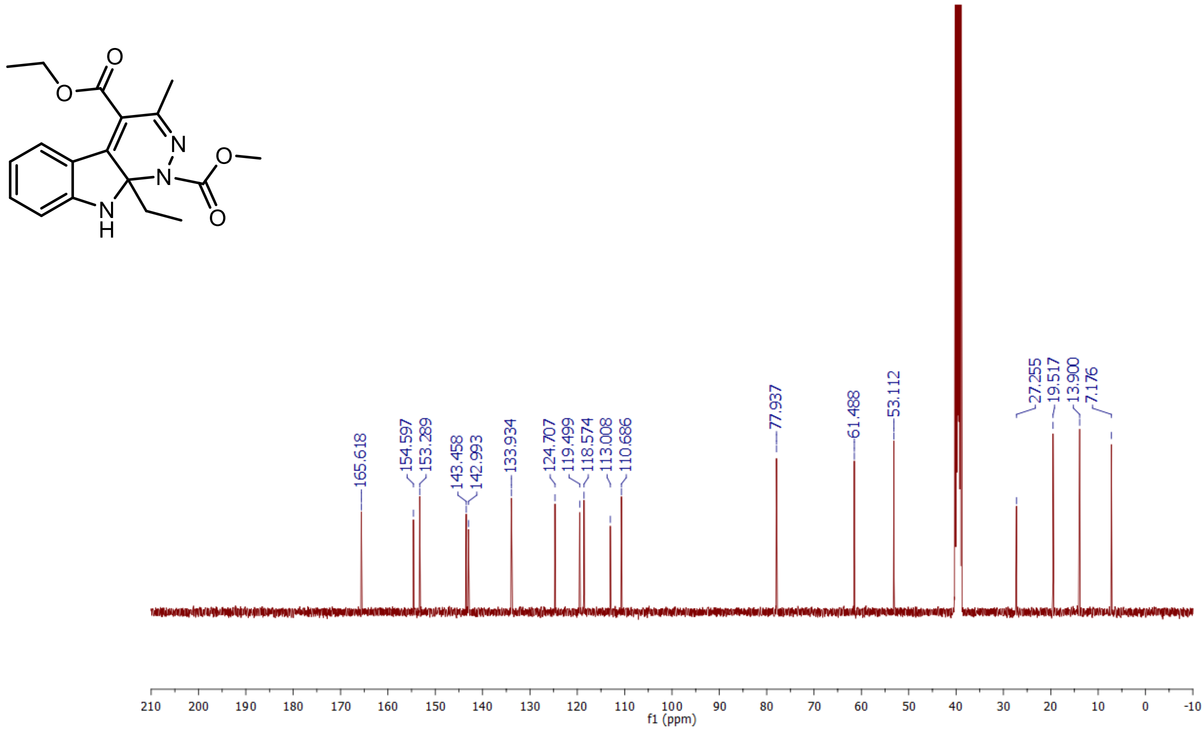

Diethyl 9a-(*tert*-butyl)-3-methyl-9,9a-dihydro-1*H*-pyridazino[3,4-*b*]indole-1,4-dicarboxylate (3zc):

^{1}H NMR (400 MHz, DMSO-d_6) of **3zc**:

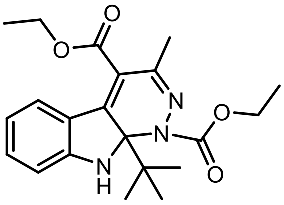

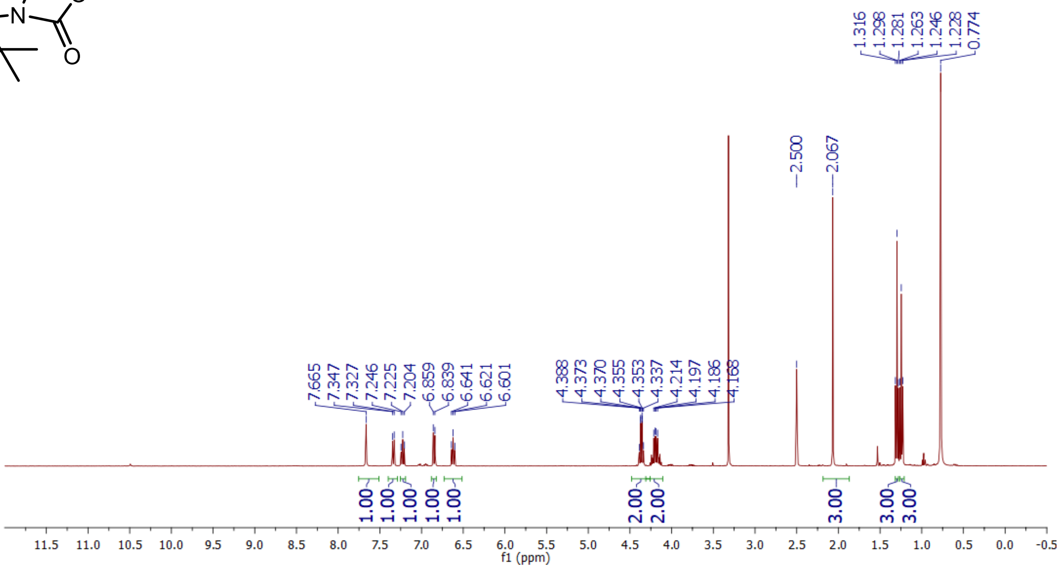

^{13}C{^{1}H} NMR (101 MHz, DMSO-d_6) of **3zc**:

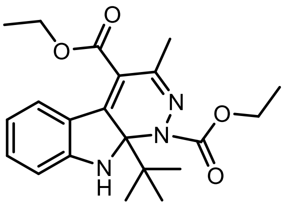

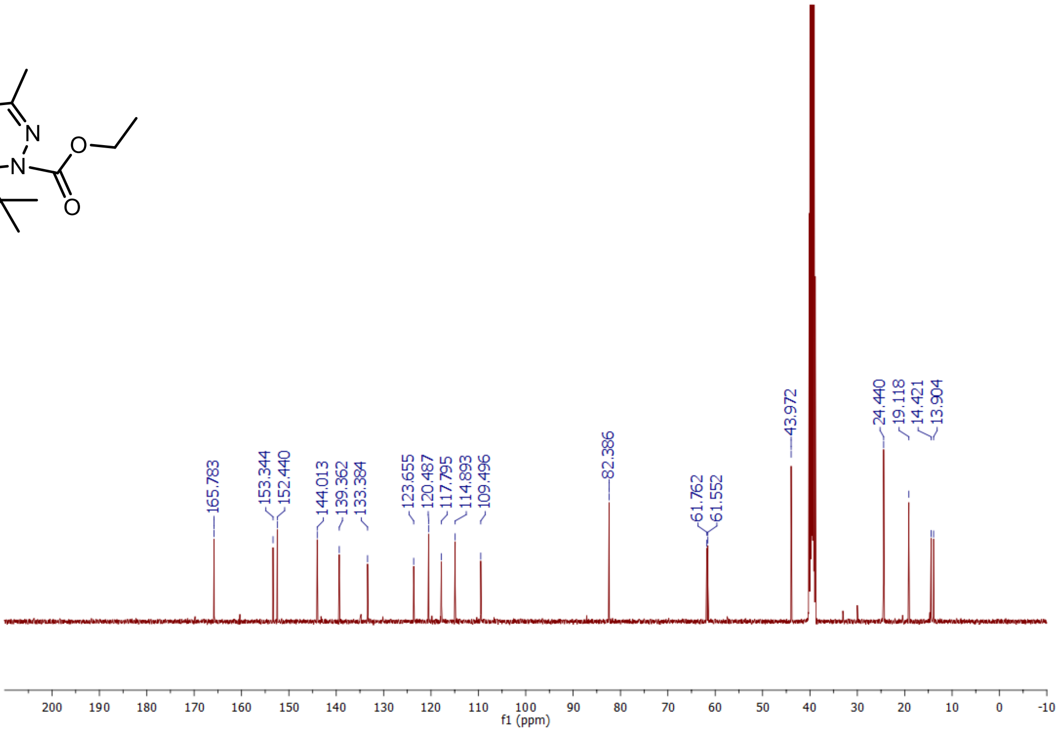

4-Ethyl 1-methyl 3-methyl-9a-phenyl-9,9a-dihydro-1*H*-pyridazino[3,4-*b*]indole-1,4-dicarboxylate (3zd):

^{1}H NMR (400 MHz, DMSO-d_6) of **3zd**:

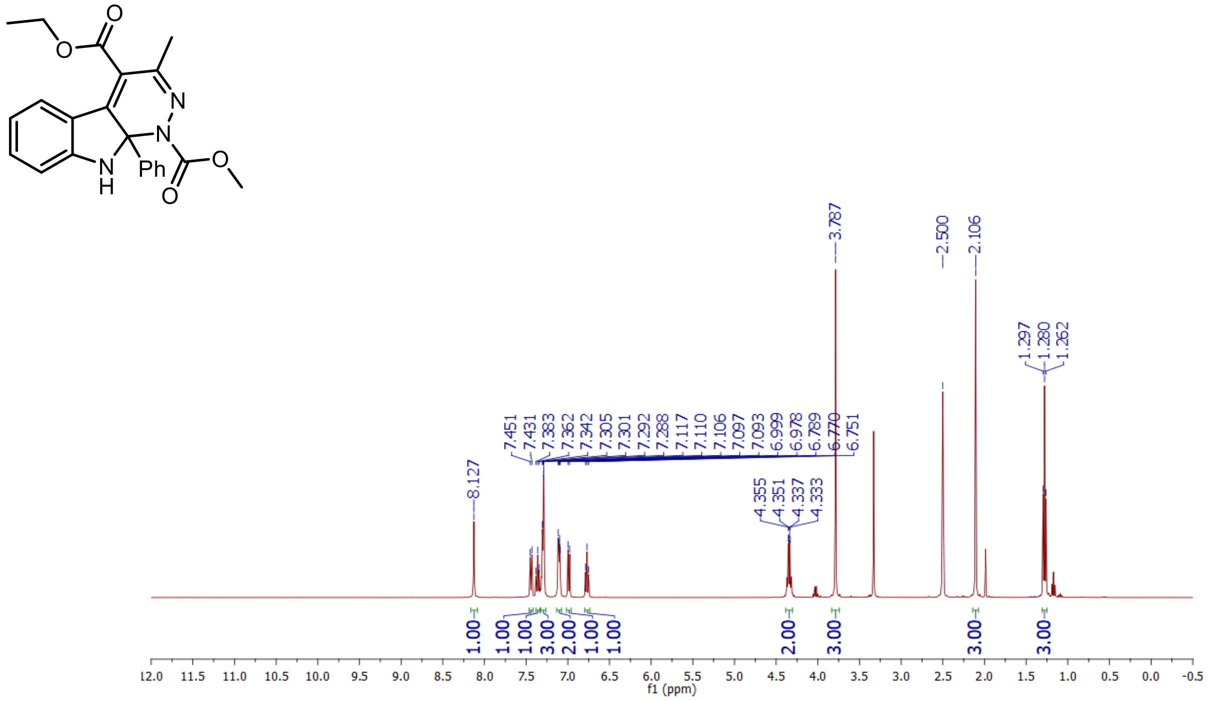

^{13}C{^{1}H} NMR (101 MHz, DMSO-d_6) of **3zd**:

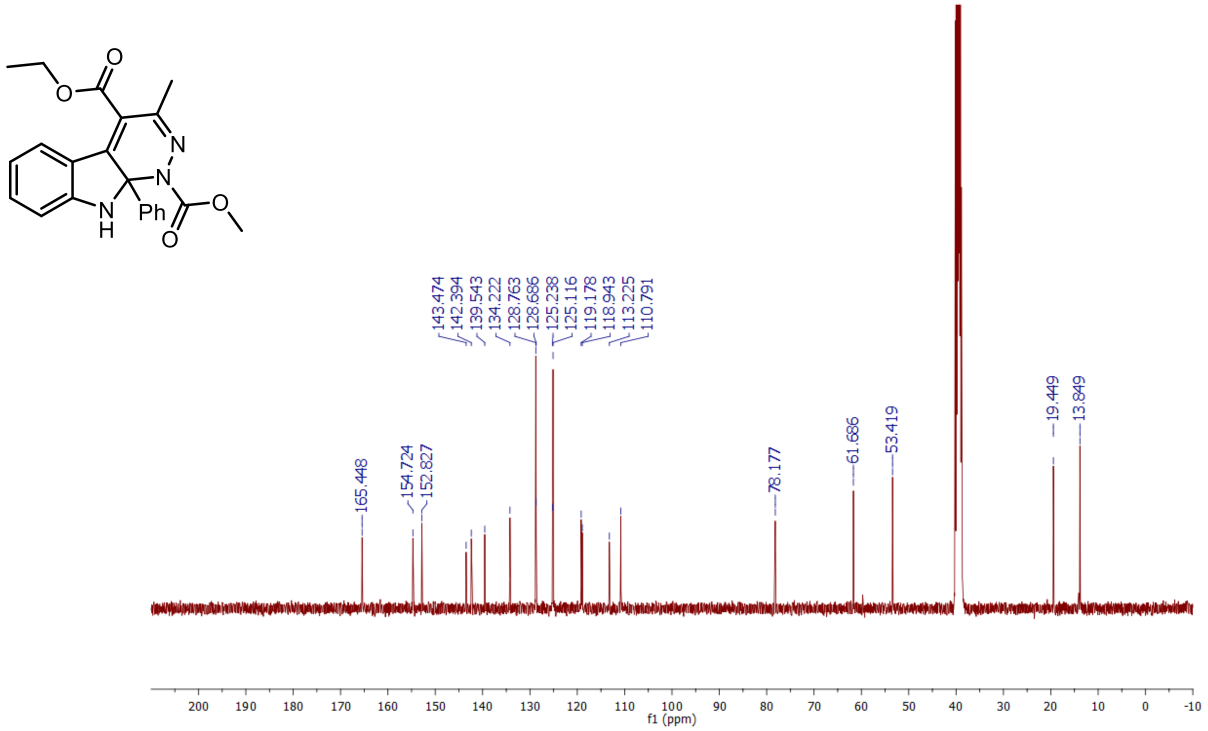

Diethyl 9a-(4-fluorophenyl)-3-methyl-9,9a-dihydro-1*H*-pyridazino[3,4-*b*]indole-1,4-dicarboxylate (3ze):

^{1}H NMR (400 MHz, DMSO-d_6) of **3ze**:

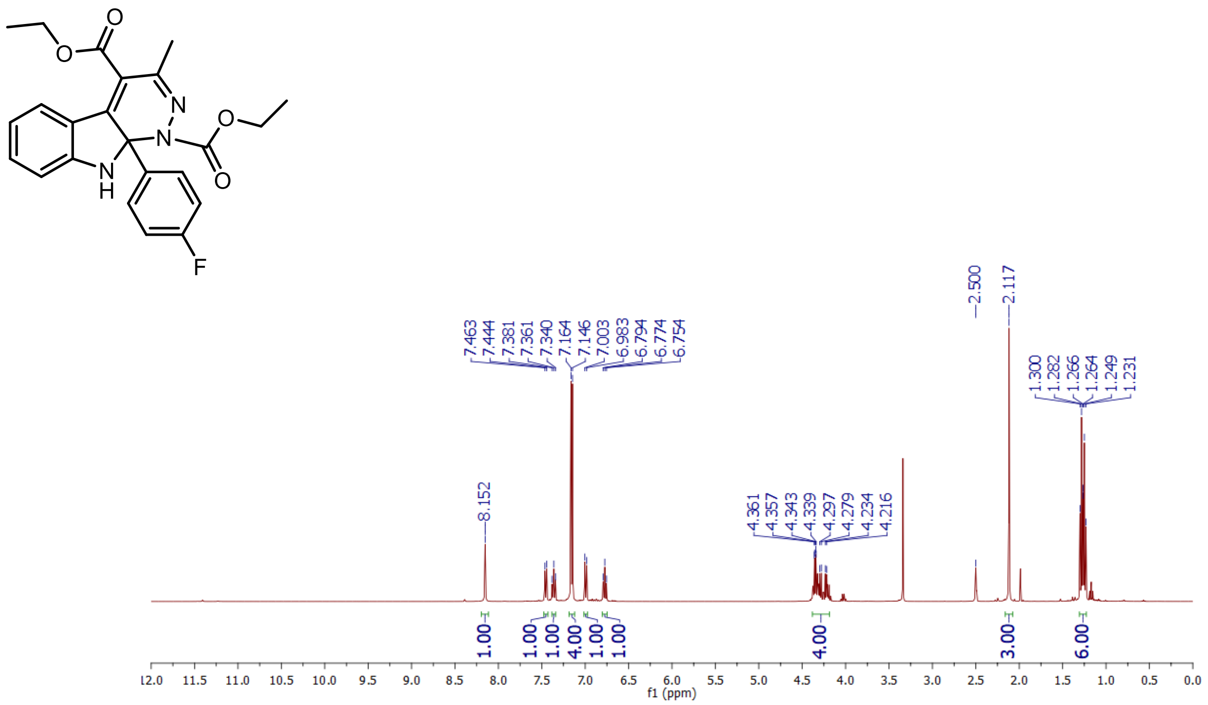

^{13}C{^{1}H} NMR (101 MHz, DMSO-d_6) of **3ze**:

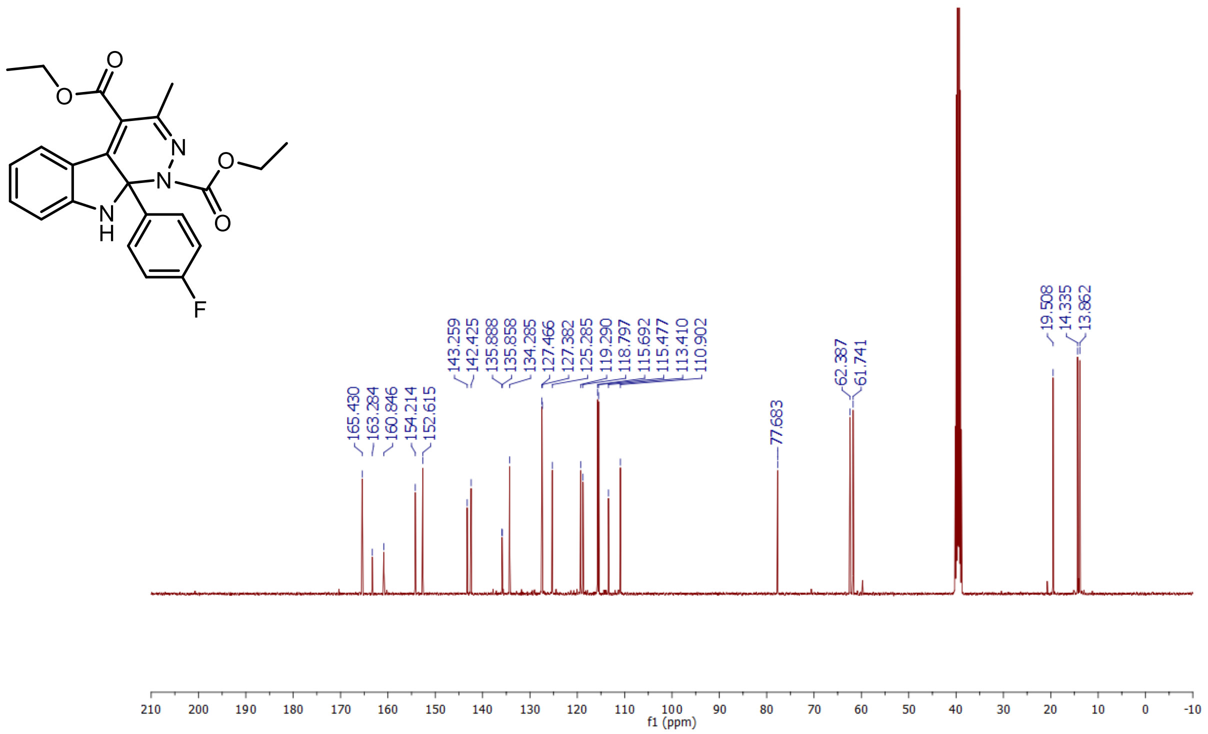

$^{19}F\{^{1}H\}$ NMR (376 MHz, DMSO-d_6) of **3ze**:

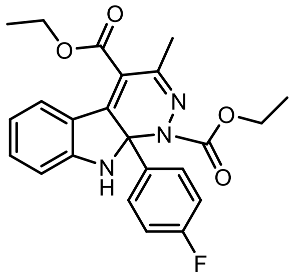

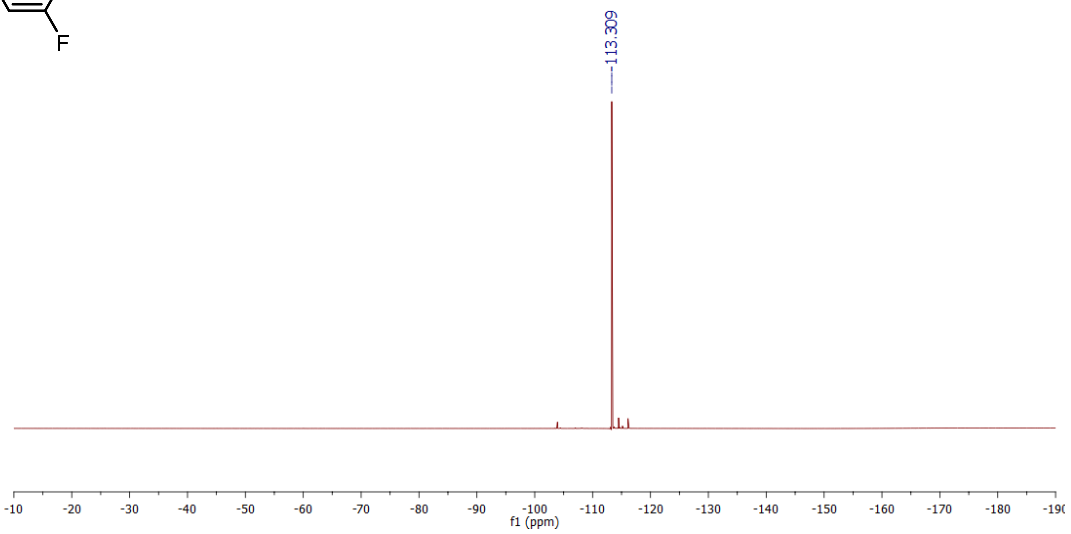

4-Ethyl 1-methyl 3-methyl-9a-(naphthalen-2-yl)-9,9a-dihydro-1*H*-pyridazino[3,4-*b*]indole-1,4-dicarboxylate (3zf):

^{1}H NMR (400 MHz, DMSO-d_6) of **3zf**:

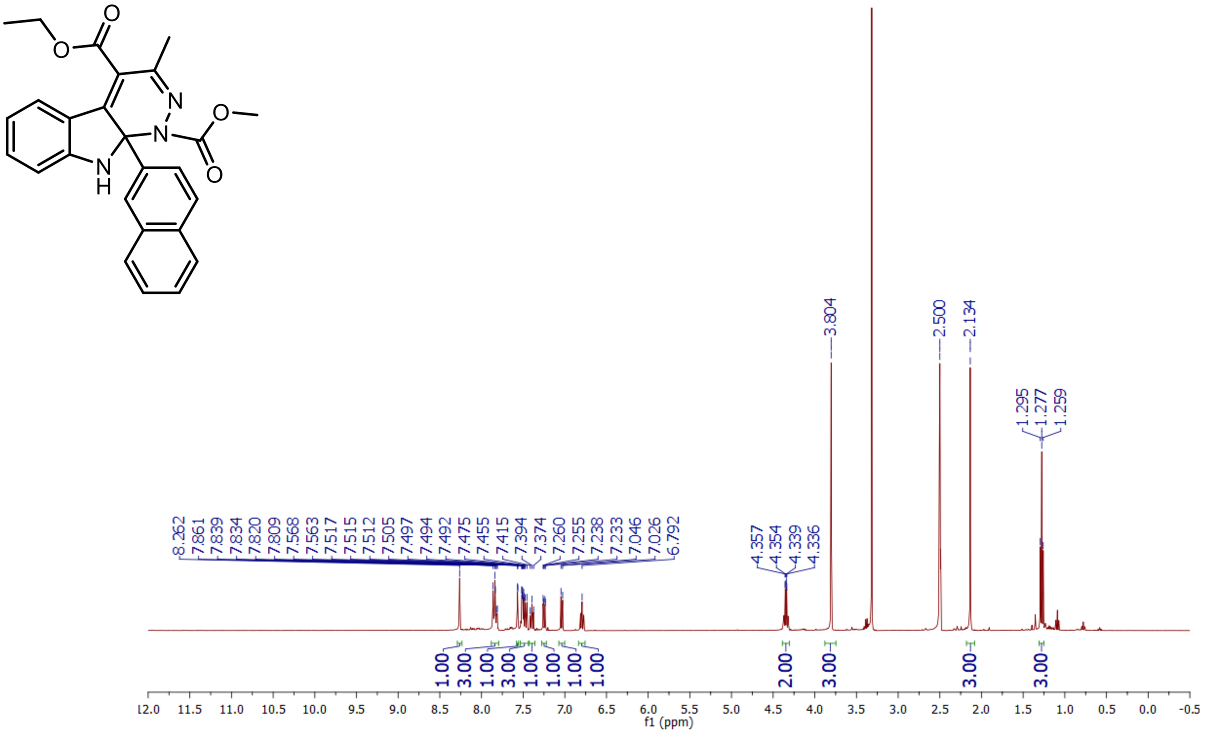

^{13}C{^{1}H} NMR (101 MHz, DMSO-d_6) of **3zf**:

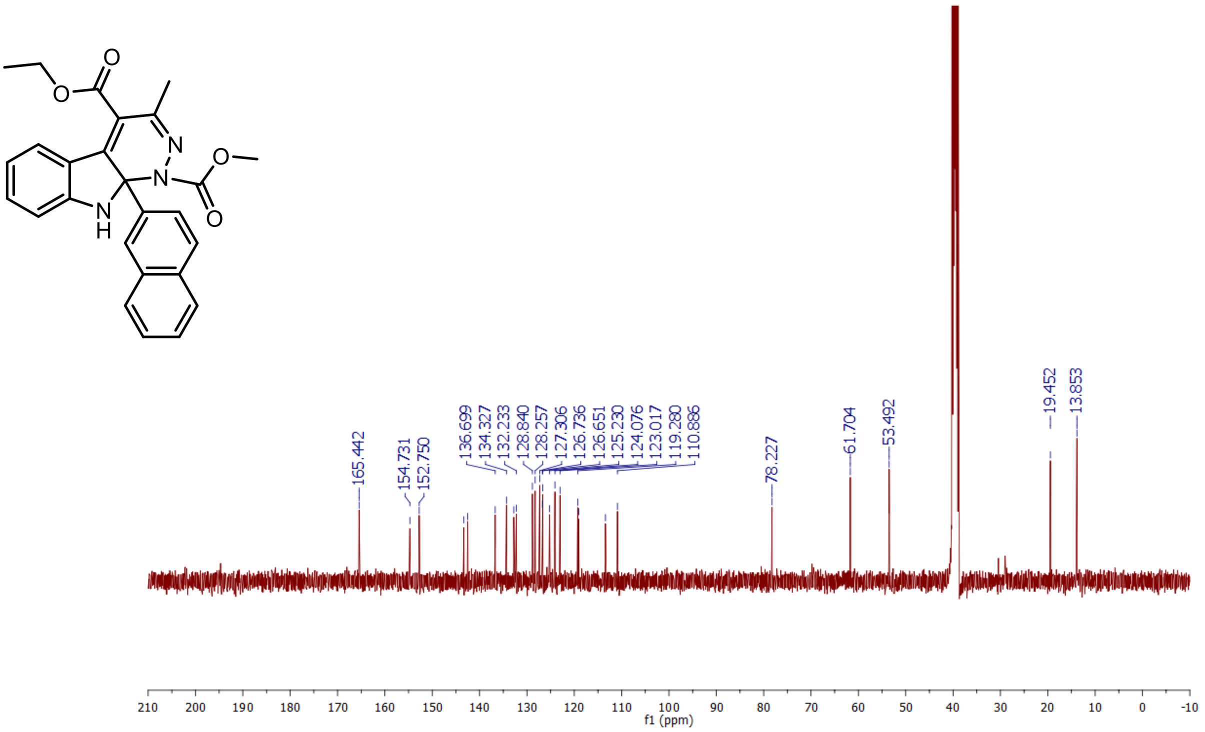

4-Ethyl 1-methyl 9-benzyl-3,9a-dimethyl-9,9a-dihydro-1*H*-pyridazino[3,4-*b*]indole-1,4-dicarboxylate (3zg):

^{1}H NMR (400 MHz, DMSO-d_6) of **3zg**:

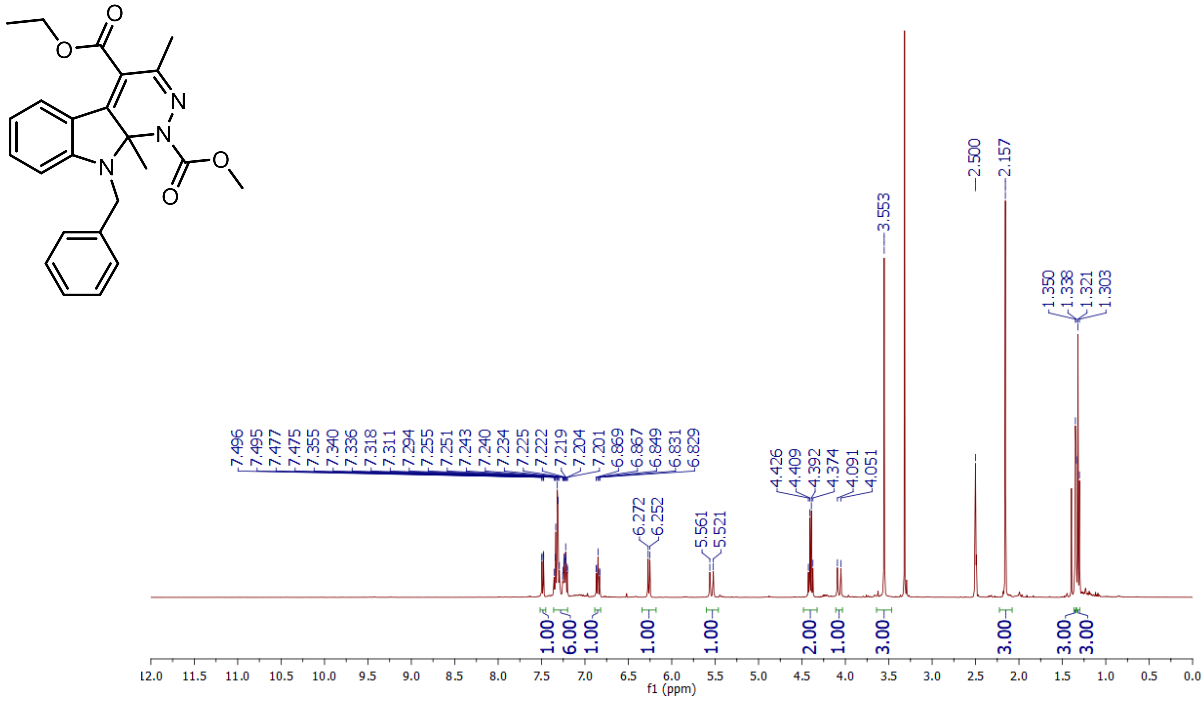

$^{13}C\{^{1}H\}$ NMR (101 MHz, DMSO-d_6) of **3zg**:

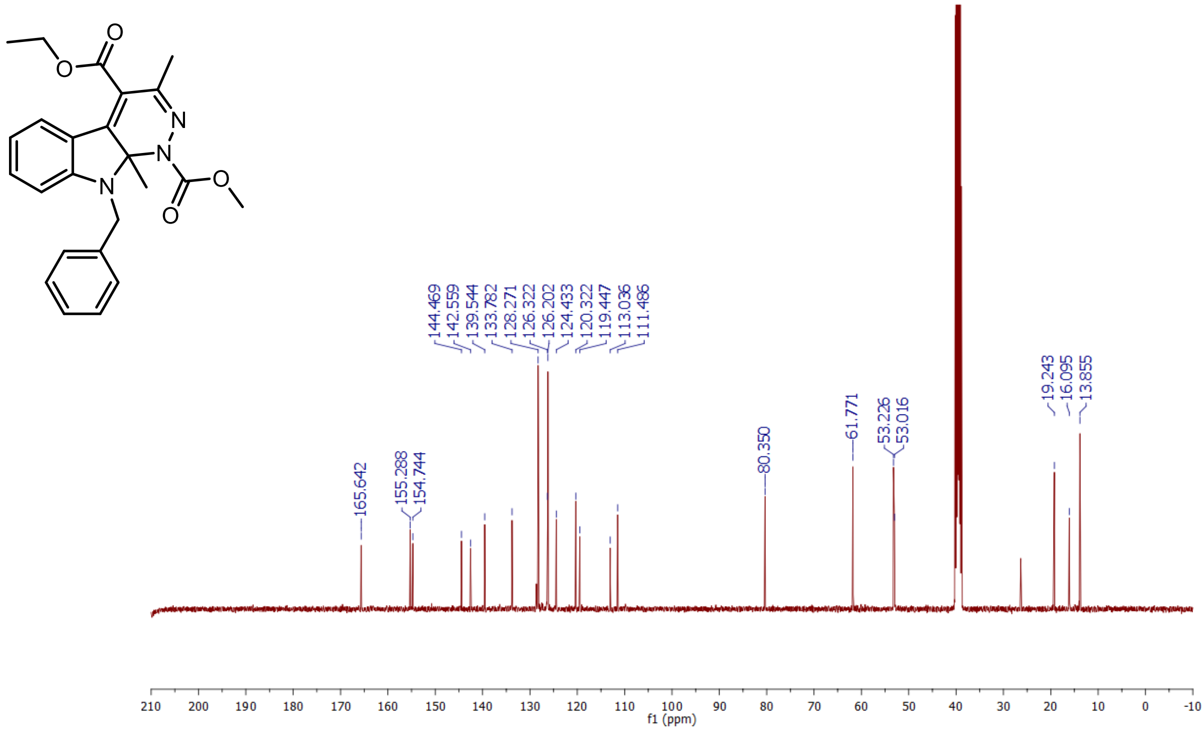

4-Ethyl 1-methyl 3,9,9a-trimethyl-9,9a-dihydro-1*H*-pyridazino[3,4-*b*]indole-1,4-dicarboxylate (3zh):

^{1}H NMR (400 MHz, DMSO-d_6) of **3zh**:

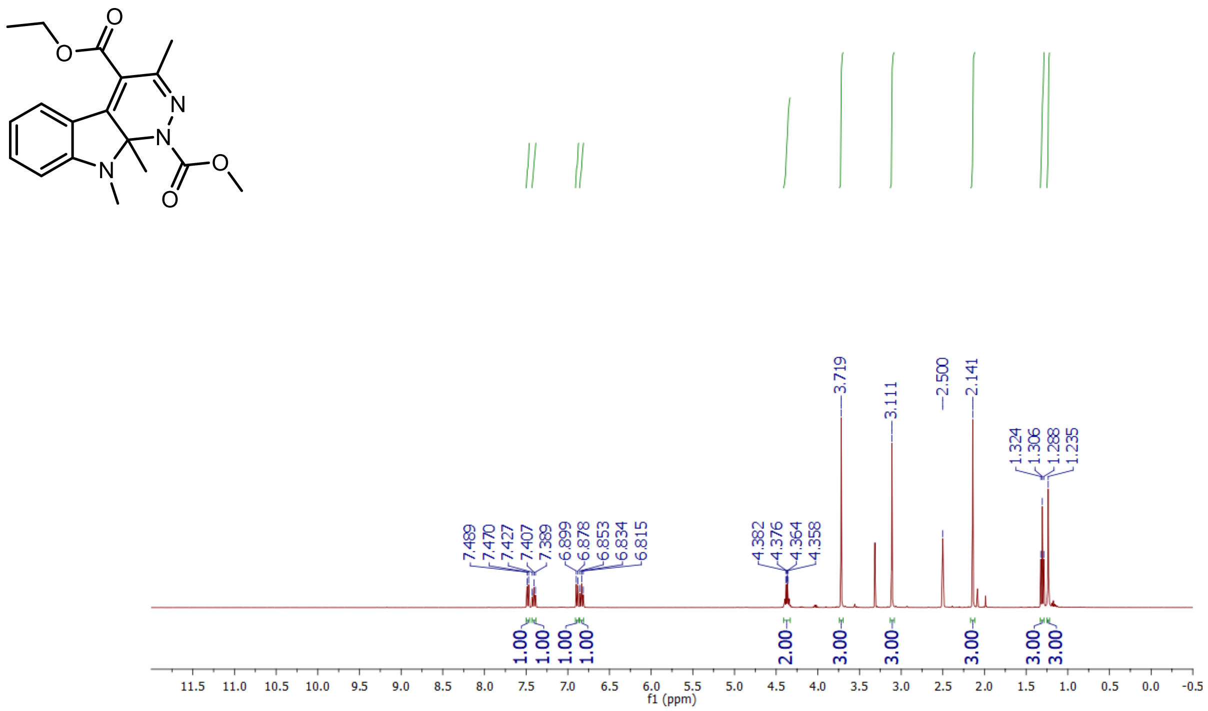

^{13}C{^{1}H} NMR (101 MHz, DMSO-d_6) of **3zh**:

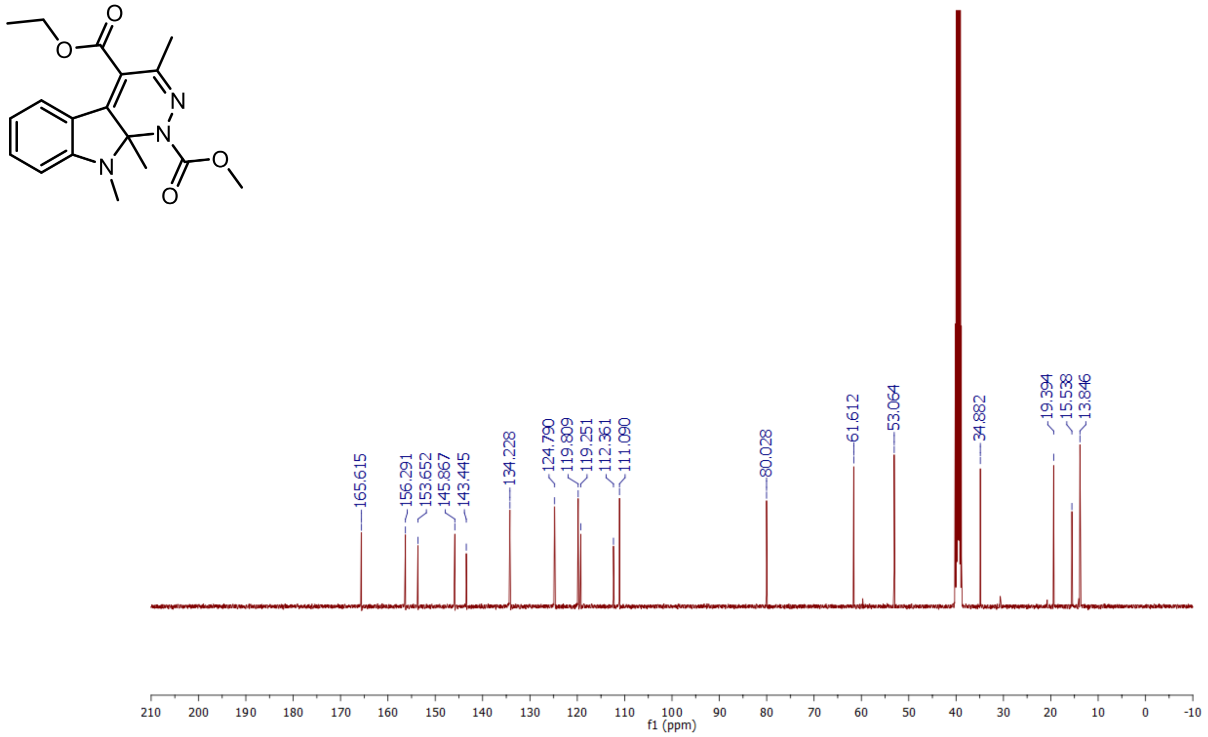

HMQC NMR (400 MHz, DMSO-d_6) of **3zh**:

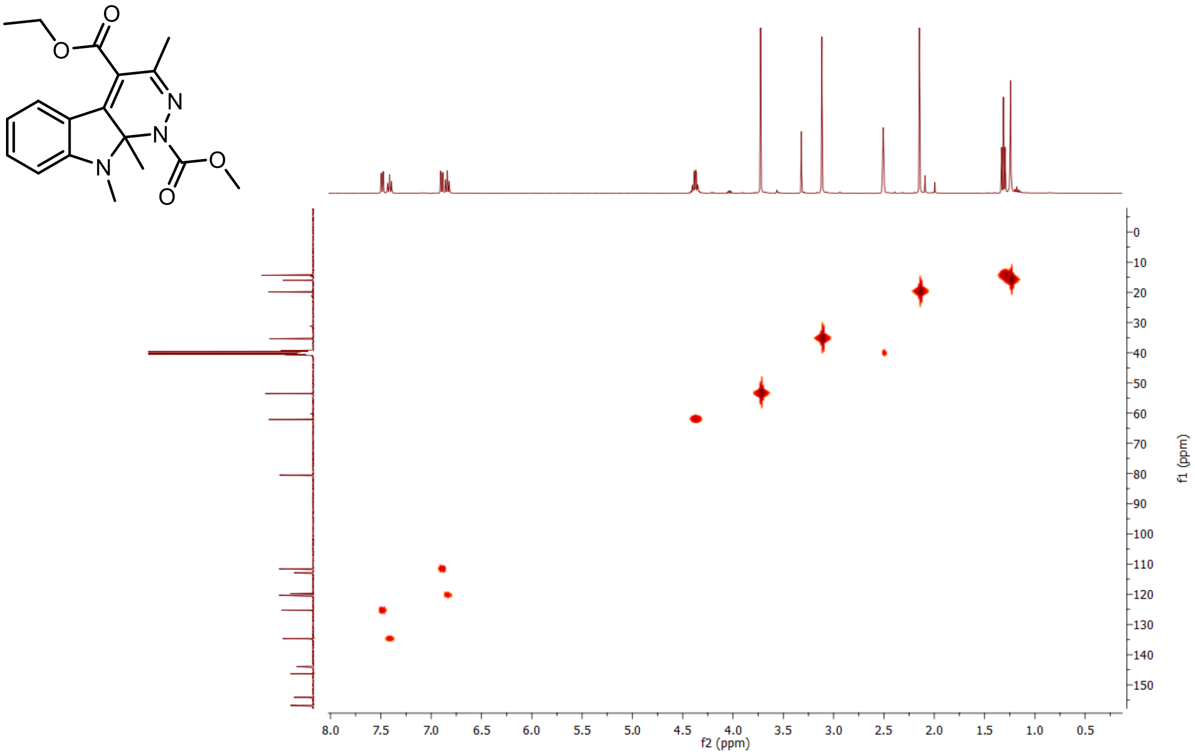

HMBC NMR (400 MHz, DMSO-d_6) of **3zh**:

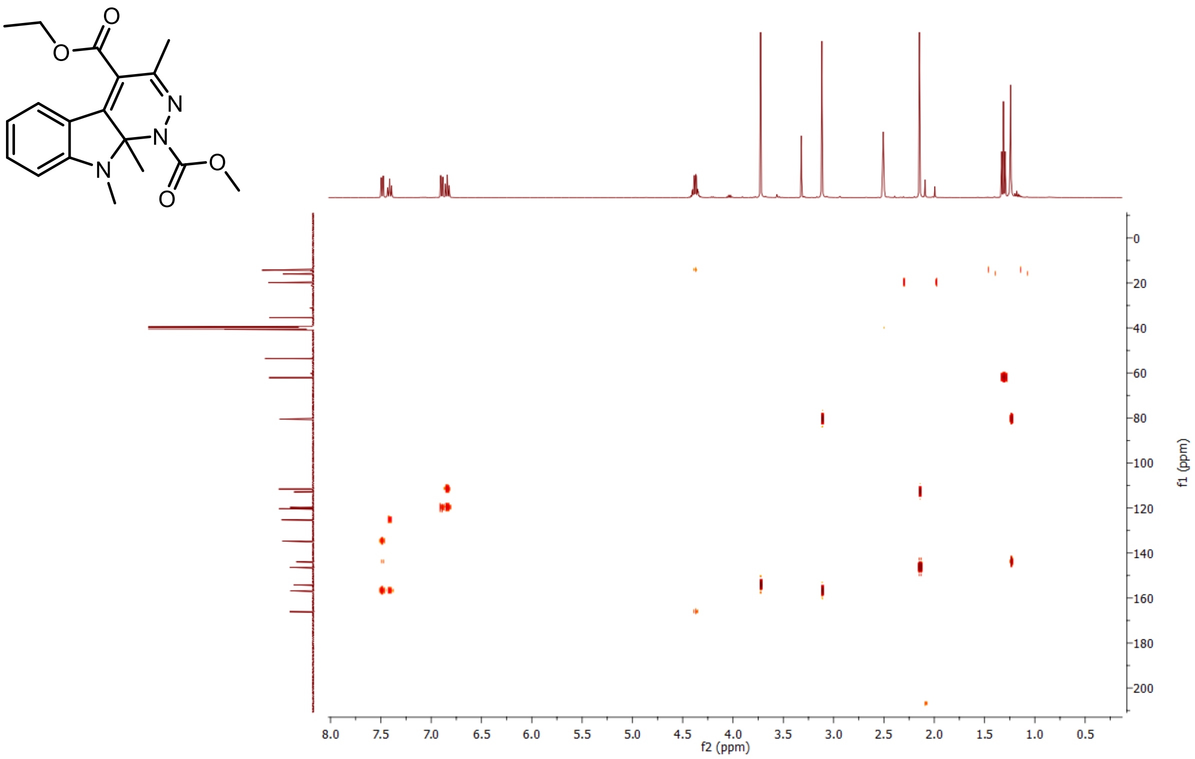

4-Ethyl 1-methyl 6,8-dichloro-3,9a-dimethyl-9,9a-dihydro-1*H*-pyridazino[3,4-*b*]indole-1,4-dicarboxylate (4a):

^{1}H NMR (400 MHz, DMSO-d_6) of **4a**:

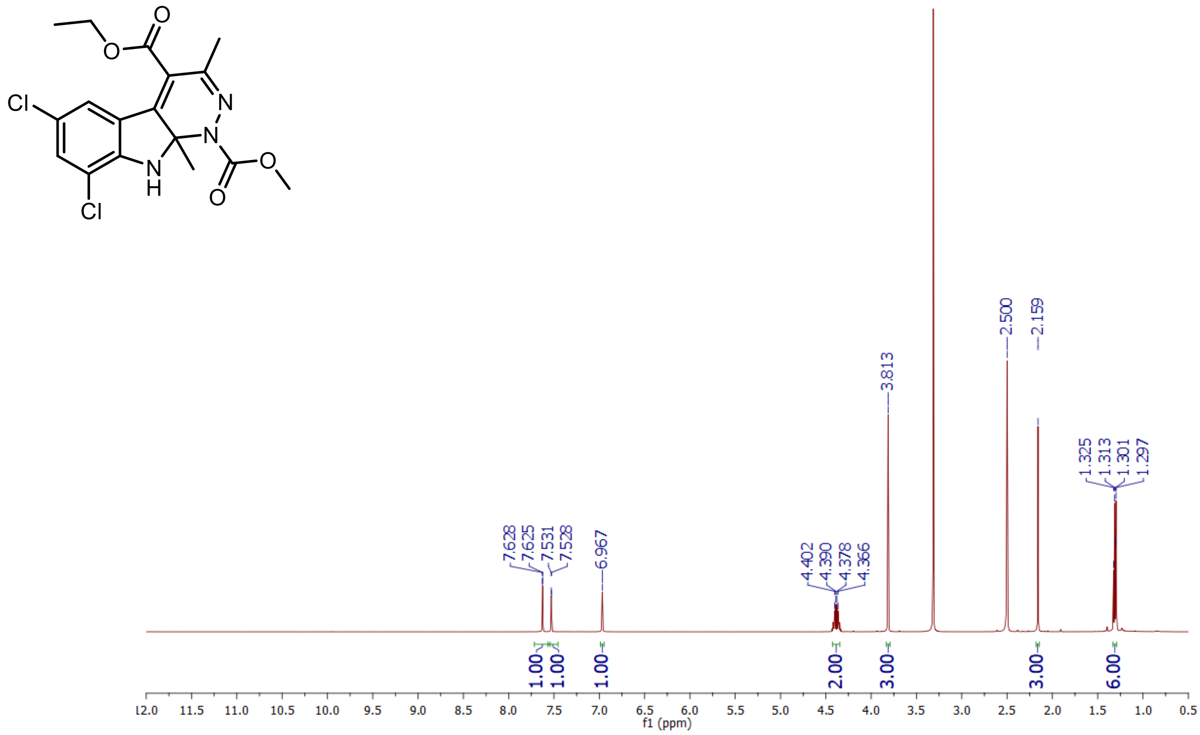

^{13}C{^{1}H} NMR (101 MHz, DMSO-d_6) of **4a**:

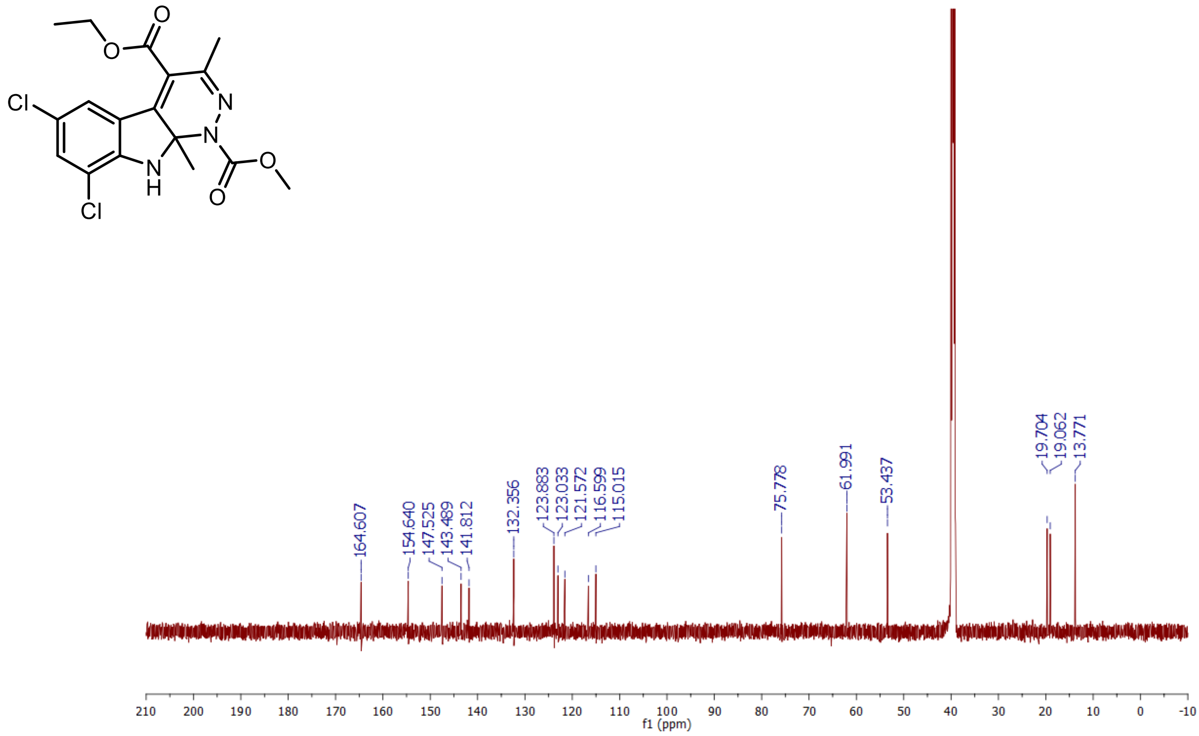

5-Methyl-4-(2-methyl-3*H*-indol-3-ylidene)-2,4-dihydro-3*H*-pyrazol-3-one (5a):

^{1}H NMR (400 MHz, DMSO-d_6) of **5a**:

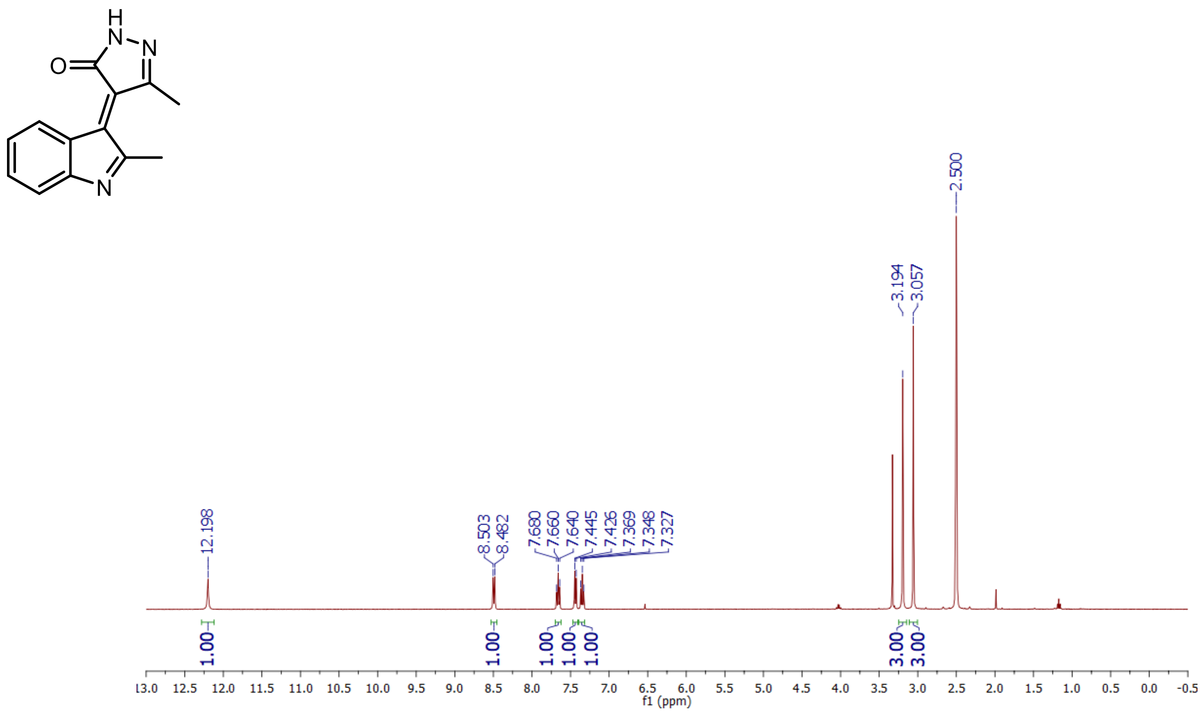

^{13}C{^{1}H} NMR (101 MHz, DMSO-d_6) of **5a**:

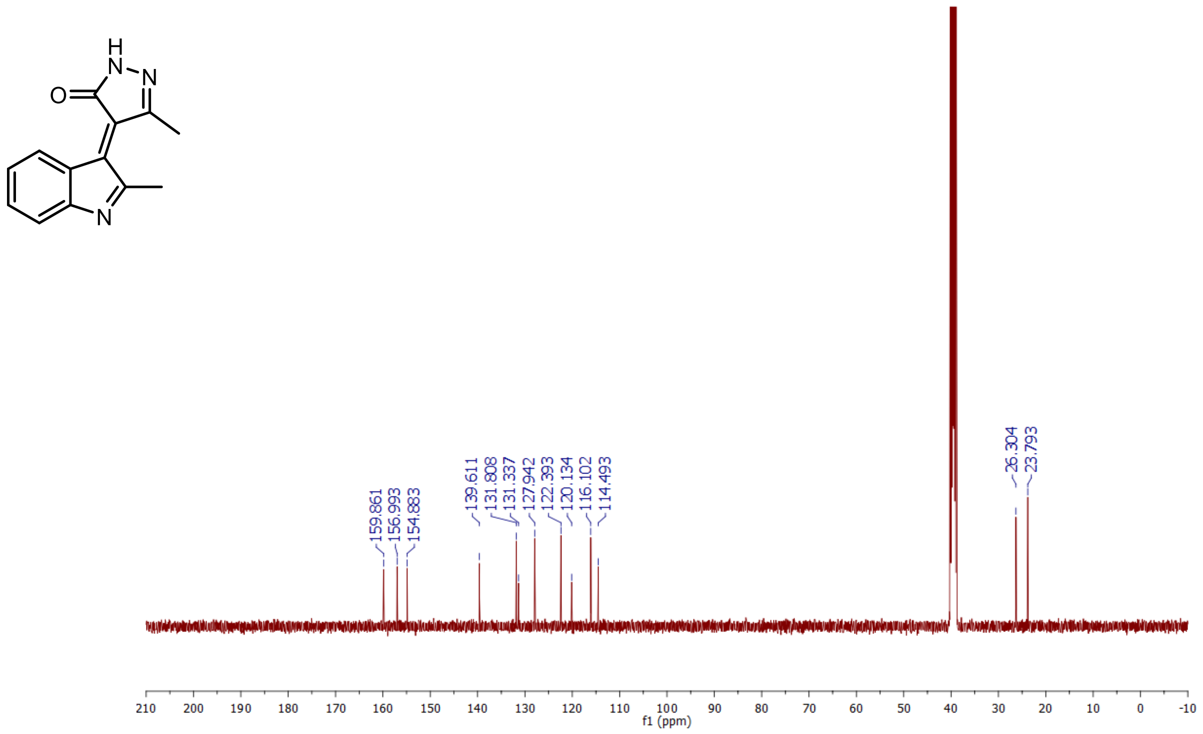

Supplement: Supplementary file 1 [file ol5c04647_si_001.pdf]
